# Supplementary material for: Low-cost, versatile, and highly reproducible microfabrication pipeline to generate 3D-printed customised cell culture devices with complex designs
Source: PLoS Biol. 2024 Mar 13;22(3):e3002503. doi: 10.1371/journal.pbio.3002503 (PMC10936828; doi:10.1371/journal.pbio.3002503)
Supplement: S1 Fig — (DOCX) [file pbio.3002503.s001.docx]

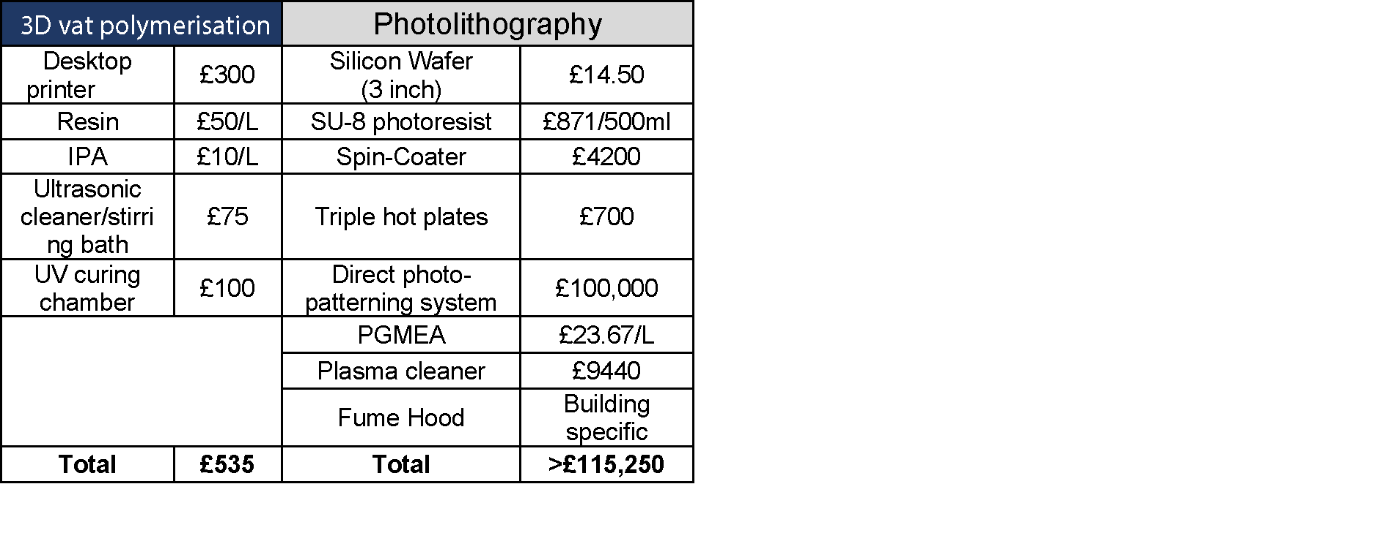


**Figure S1: Comparison of costs for 3D vat polymerization printing and photolithography with all the required equipment and materials but excluding personnel training costs.**
