# Supplementary material for: Low-cost, versatile, and highly reproducible microfabrication pipeline to generate 3D-printed customised cell culture devices with complex designs
Source: PLoS Biol. 2024 Mar 13;22(3):e3002503. doi: 10.1371/journal.pbio.3002503 (PMC10936828; doi:10.1371/journal.pbio.3002503)
Supplement: S2 Fig — (A) Chips of 6 resins were added to cultures of motor neuron progenitors pre-stained with SiR–tubulin live dye, incubated for 48 h before imaging. Representative images of SiR live dye-stained motor neurons after 48 h in culture with resins compared to 2 control wells. (B) Chips of 6 resins were treated with extra processing steps to improve biocompatibility, bake 4 h at 75°C, wash in PBS 72 h at 50°C, UV sterilise 15 min before being added to cultures of motor neuron progenitors pre-stained with SiR-tubulin cytoskeletal live dye, incubated for 48 h before imaging. Representative images of SiR live dye-stained motor neurons after 48 h in culture with treated resins compared to 2 control wells. (C) Representative time-lapse videos of SiR live dye-stained motor neurons during the first 24 h in culture with treated resin chips compared to 2 control wells. (DOCX) [file pbio.3002503.s002.docx]

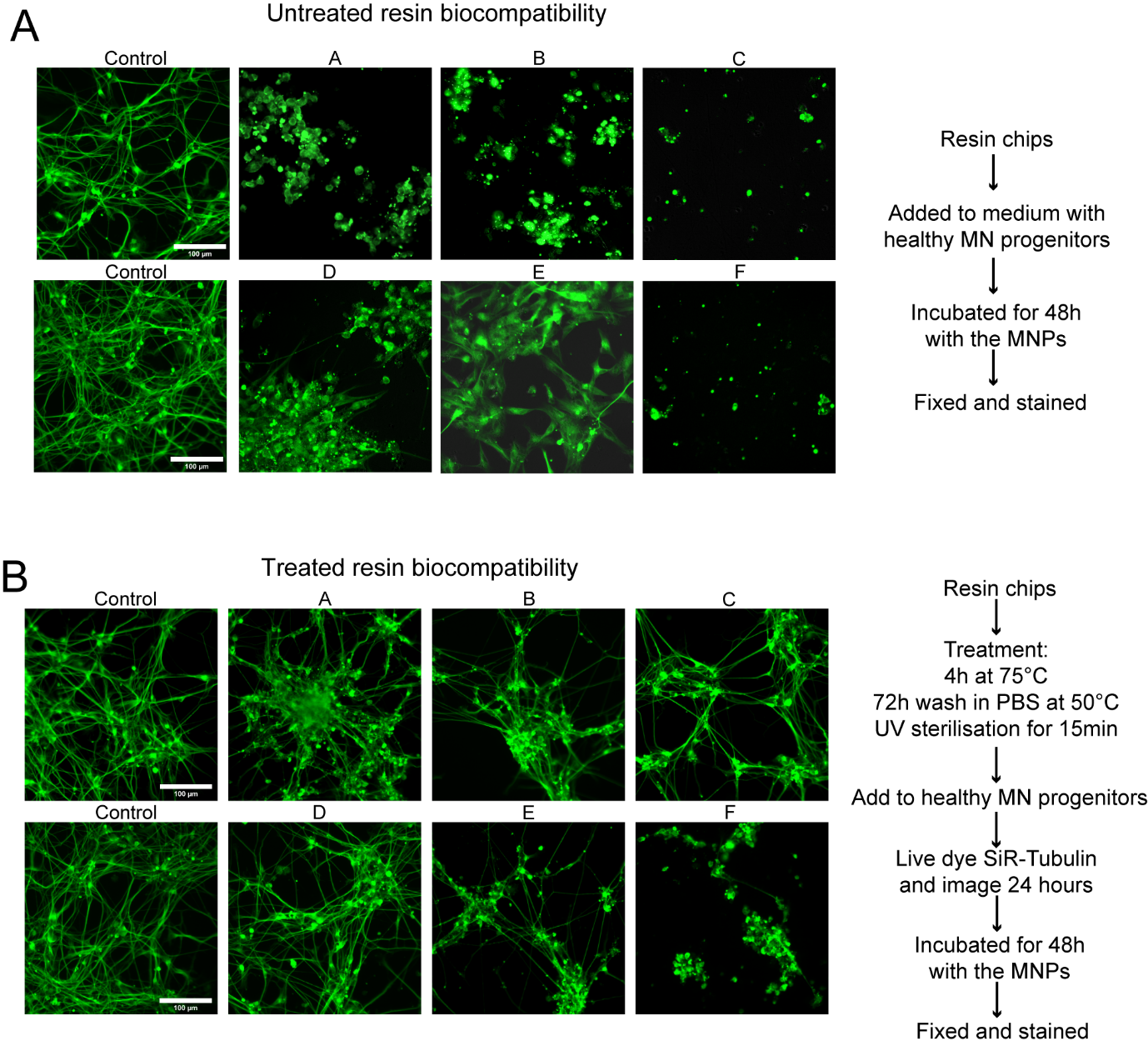


**Figure S2: SLA resins themselves are toxic with and without pre-treatment**

(A) Chips of 6 resins were added to cultures of motor neuron progenitors pre-stained with SiR – tubulin live dye, incubated for 48 hours before imaging. Representative images of SiR live dye-stained motor neurons after 48 hours in culture with resins compared to 2 control wells. (B) Chips of 6 resins were treated with extra processing steps to improve biocompatibility, bake 4 hours at 75°C, wash in PBS 72 hours at 50°C, UV sterilise 15 mins before being added to cultures of motor neuron progenitors pre-stained with SiR-tubulin cytoskeletal live dye, incubated for 48hrs before imaging. Representative images of SiR live dye-stained motor neurons after 48 hours in culture with treated resins compared to 2 control wells. (C) Representative time lapse videos of SiR live dye-stained motor neurons during the first 24 hours in culture with treated resin chips compared to 2 control wells.
