# Supplementary material for: Low-cost, versatile, and highly reproducible microfabrication pipeline to generate 3D-printed customised cell culture devices with complex designs
Source: PLoS Biol. 2024 Mar 13;22(3):e3002503. doi: 10.1371/journal.pbio.3002503 (PMC10936828; doi:10.1371/journal.pbio.3002503)
Supplement: S3 Fig — iPSC-derived MNs were plated at equal densities. Then, a 3D-printed well-sized cylinder of ortho-clear resin was added to the well and incubated for 4 days to identify the toxic effects of the biocompatible resin. The resin was either pretreated with the recommended manufacturer’s protocol or our pipeline. Cells were stained for β-3-tubulin and DAPI. (DOCX) [file pbio.3002503.s003.docx]

**
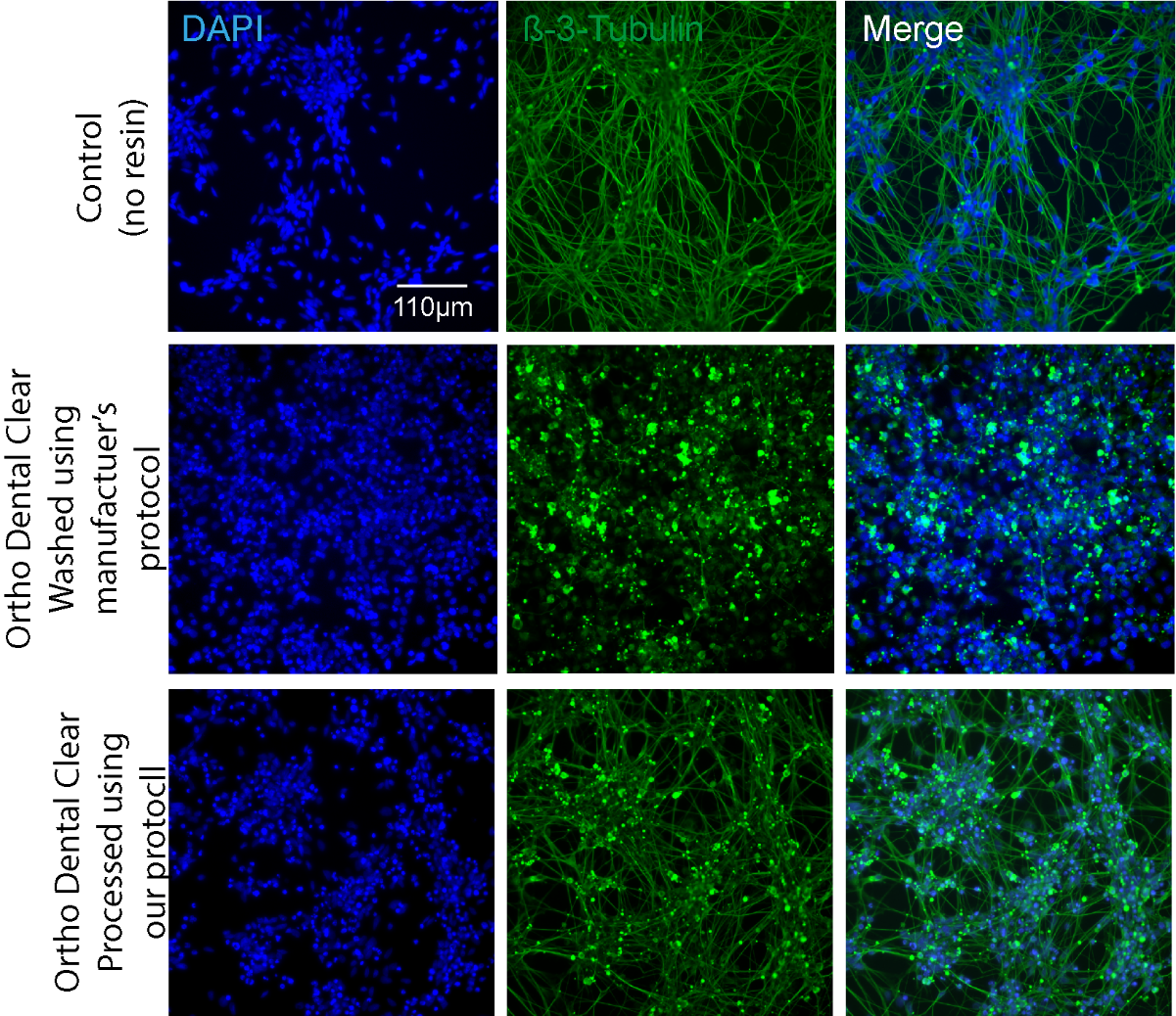
**

**Figure S3: Effects of co-culture with a biocompatible resin on iPSC-derived MNs**

iPSC-derived MNs were plated at equal densities. Then a 3D-printed well-sized cylinder of ortho-clear resin was added to the well and incubated for four days to identify the toxic effects of the biocompatible resin. The resin was either pretreated with the recommended manufacturer’s protocol or our pipeline. Cells were stained for β-3-tubulin and DAPI.
