# Supplementary material for: Low-cost, versatile, and highly reproducible microfabrication pipeline to generate 3D-printed customised cell culture devices with complex designs
Source: PLoS Biol. 2024 Mar 13;22(3):e3002503. doi: 10.1371/journal.pbio.3002503 (PMC10936828; doi:10.1371/journal.pbio.3002503)
Supplement: S4 Fig — (A) Dendrogram of the spectral similarity of PDMS casts from 3D-printed moulds fabricated with 6 commercially available resins, washed with 5 conditions (S + W = sonicate 10 min, wash 10 min, S10 = Sonicate 10 min, S20 = sonicate 20 min, W10 = Wash 10 min, W20 = Wash 20 min), either untreated or coated with airbrush (AB) and cured at 3 different temperatures (60, 75, and 90), compared to samples of uncured and cured PDMS. Replicate and print number (RxPx) for each condition. (B) Heterogeneity of PDMS cast curing from 3D-printed moulds by observation ranging from “cured,” “partially cured,” “not cured,” “damaged.” (DOCX) [file pbio.3002503.s004.docx]

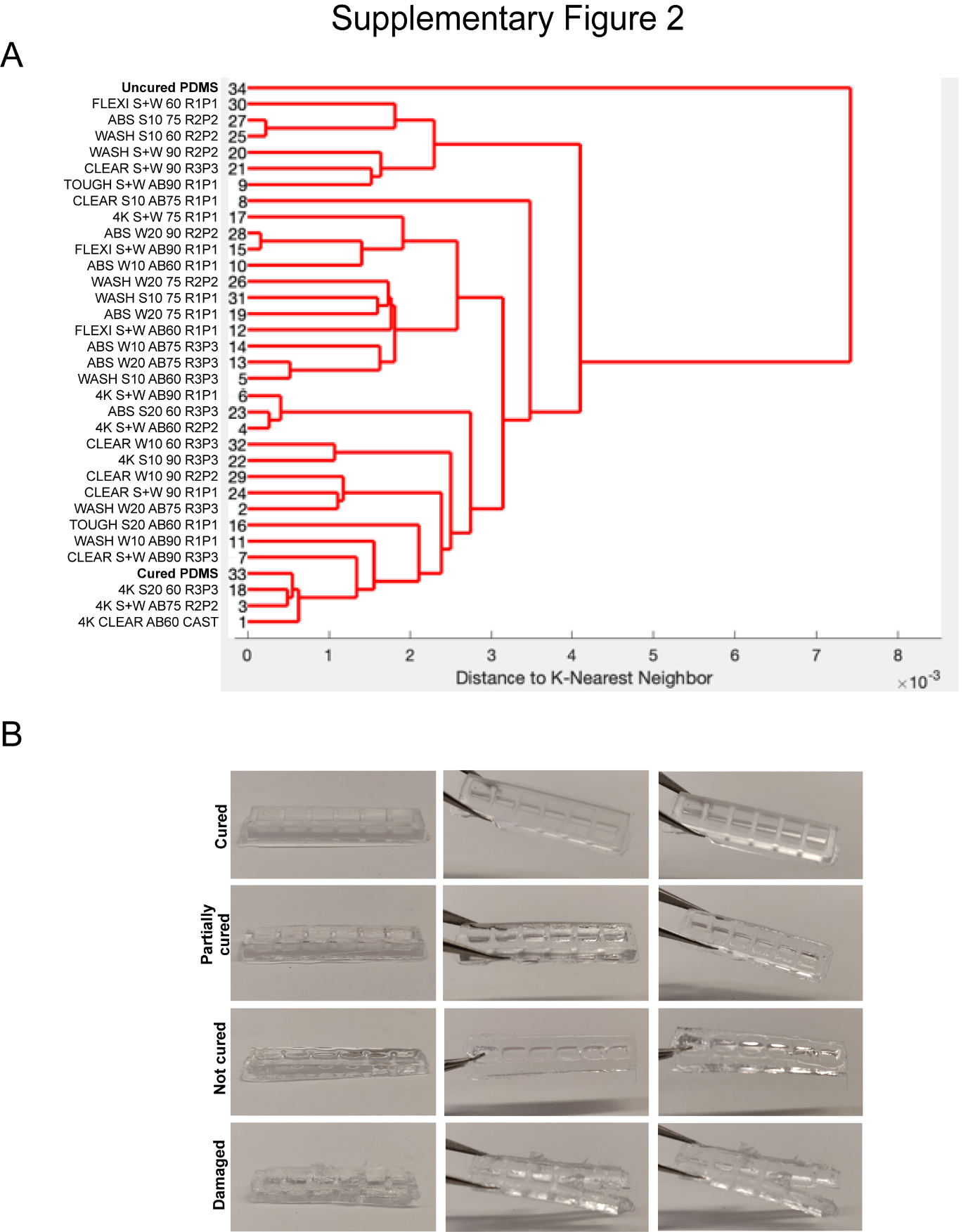


**Figure S4: Quantitation of PDMS curing on 3D printed moulds**

(A) Dendrogram of the spectral similarity of PDMS casts from 3D printed moulds fabricated with 6 commercially available resins, washed with 5 conditions (S+W = sonicate 10 mins, wash 10 mins, S10 = Sonicate 10 mins, S20 = sonicate 20 mins, W10 = Wash 10 mins, W20 = Wash 20 mins), either untreated or coated with airbrush (AB) and cured at 3 different temperatures (60), (75), (90), compared to samples of uncured and cured PDMS. Replicate and print number (RxPx) for each condition. (B) Heterogeneity of PDMS cast curing from 3D printed moulds by observation ranging from ‘cured’, ‘partially cured’, ‘not cured’, ‘damaged’.
