# Supplementary material for: Low-cost, versatile, and highly reproducible microfabrication pipeline to generate 3D-printed customised cell culture devices with complex designs
Source: PLoS Biol. 2024 Mar 13;22(3):e3002503. doi: 10.1371/journal.pbio.3002503 (PMC10936828; doi:10.1371/journal.pbio.3002503)
Supplement: S5 Fig — Spectra of samples taken after incubating PDMS devices fabricated in SOL3D moulds in water at 37°C for 72 h, to mimic the conditions of the biological experiments, compared to a control consisting of water maintained at 37°C for 72 h within the same incubator and the technical control for the analysis employing only MALDI matrix. The comparison shows no detectable leachates in water incubated with the SOL3D PDMS devices, with a profile essentially comparable to what observed in the water only control. (DOCX) [file pbio.3002503.s005.docx]

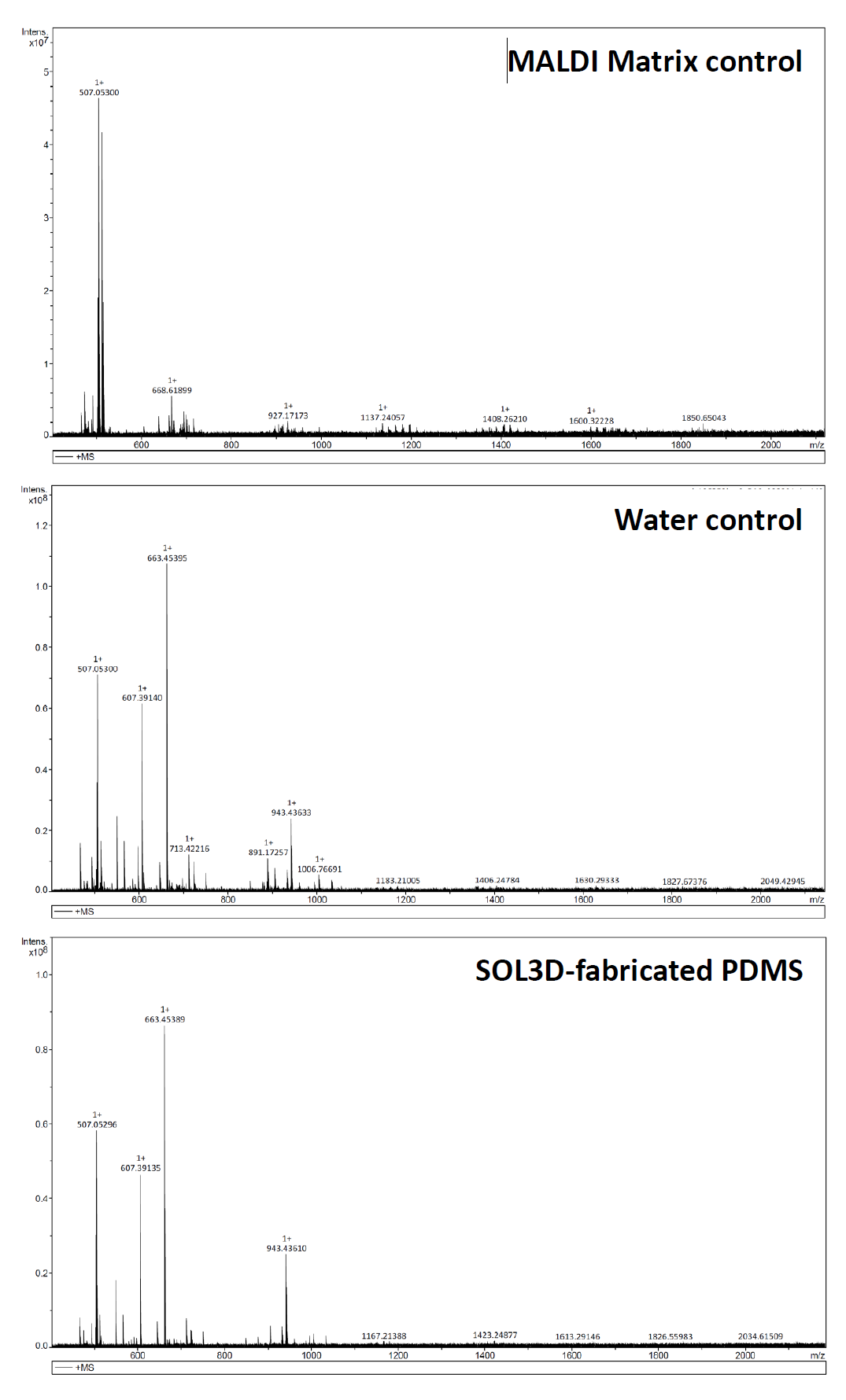


**Figure S5: MALDI-TOF analysis of leachates from SOL3D PDMS devices**

Spectra of samples taken after incubating PDMS devices fabricated in SOL3D molds in water at 37 °C for 72 h, to mimic the conditions of the biological experiments, compared to a control consisting of water maintained at 37 °C for 72 h within the same incubator and the technical control for the analysis employing only MALDI matrix. The comparison shows no detectable leachates in water incubated with the SOL3D PDMS devices, with a profile essentially comparable to what observed in the water only control
