# Supplementary material for: Low-cost, versatile, and highly reproducible microfabrication pipeline to generate 3D-printed customised cell culture devices with complex designs
Source: PLoS Biol. 2024 Mar 13;22(3):e3002503. doi: 10.1371/journal.pbio.3002503 (PMC10936828; doi:10.1371/journal.pbio.3002503)
Supplement: S6 Fig — (A) Representative β-III Tubulin stained differentiated motor neurons cultured on a flat substrate. (B) Brightfield images of differentiated motor neurons with a PDMS cast from a 3D-printed mould in the culture medium. (C) β-III Tubulin stained motor neuron long axons seeded on PDMS microfabricated substrate. (D) Snapshots from 60-h time-lapse of SiR–tubulin live dye stained motor neuron differentiation on PDMS substrate cast in a 3D-printed mould. (DOCX) [file pbio.3002503.s006.docx]

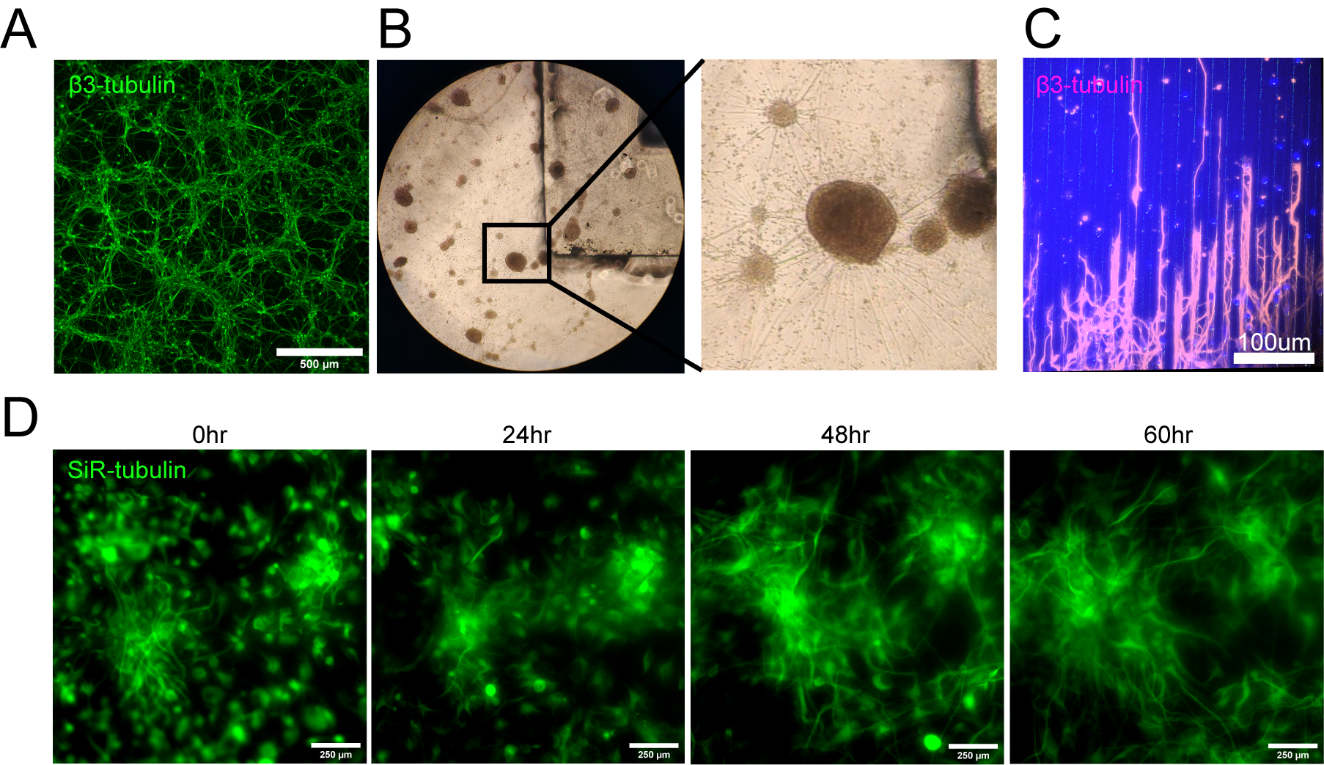


**Figure S6: Biocompatibility of PDMS cast in 3D printed moulds with cells (timelapse and long axons)**

(A) Representative β-III Tubulin stained differentiated motor neurons cultured on a flat substrate. (B) Brightfield images of differentiated motor neurons with a PDMS cast from a 3D printed mould in the culture medium**.** (C) β-III Tubulin stained motor neuron long axons seeded on PDMS microfabricated substrate**.** (D) Snapshots from 60-hour time-lapse of SiR – tubulin live dye stained motor neuron differentiation on PDMS substrate cast in a 3D printed mould.
