# Supplementary material for: Low-cost, versatile, and highly reproducible microfabrication pipeline to generate 3D-printed customised cell culture devices with complex designs
Source: PLoS Biol. 2024 Mar 13;22(3):e3002503. doi: 10.1371/journal.pbio.3002503 (PMC10936828; doi:10.1371/journal.pbio.3002503)
Supplement: S7 Fig — (A) Representative SEM image of a resin A print with airbrushed enamel paint. (B) Analysis of paint layer thickness on 3D vat printed moulds. (C–F) Optical profiling of a 3D print (C) painted with enamel, shows a surface roughness that is variable around 3 μm (D) a flat silicon wafer shows variability in the nanometre scale (E, F) a non-painted 3D print showing a small variation based on the pixel size of the screen, with 1 μm variability. (DOCX) [file pbio.3002503.s007.docx]

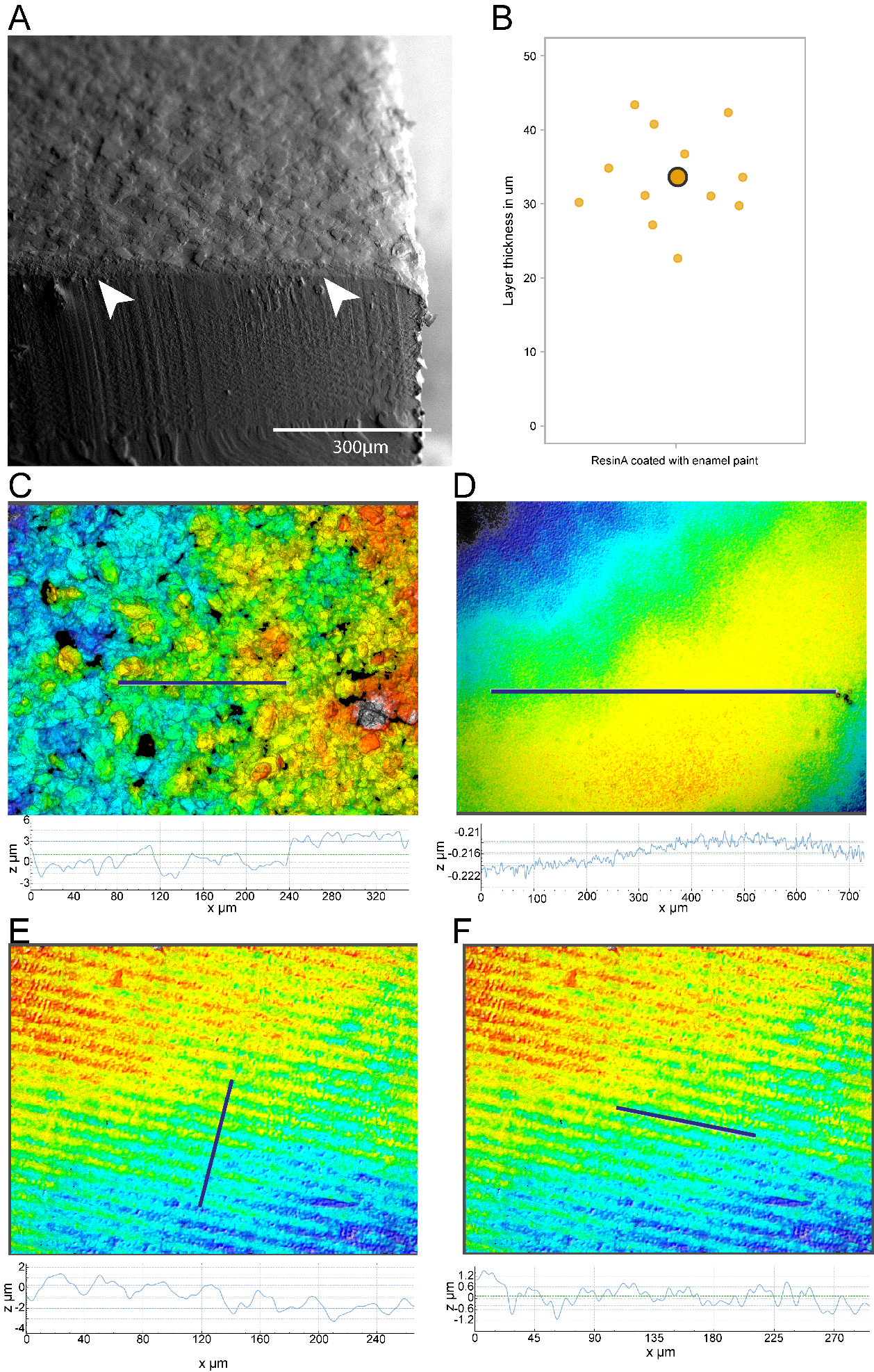


**Figure S7: Analysis of paint layer thickness and surface roughness**

(A) Representative SEM image of a resin A print with airbrushed enamel paint (B) Analysis of paint layer thickness on 3D vat printed moulds (C, D , E, F) Optical profiling of a 3D print (C) painted with enamel, shows a surface roughness that is variable around 3um (D) a flat silicon wafer shows variability in the nanometer scale (E, F) a non-painted 3D print showing a small variation based on the pixel size of the screen, with 1um variability.
