# Supplementary material for: Low-cost, versatile, and highly reproducible microfabrication pipeline to generate 3D-printed customised cell culture devices with complex designs
Source: PLoS Biol. 2024 Mar 13;22(3):e3002503. doi: 10.1371/journal.pbio.3002503 (PMC10936828; doi:10.1371/journal.pbio.3002503)

**Figure S8: PDMS stencil devices manufactured using photolithography**

(A) Dimensions of the stencil-like device with photolithography (B) Schematic overview of the seeding strategy with stencil-like devices (C) Representative image of a stencil-like device with pockets for cell seeding (arrow) (D) Demonstration of cell seeding using food coloring as a ‘single-cell suspension’. The liquid was manually pipetted on the pockets of the device (arrow). Limited volume can be used. (E) Representative brightfield image of single motoneurons seeded using a stencil device manufactured using photolithography and a schematic showing the seeding process as well as the position of the cells. (F) Representative brightfield image of single motoneurons seeded using a plating device manufactured using SOL3D and a schematic showing the seeding process as well as the position of the cells.


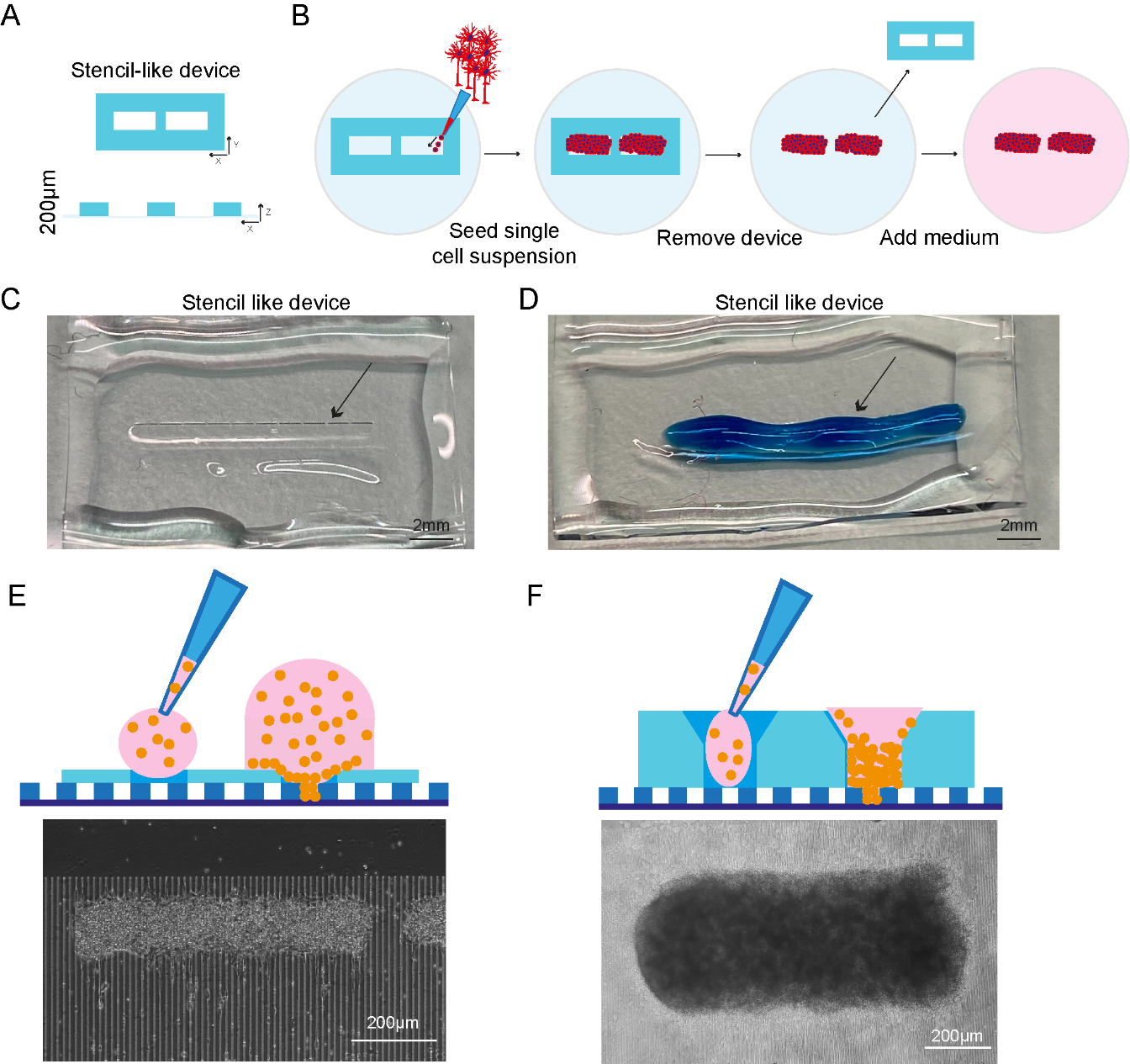

Supplement: S8 Fig — (A) Dimensions of the stencil-like device with photolithography. (B) Schematic overview of the seeding strategy with stencil-like devices. (C) Representative image of a stencil-like device with pockets for cell seeding (arrow). (D) Demonstration of cell seeding using food colouring as a “single-cell suspension.” The liquid was manually pipetted on the pockets of the device (arrow). Limited volume can be used. (E) Representative Brightfield image of single motoneurons seeded using a stencil device manufactured using photolithography and a schematic showing the seeding process as well as the position of the cells. (F) Representative Brightfield image of single motoneurons seeded using a plating device manufactured using SOL3D and a schematic showing the seeding process as well as the position of the cells. (DOCX) [file pbio.3002503.s008.docx]
