# Supplementary material for: Low-cost, versatile, and highly reproducible microfabrication pipeline to generate 3D-printed customised cell culture devices with complex designs
Source: PLoS Biol. 2024 Mar 13;22(3):e3002503. doi: 10.1371/journal.pbio.3002503 (PMC10936828; doi:10.1371/journal.pbio.3002503)
Supplement: S9 Fig — (A) Representative optical profiles from PDMS casts demoulded from a single airbrushed 3D-printed mould and a single microfabricated mould. (B) Representation of 5 ROI selection for quantification of surface roughness on PDMS casts. (C) Quantitation of surface roughness of PDMS casts from the same device over time (25 simultaneous casts) between an airbrushed 3D-printed mould and a microfabricated mould. (DOCX) [file pbio.3002503.s009.docx]

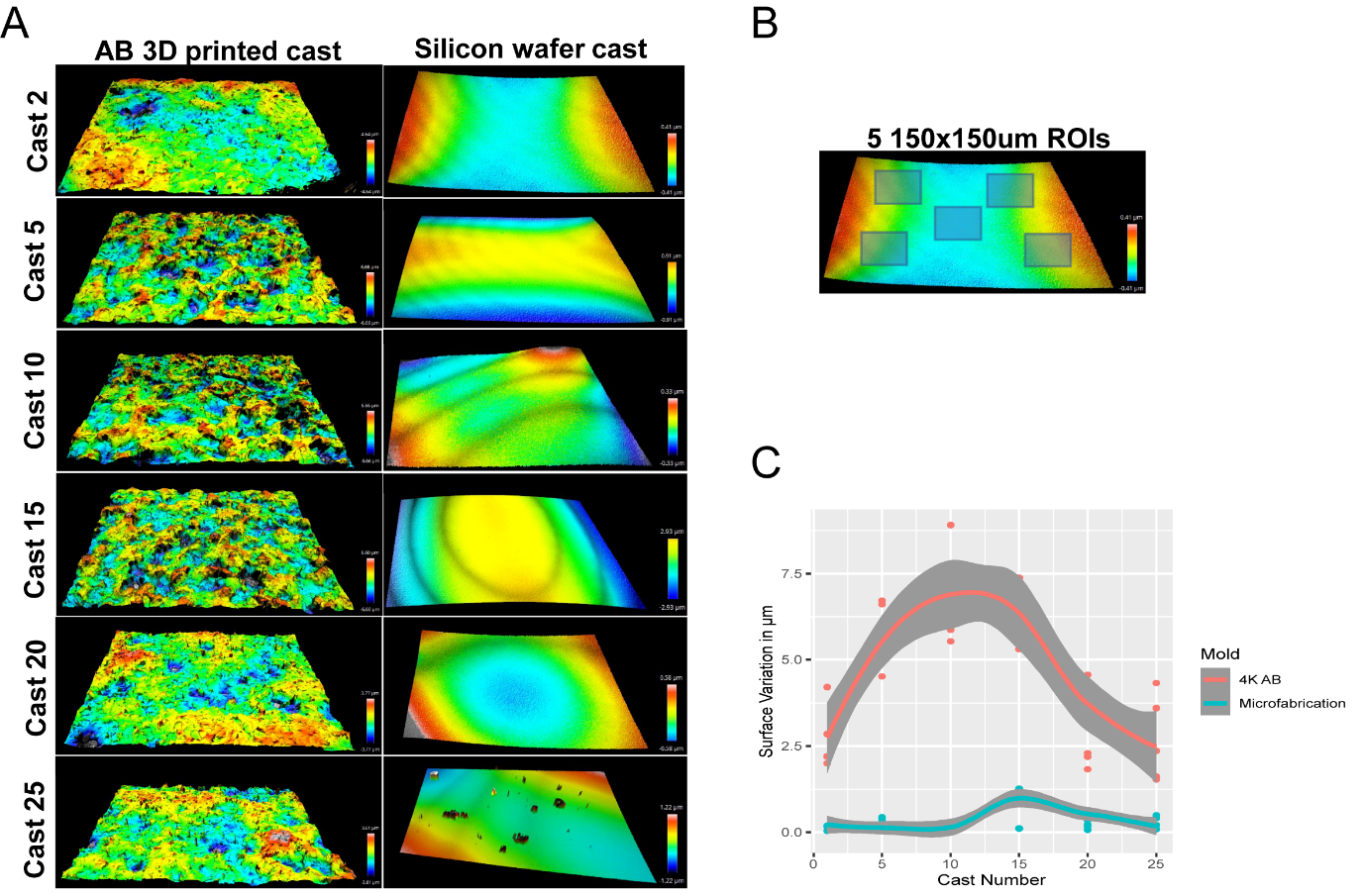


**Figure S9: The surface of 3D prints is rough**

(A) Representative optical profiles from PDMS casts demoulded from a single airbrushed 3D printed mould and a single microfabricated mould. (B) Representation of 5 ROI selection for quantification of surface roughness on PDMS casts**.** (C) Quantitation of surface roughness of PDMS casts from the same device over time (25 simultaneous casts) between an airbrushed 3D printed mould and a microfabricated mould.
