# Supplementary material for: Low-cost, versatile, and highly reproducible microfabrication pipeline to generate 3D-printed customised cell culture devices with complex designs
Source: PLoS Biol. 2024 Mar 13;22(3):e3002503. doi: 10.1371/journal.pbio.3002503 (PMC10936828; doi:10.1371/journal.pbio.3002503)
Supplement: S10 Fig — (A) Schematic (top) and representative image (bottom) of clamping approach to ensure fluid seal when devices are placed on PDMS microgroove substrates. (B) Comparison of liquid seal integrity of clamping strategy (top) compared to open curing (bottom) on PDMS microgroove substrate with different well sizes and shapes using dyed liquid. Successful sealing of devices cast with a glass cover (Green zoom). Dye spreads throughout device and grooves using open cured (Red zoom). Arrows highlight liquid spreading. (DOCX) [file pbio.3002503.s010.docx]

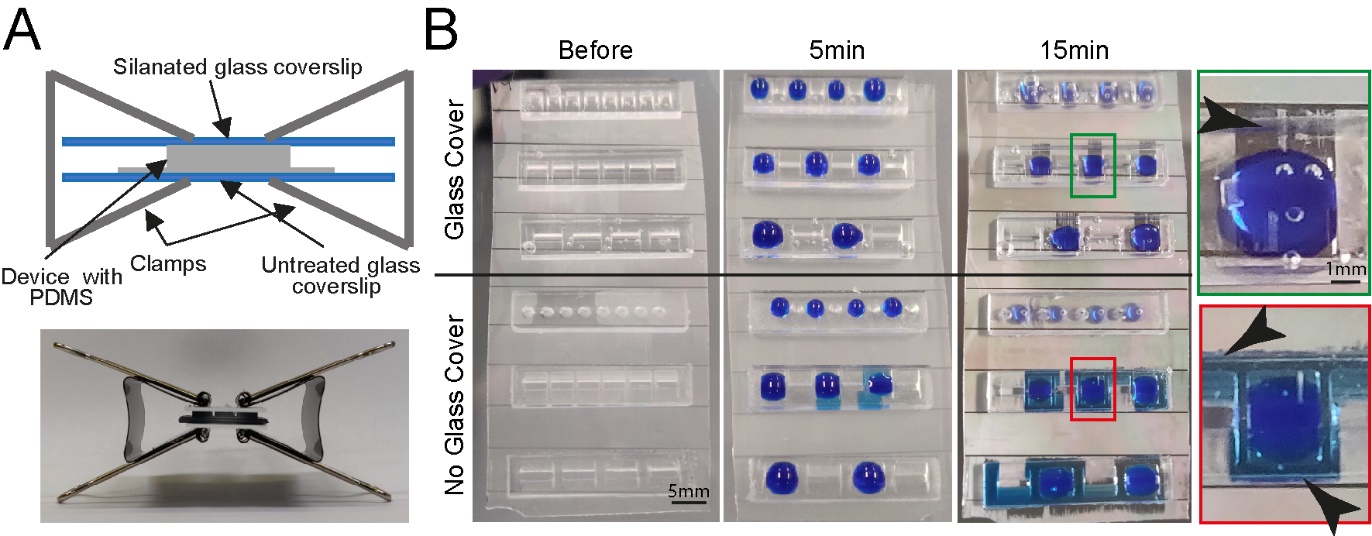


**Figure S10:** **Fluidic seal for cell plating devices**

(A) Schematic (top) and representative image (bottom) of clamping approach to ensure fluid seal when devices are placed on PDMS microgroove substrates. (B) Comparison of liquid seal integrity of clamping strategy (top) compared to open curing (bottom) on PDMS microgroove substrate with different well sizes and shapes using dyed liquid. Successful sealing of devices cast with a glass cover (**Green zoom)**. Dye spreads throughout device and grooves using open cured (**Red zoom)**. Arrows highlight liquid spreading.
