# Supplementary material for: Low-cost, versatile, and highly reproducible microfabrication pipeline to generate 3D-printed customised cell culture devices with complex designs
Source: PLoS Biol. 2024 Mar 13;22(3):e3002503. doi: 10.1371/journal.pbio.3002503 (PMC10936828; doi:10.1371/journal.pbio.3002503)
Supplement: S11 Fig — (A) Graph comparing X and Y dimensions of 3D printed constructs to CAD specifications in a single device with well dimensions ranging from 600 μm × 1,000 μm to 50 μm × 1,000 μm. (B) Graph comparing X and Y dimensions of 300 μm × 1,000 μm features on 3D printed constructs to CAD specifications for 6 commercially available resins printed on two 3D printers at manufacturer default settings with a 50 μm layer thickness. (C) Graph comparing the actual layer thickness of 3D printed constructs to CAD specifications for 6 commercially available resins printed on 2 SLA 3D printers at manufacturer default settings with a 50 μm layer thickness. Data points for all graphs can be found in file S11A–S11C Data in S1 Data. (DOCX) [file pbio.3002503.s011.docx]

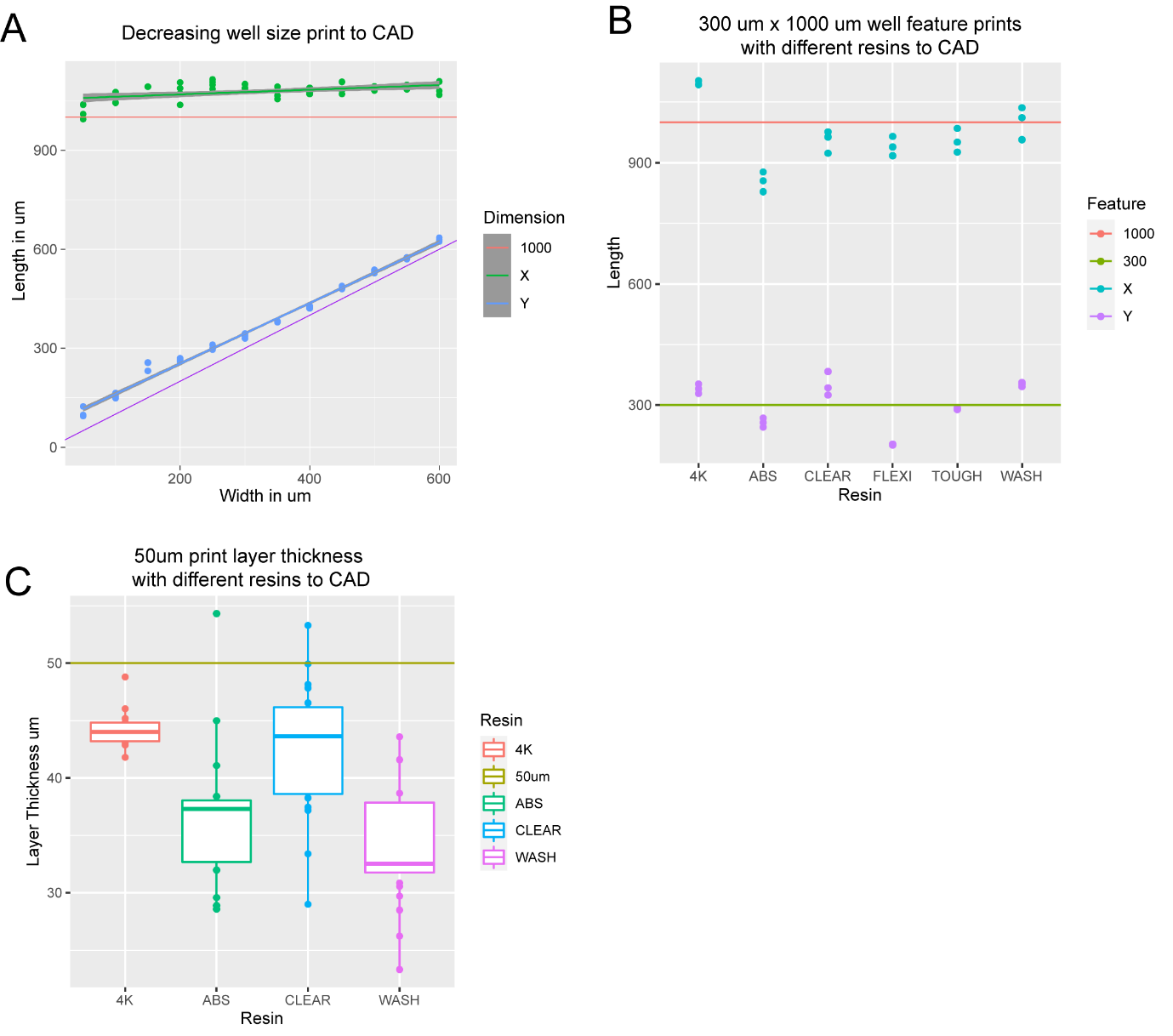


**Figure S11: 3D print dimensions are homogenous**

(A) Graph comparing X and Y dimensions of 3D printed constructs to CAD specifications in a single device with well dimensions ranging from 600 µm x 1000 µm to 50 µm x 1000 µm. (B) Graph comparing X and Y dimensions of 300 µm x 1000 µm features on 3D printed constructs to CAD specifications for 6 commercially available resins printed on 2 3D printers at manufacturer default settings with a 50 µm layer thickness. (C) Graph comparing the actual layer thickness of 3D printed constructs to CAD specifications for 6 commercially available resins printed on 2 SLA 3D printers at manufacturer default settings with a 50 µm layer thickness. Data points for all graphs can be found in S11A-C-Data.
