# Supplementary material for: Low-cost, versatile, and highly reproducible microfabrication pipeline to generate 3D-printed customised cell culture devices with complex designs
Source: PLoS Biol. 2024 Mar 13;22(3):e3002503. doi: 10.1371/journal.pbio.3002503 (PMC10936828; doi:10.1371/journal.pbio.3002503)
Supplement: S12 Fig — (A) Representative SiR-Tubulin images of motor neuron progenitors seeded in microwells ranging from 600 μm × 1,000 μm to 50 μm × 1,000 μm. Difference in colour indicates depth in focal plane where cells do not reach the micropatterned substrate below. (DOCX) [file pbio.3002503.s012.docx]

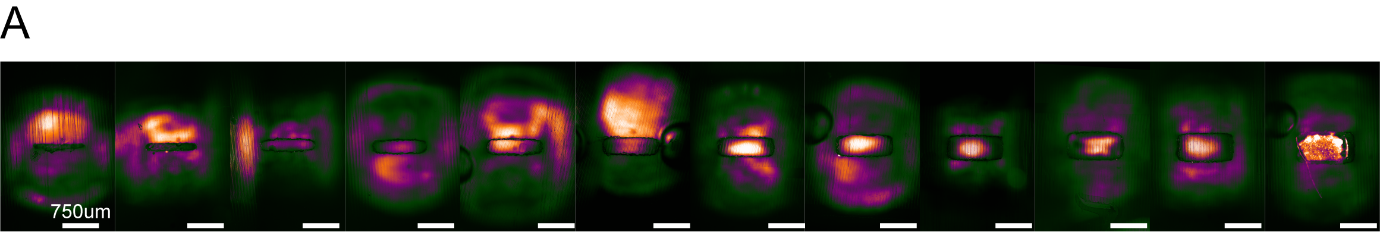


**Figure S12: Non-Plasma devices cannot be used for seeding in microwells**

(A) Representative SiR-Tubulin images of motor neuron progenitors seeded in microwells ranging from 600 µm x 1000 µm to 50 µm x 1000 µm. Difference in colour indicates depth in focal plane where cells do not reach the micropatterned substrate below.
