# Supplementary material for: Low-cost, versatile, and highly reproducible microfabrication pipeline to generate 3D-printed customised cell culture devices with complex designs
Source: PLoS Biol. 2024 Mar 13;22(3):e3002503. doi: 10.1371/journal.pbio.3002503 (PMC10936828; doi:10.1371/journal.pbio.3002503)
Supplement: S13 Fig — (A) Representative SiR tubulin live cell dye fluorescence images of astrocyte and cortical progenitors. Images were captured 2 days following device removal. (B) Channel split of complete circuit after 19 days of culture. GFP transfected motor neurons = green, Glial Fibrillary Acidic Protein (GFAP) identifies astrocytes, β-III–Tubulin identifies cortical neurons and the tubulin in GFP+ motor neurons. Blue device well shapes overlaid for illustrative purposes. (DOCX) [file pbio.3002503.s013.docx]

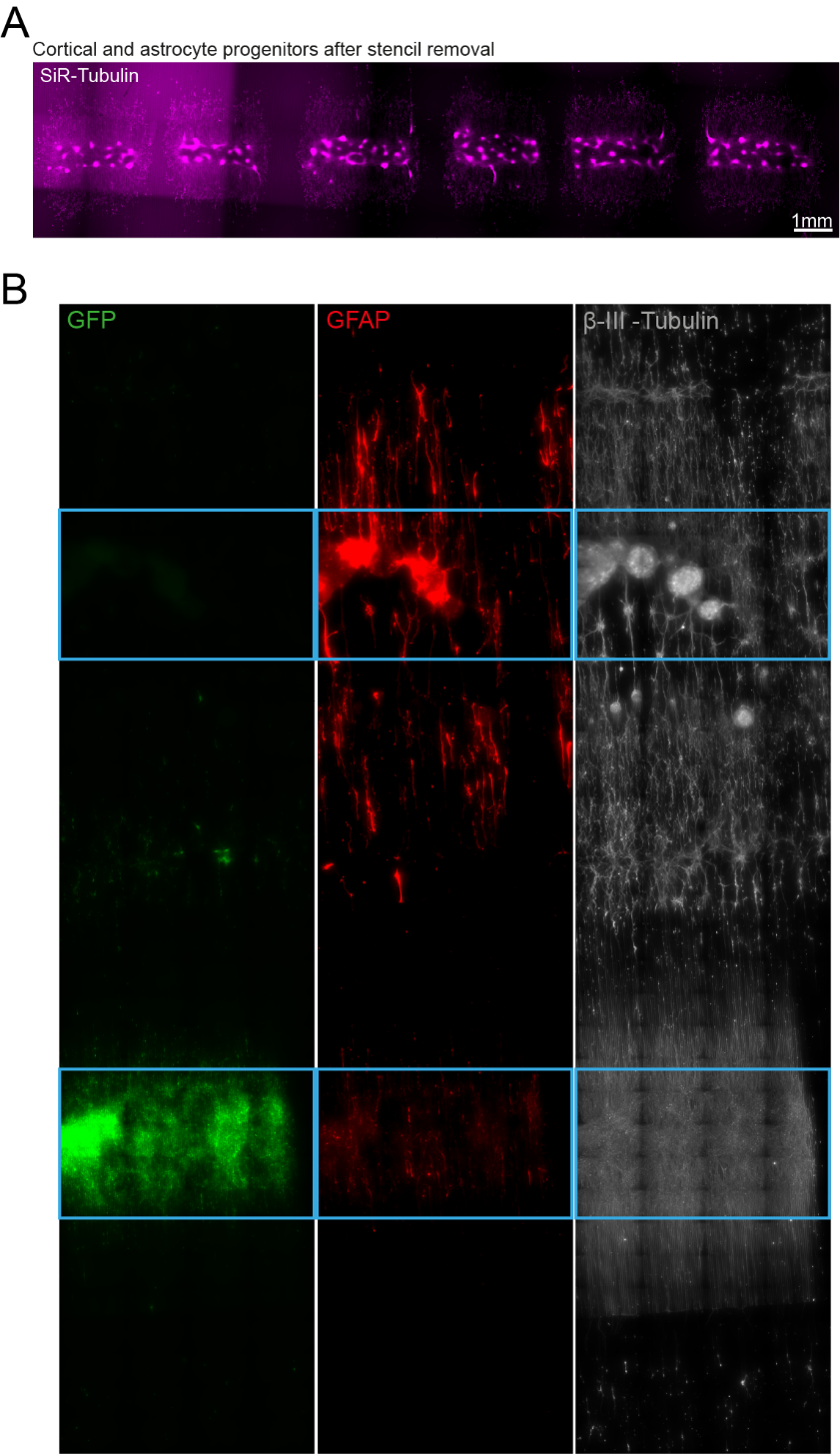


**Figure S13: Spatio-temporal control over cell seeding in an open well**

(A) Representative SiR tubulin live cell dye fluorescence images of astrocyte and cortical progenitors. Images were captured 2 days following device removal (B) Channel split of complete circuit after 19 days of culture. GFP transfected motor neurons = green, Glial Fibrillary Acidic Protein (GFAP) identifies astrocytes, β-III – Tubulin identifies cortical neurons and the tubulin in GFP+ motor neurons. Blue device well shapes overlaid for illustrative purposes.
