# Supplementary material for: Low-cost, versatile, and highly reproducible microfabrication pipeline to generate 3D-printed customised cell culture devices with complex designs
Source: PLoS Biol. 2024 Mar 13;22(3):e3002503. doi: 10.1371/journal.pbio.3002503 (PMC10936828; doi:10.1371/journal.pbio.3002503)
Supplement: S14 Fig — (A) Multiple PDMS casts from 3D-printed devices can be seeded in the same well and seeded with different cell types at different time points. Schematic overview of the multi-device protocol for seeding GFP and non GFP+ motor neurons at different time points in 3 devices in the same well. (B) Representative fluorescence images of cells GFP+ motor neurons seeded in the inner and outer rings of the 3 devices and imaged with devices still on (left) and after seeding of the second non-GFP+ motor neurons in the medial ring after device stripping (right). Representative line profile of imaged cell fluorescence showing segregation of individual populations to their designated rings. (DOCX) [file pbio.3002503.s014.docx]

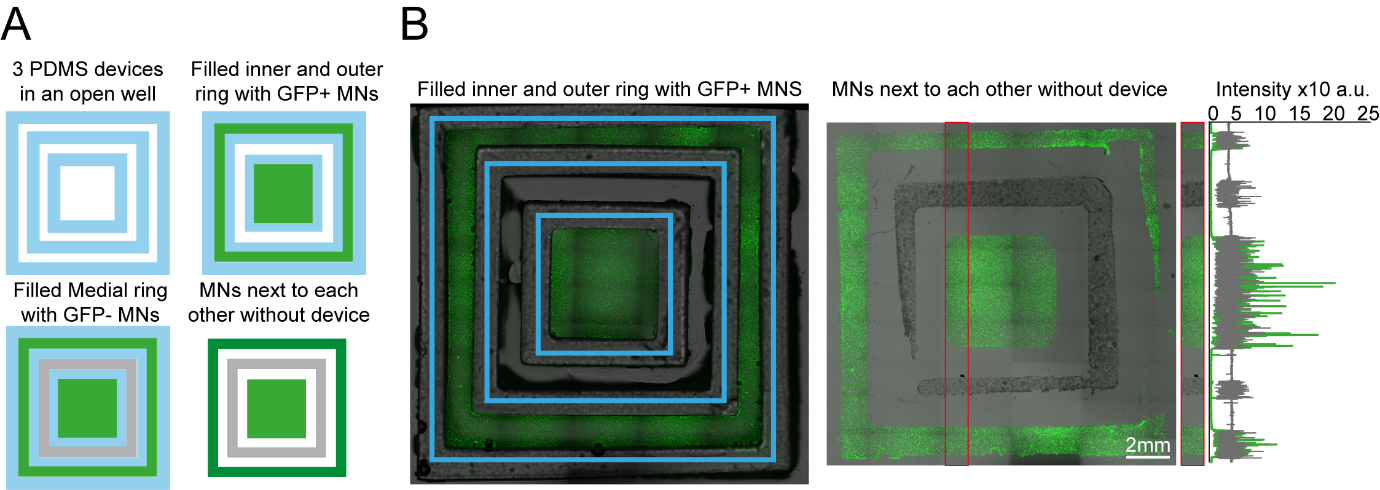


**Figure S14: Plating devices enable manual segregated seeding of different cell types in the same well, device or multiple devices**

(A) Multiple PDMS casts from 3D printed devices can be seeded in the same well and seeded with different cell types at different time points. Schematic overview of the multi-device protocol for seeding GFP and non GFP+ motor neurons at different time points in 3 devices in the same well. (B) Representative fluorescence images of cells GFP+ motor neurons seeded in the inner and outer rings of the 3 devices and imaged with devices still on (left) and after seeding of the second non-GFP+ motor neurons in the medial ring after device stripping (right), Representative line profile of imaged cell fluorescence showing segregation of individual populations to their designated rings
