# Supplementary material for: Low-cost, versatile, and highly reproducible microfabrication pipeline to generate 3D-printed customised cell culture devices with complex designs
Source: PLoS Biol. 2024 Mar 13;22(3):e3002503. doi: 10.1371/journal.pbio.3002503 (PMC10936828; doi:10.1371/journal.pbio.3002503)

**Figure S15: Plating devices enable geometric manipulation of aggregoid cultures in 2D and 3D**

(A) Schematic overview of protocol for manipulating aggregate geometry in combination with existing microgroove and flat substrates. (B) Representative images of SiR-tubulin live-cell-stained motor neuron aggregoids at day 2 (top) and β-III Tubulin staining after 11 days (bottom) of culture. (C) Boxplot of aggregoid aspect ratio fold change by shape between CAD (blue line) and day 2 of culture from β-III-Tubulin channel (top) (D) Boxplot of aggregoid area fold change by shape between CAD (blue line) and day 2 of culture from β-III Tubulin channel. (E) Representative images of SiR-tubulin stained cortical aggregates after seeding and 24h later in different geometry stencil devices, round (left), triangular (middle) and rectangular (right).


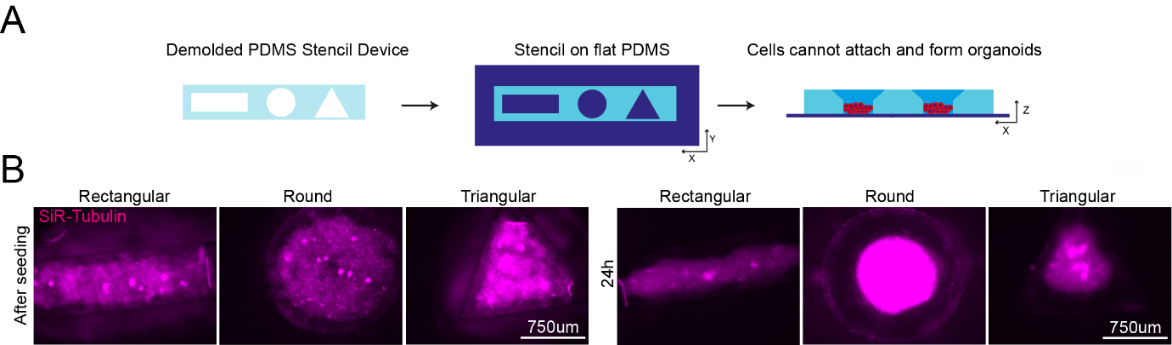

Supplement: S15 Fig — (A) Schematic overview of protocol for manipulating aggregate geometry in combination with existing microgroove and flat substrates. (B) Representative images of SiR-tubulin live-cell-stained motor neuron aggregoids at day 2 (top) and β-III Tubulin staining after 11 days (bottom) of culture. (C) Boxplot of aggregoid aspect ratio fold change by shape between CAD (blue line) and day 2 of culture from β-III-Tubulin channel (top). (D) Boxplot of aggregoid area fold change by shape between CAD (blue line) and day 2 of culture from β-III Tubulin channel. (E) Representative images of SiR-tubulin stained cortical aggregates after seeding and 24 h later in different geometry stencil devices, round (left), triangular (middle), and rectangular (right). (DOCX) [file pbio.3002503.s015.docx]
