# Supplementary material for: Low-cost, versatile, and highly reproducible microfabrication pipeline to generate 3D-printed customised cell culture devices with complex designs
Source: PLoS Biol. 2024 Mar 13;22(3):e3002503. doi: 10.1371/journal.pbio.3002503 (PMC10936828; doi:10.1371/journal.pbio.3002503)
Supplement: S17 Fig — (A) Design of a 3 compartment device. (B) Optical profilometer images with indicated positions of measurement (red circle, blue triangle). (C) Profile of dimensions across the channel (blue line in B) with measured depth and width. (D–F) Time-lapse images of a fluorescent solution spreading through the device. (DOCX) [file pbio.3002503.s017.docx]

**
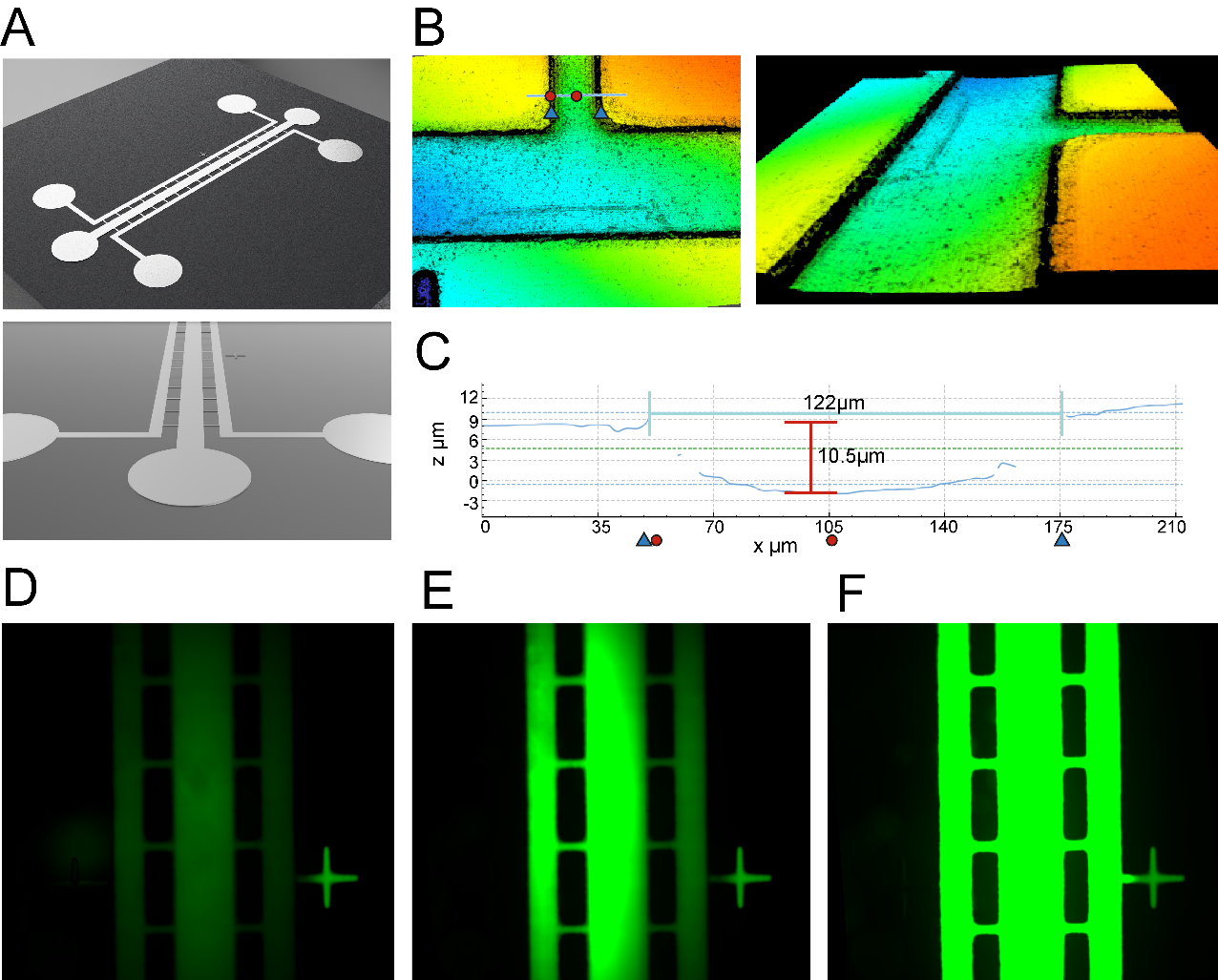
**

**Figure S17: 3D printed mould for microfluidic devices**

(A)Design of a 3 compartment device (B) Optical profilometer images with indicated positions of measurement (red circle, blue triangle) (C) Profile of dimensions across the channel (blue line in B) with measured depth and width (D, E, F) Time lapse images of a fluorescent solution spreading through the device.
