# Supplementary material for: Low-cost, versatile, and highly reproducible microfabrication pipeline to generate 3D-printed customised cell culture devices with complex designs
Source: PLoS Biol. 2024 Mar 13;22(3):e3002503. doi: 10.1371/journal.pbio.3002503 (PMC10936828; doi:10.1371/journal.pbio.3002503)

**Figure S18: Optimisation and protocol for 3D muscle culture using PDMS constructs from 3D printed moulds**

(A) Images show three different mould sizes tested to optimise the volume of the hydrogel mix at a side and bottom view. (Bi-iii) Images showing moulds with (yellow arrows) and without grooves at various perspectives. (Biv) Image shows top view of agarose once mould is removed. (Ci-ii) Images show posts with and without a ring placed underneath the arms from a front and top view. Rings were designed to match the size of one well of a 12-well plate. (Ciii) Images showing posts inserted into agarose moulds at a side view and bottom view from underneath the plate, rings allow posts to be inserted at a specific height.


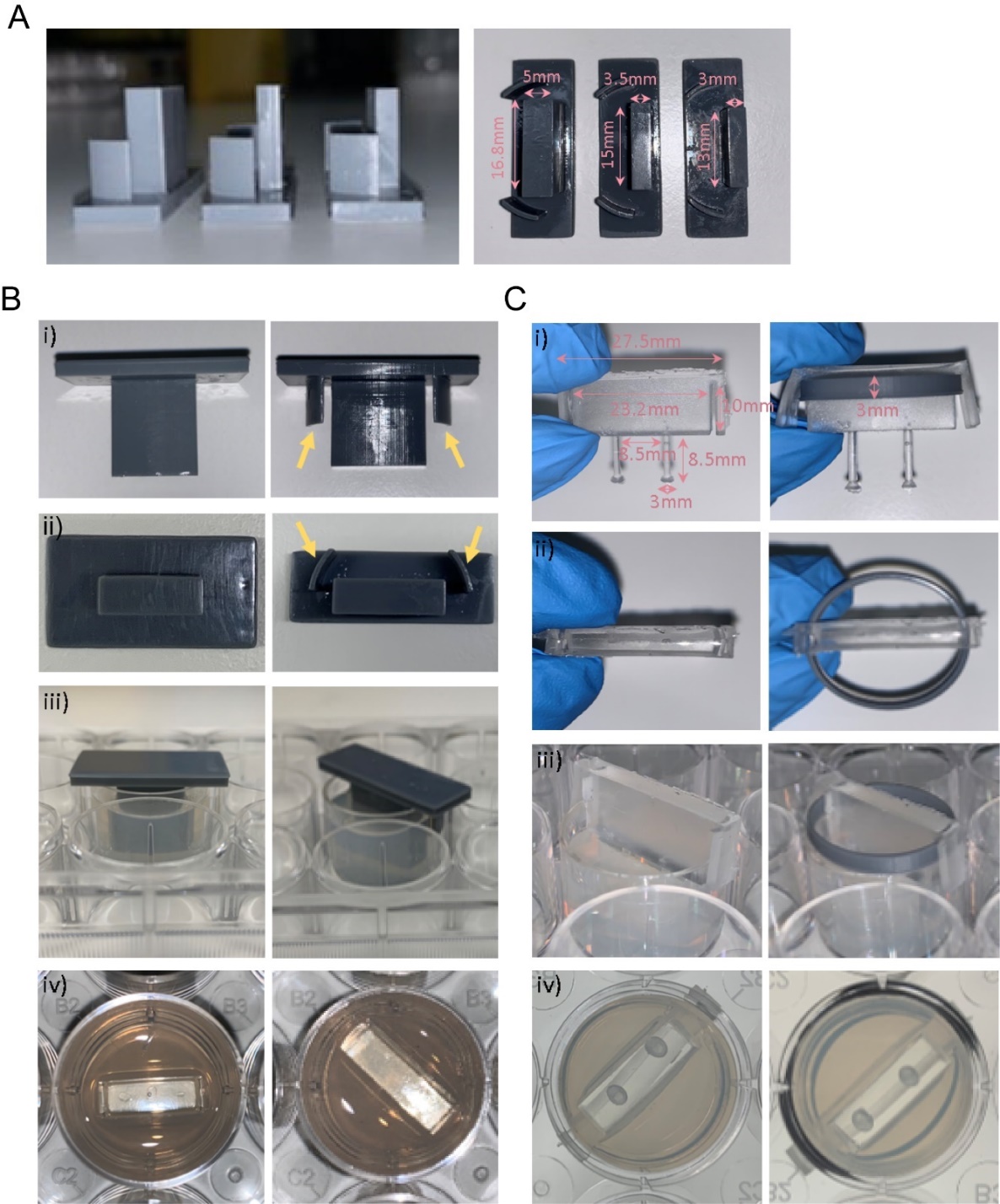

Supplement: S18 Fig — (A) Images show 3 different mould sizes tested to optimise the volume of the hydrogel mix at a side and bottom view. (Bi–iii) Images showing moulds with (yellow arrows) and without grooves at various perspectives. (Biv) Image shows top view of agarose once mould is removed. (Ci–ii) Images show posts with and without a ring placed underneath the arms from a front and top view. Rings were designed to match the size of 1 well of a 12-well plate. (Ciii) Images showing posts inserted into agarose moulds at a side view and bottom view from underneath the plate, rings allow posts to be inserted at a specific height. (DOCX) [file pbio.3002503.s018.docx]
