# Supplementary material for: Low-cost, versatile, and highly reproducible microfabrication pipeline to generate 3D-printed customised cell culture devices with complex designs
Source: PLoS Biol. 2024 Mar 13;22(3):e3002503. doi: 10.1371/journal.pbio.3002503 (PMC10936828; doi:10.1371/journal.pbio.3002503)
Supplement: S1 Text — (DOCX) [file pbio.3002503.s019.docx]

## 1.0 Background

The field of 3D printing and bioengineering has developed rapidly during the last decades, in turn entering and understanding a new field can be challenging. This document will provide more detailed insights to the fundamentals of the techniques and their applications as well as limitations. The main document focusses on the explanation of the developed pipeline, while this document illuminates the surrounding knowledge on 3D printing and bioengineering.

Several microfabrication techniques have been adapted over the years to the needs of the biology research community, and as a result the use of micropatterned substrates, microchamber devices and other engineered substrates has increased exponentially. One of the most versatile combinations of techniques to obtain PDMS devices for biological experiments is photolithography coupled with soft-lithography^1,2^ .Photolithography is based on the deposition of layers of UV-sensitive photoresist of specified thickness, which is then exposed to a UV source with either a photomask or by direct laser writing to create the desired design, before developing the exposed photoresist. This process can be repeated for multiple layers and allows the creation of 2.5D designs (i.e. multiple planar structures of different thickness stacked to form one single set of features) (Diagram1 A, B). While these techniques can be used to create advanced in vitro culture systems with micron-scaled features, photolithography-based pipelines also present some limitations; for example, they can only create a single layer at a time with a given height determined by the photoresist layer's properties and are generally limited in the aspect ratio of the features that they can create. As a result, features are limited to 2.5D designs with defined thickness, lacking 3D volumes, curves, or interconnected shapes (Diagram 1B). Moreover, generating multi-layer constructs for complex features involves multiple photolithographic steps, which can be time-consuming, prone to errors, and costly (Diagram 1 A-B). While these limitations can be obviated by recent improvements and optimisations in photolithography, such as grey scale photoresist^3,4^ and high resolution 2-photon based lithographs^5,6^, the result is often increased complexity in the fabrication process, and in costs or availability of the necessary instruments. This creates the need for a technique that is cost effective and provides features ranging from um to cm scale. Advancements in 3D printing, in price and accessibility, render them as a great tool for biology labs. Here we outline the fundamental and commercially available printer types, highlighting their advantages, disadvantages and estimated resolution. The printing process overall always follow the same pattern though, with an idea, the design, the conversion to a 3D printer suitable file format and the actual print. Later we go into detail, which software to use for the design and the conversion to the correct file format, in this case tailored to the Phrozen 4k Mini.

Fused deposition modelling (FDM) allows to take a solid polymer, heat it to its melting point and extrude it onto a printbed. The printer has a heat controllable hotend through which the filament is pressed, and a coordinate system to move the hotend to a desired position in x, y and z. To construct an object, the design is converted into g-code, giving the coordinates for the printer to move to, while extruding the polymer. This type of printing takes rather long as every position is passed through and the object is constructed line by line. However, it allows to use a vast range of different polymers with specific properties such as chemical stability and durability. The resolution of this printer is determined by the filament size, which can range from 1,75mm to 3mm, and the layer height, which can as low as 50- 100um but this ultimately depends on the printer model.

Vat polymerisation is a specific type of 3D printing that generates constructs in a layer-by-layer fashion by forming features onto a build plate using UV-curable resin. There are 3 main subtypes of vat polymerisation-based 3D printers, defined by their UV light source, which dictate resin choice (due to light wavelength – extensively reviewed^7^, as SLA printers usually use ~395nm light and LCD screens emit longer wavelength of ~405nm), part resolution, printing time, and printer cost. The original illumination technique for vat-polymerisation-based printing known as Stereolithography (SLA) utilised lasers, which function by scanning across designs on the build plate pixel-by-pixel to polymerise resin. These systems, such as the Formlabs 3B, offer sub-millimetre resolution (25µm X Y, 25 µm Z) and multiple proprietary optimised resin/curing options at the expense of printing speed, systems size, and cost, tending to be designed primarily for business use^8^.

The most common and cost-effective printer light sources are LCD screens, these illuminate each full layer of the design all at once, with X Y resolution dictated by the resolution of pixels within the screen, and Z resolution by the precision of the printer build plate motor. These printers offer similar resolution (35 µm X Y, 10 µm Z) and, with the advent of mono-colour LCD screen technology, higher printing speeds than laser-based systems at a lower cost-of-entry and running^9^.

Digital Light Processing (DLP) printers employ a similar full-layer polymerisation method to LCD printers except designs are first projected onto a digital micromirror device (DMD) before being reflected onto the build plate. The benefit of DLP over LCD light sources is that the intensity of the illumination is uniform across the build area, meaning that µm-scale features are easier to manufacture due to a lack of pixel-pixel shadowing that can be present for LCD screens. However, DLP printers are more costly than LCD systems for equivalent resolution, and their resolution changes across print scales due to DMD manipulation of a single light source (51 µm X Y, 10 µm Z) for USD 407 Anycubic Photon D2^10^.

**
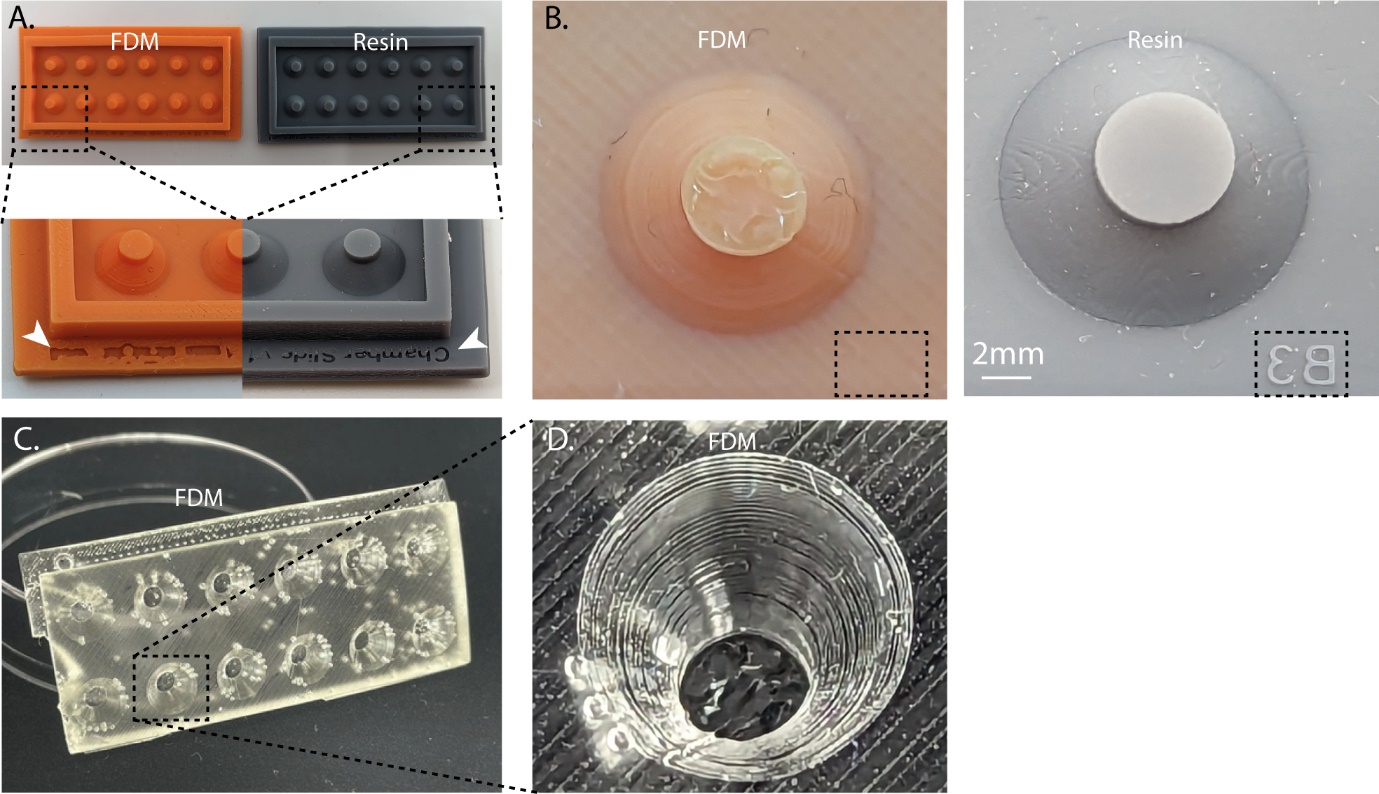
**Overall, driven by recent advancements of resolution and financial accessibility vat polymerisation 3D printers show the potential to bridge the gap between μm-resolution photolithography and mm-resolution fused deposition modelling (FDM) 3D printers (Diagram 1), whilst remaining economically accessible to any lab. Because of the positive characteristics UV vat polymerisation printers provide while remaining accessible, coupled with the rapid and easy fabrication of complex shapes in 3D, UV resin vat polymerisation represents in theory an ideal technique to create bespoke culture vessels, inserts and other devices to increase complexity within biological experiments without sacrificing control over culture conditions ^11,12^.

**Comparison of feature quality between FDM and Vat polymerized samples**

(A) Chamber devices used in Figure 5 were manufactured using a FDM (Bambu Labs, 0,4mm nozzle with 0.16mm settings) printer and a 3D UV-resin printer (Phrozen mini 4k, 50um settings). Zoom in images show a clear constructed writing for the UV resin printer, lacking for the FDM sample. (B) Representative images highlight the flat surface of resin printed samples, especially on the flat top of the pillar. The dotted box highlights the absence of the well labelling in FMD prints, and a clear label in the resin sample. (C-D) PDMS cast of the FDM mould, clearly highlights the large print and build lines of the sample.

While using PDMS as a material it is to note that, PDMS is a porous material that absorbs small molecules and growth factors, necessitating single usage of PDMS constructs and frequent preparation of new devices. For this reason, fast and reliable manufacturing of these devices is often a crucial limit step in several experimental pipelines.

**Diagram 1: Description of 3D vat polymerization and soft lithography and scale comparison across methodologies**

(A) Schematic overview of the UV resin vat polymerisation process and post-processing with a time estimate for production. Printing times range depend on the printed volume but for cell culture device range from 10min to 1h. (B) Schematic overview of photolithography and an estimated time to completion of a print. Print time is an estimate for feature creation across the whole silicon wafer and alters in between prints, depending on the design. Overall, UV resin vat polymerisation is faster compared to photolithography, as it uses whole field illumination and no single point illumination as photolithography. (C) Scale comparison of 3D printing methods UV resin vat polymerisation, FDM and photolithography. Comparison of maximal resolution achieved with UV resin vat polymerisation, with current printers, and photolithography. (D) Representation of feature designs achievable with UV resin vat polymerisation and photolithography. UV resin vat polymerisation offers a wide range of feature dimensions and complexity compared to photolithography which underlies technical limitations for designs.


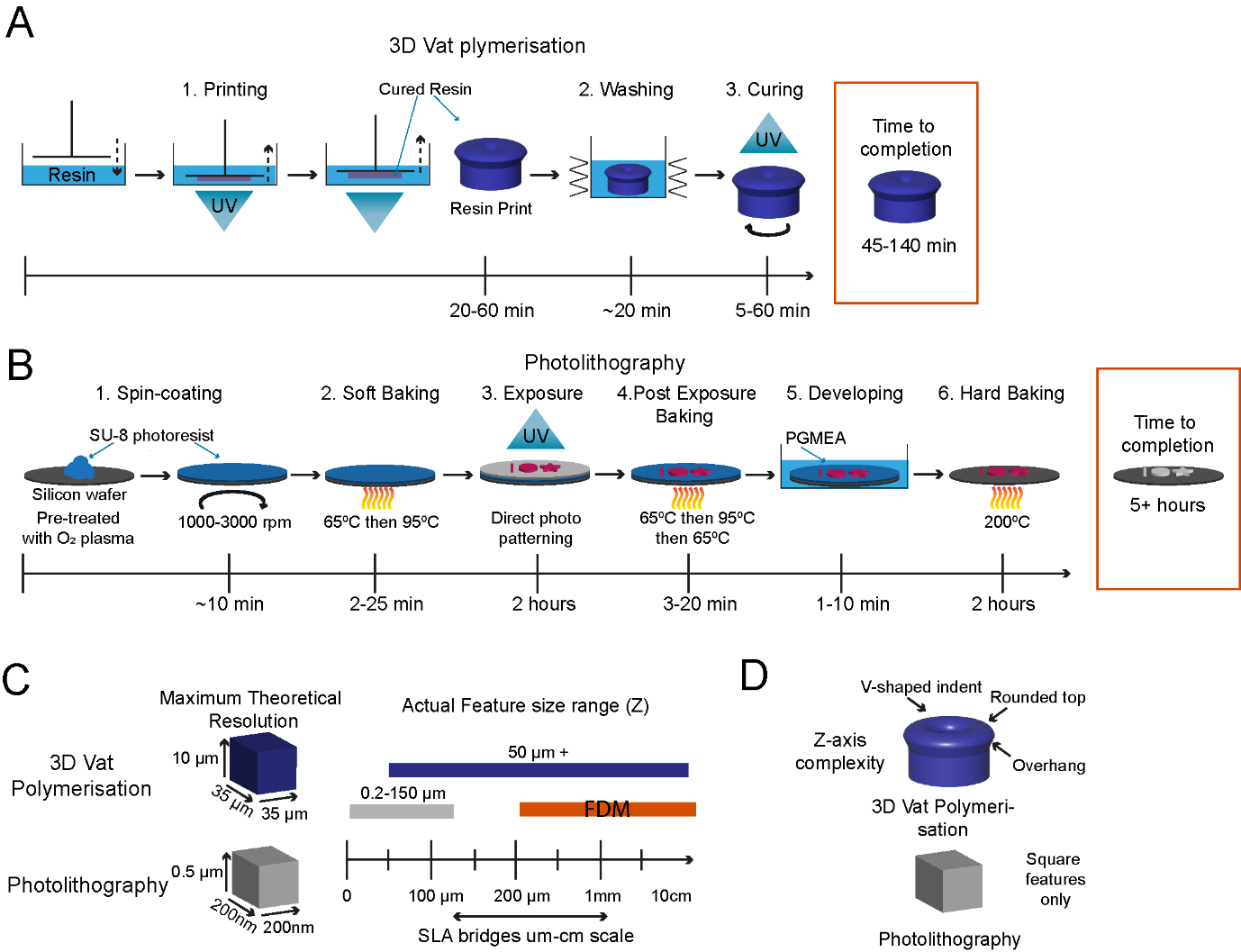


## 2.0 Design

When designing a part, a clear idea of the final construct and its function are important to make the process quick and easy. For example, visualising the spatial relationships between a 3D printed mould and the PDMS cast it is intended to form in the printing – can you print the features you need? Casting – does the resin you plan to print with enable PDMS curing with/without enamel coating? Demoulding – does the part have multiple/complex components? Can it be demoulded in a single step? and Cell culture function – is it a stencil device? Does it need to be thin for imaging? all dictate the design strategy at the CAD level.

**Software**

There are several CAD software’s online, both subscription based and open source, the most common are [Fusion360](https://www.autodesk.co.uk/campaigns/education/fusion-360) (subscription based) and [Tinkercad](https://www.tinkercad.com/) (open source). Although both are offered by Autodesk Inc. Tinkercad is a high-level design software, useful for creating designs rapidly from a menu of shapes. Whilst Fusion360 is a more powerful software which builds designs from fundamental sketch components, enabling the creation of more complex and adaptable designs. Although Fusion360 is subscription based, for those in research institutions with a yearly renewable education license can be obtained so it is beneficial to opt for Fusion360 over tinkercad in most cases. In addition, there is a wealth of design tutorials on YouTube to help get to grips with Fusion360.

**Picking a resin and printer (SLA)**

Depending on the intended function of the printed part, resin and printer selection is vital to the success of the construct. For example, ABS like resin has good mechanical properties but poor compatibility with PDMS casting. Prints with small features <200um and curved surfaces should be printed with high-resolution resin to improve fine feature formation and curved surface smoothness. Additionally, it is important to consider the size and number of parts to be fabricated as printers have different sizes and shapes of build plate. A breakdown of the capabilities of the resins used in the Serio lab and printers they are suitable for is below.

| Resin | Layer thickness settings | Type of prints | Currently printed on |
| --- | --- | --- | --- |
| 4K aqua Gray | 25um, 50um | High res, curved | 4K, Mars 3 |
| Elegoo ABS like | 50um (from online repository) | Strong prints, non PDMS cast | 4K, Mars 3, Photon S |
| Next Dent Ortho Clear | 50um (default) | Biocompatible/non leaching – non PDMS cast | 4K |
| Flexible X | 50um (non-optimised) | Flexible prints/ non PDMS casting | 4K |
| Premium tough | 50um (non-optimised) | Parts with good mechanical properties, non PDMS cast | 4K |

**Designing parts for PDMS casting**

As mentioned, the design of parts intended for PDMS casting requires a clear plan of the final function of the part. Here, it is important to understand the limits of printing resolution, PDMS mechanical properties, and practical aspects of the casting process such as using a positive mould to fabricate a negative with PDMS.

For example, whilst parts with features of 100 µm in XYZ and spacing can be manufactured, moulds for microgrooves of 100um width, depth and spacing require tolerances of 50um to be added to the spacing of grooves. This accounts for bleaching that occurs between the channels as resin drainage is worse for these types of features than for standalone features. Less spacing than this between channels results in shallower features than specified at this depth. Another factor to note is that when printing micro features (<200um) decreasing layer thickness does not guarantee improved resolution. For squared/single profile features, having less layers by using 50um layer thickness can give better results than with 25um layer thickness for the same print due to potential overcuring of the smaller layers. Lower layer thicknesses are helpful for prints with features that have more complex shapes e.g., round/v shaped microgrooves as smaller layers smooth gradients, providing a more accurate replication of the CAD with impacts on cell behaviour when plated in constructs.

**Minimum feature sizes optimised:**

4K resin on 4K/Mars 3 printer @25um layer

| Shape | CAD Dimension (um) | Actual Dimension (um) XYZ |
| --- | --- | --- |
| 3 Dimension (feature) | 200x200x200, 200 spacing | 230x220x240, 150 spacing |
| 2 Dimension (grooves) | 100x250, 200 spacing | 120x >100x 240, 190 spacing |

Additionally, both printing and demoulding tolerances should be accounted for when designing a part for PDMS casting. As a rule, an outer wall thickness of 2mm is the minimum suggested to enable successful printing of larger (taller >5mm) moulds and to prevent mould destruction during cast demoulding. For internal design spacing, at >1mm between any features and the walls is recommended (more if you can to help prevent damaging casts when demoulding), with >500um between features >500um in height. Aspect ratios are important here so take this into consideration when designing too. See diagram below.


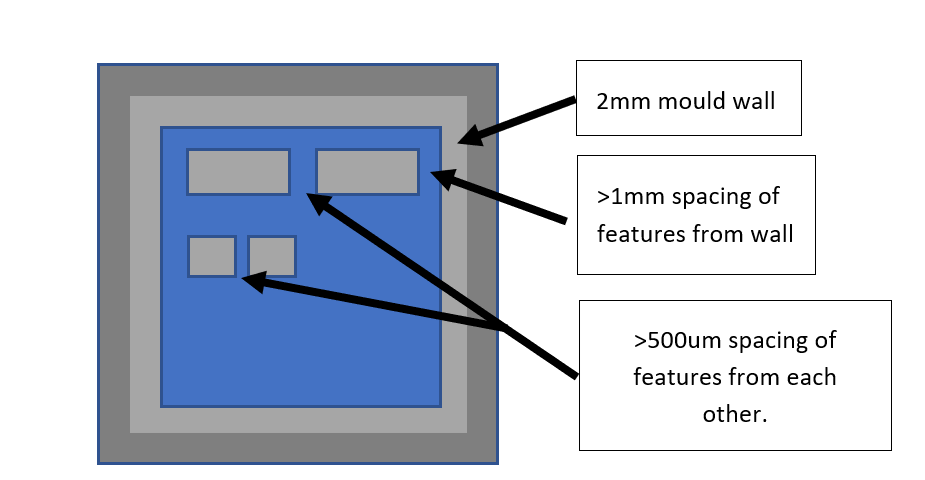


**Designing parts in Fusion360**

In fusion 360, all designs are generated from parametrically constrained **sketch** objects. This ensures that designs can be easily modified by altering any parameters in the sketch, without having to restart the design process. Sketches are formed into 3D objects through **extrusion**. Modification of 3D object can then be performed with several operations including: **filet, chamfer,** and **shell** to generate a final part. Below is a short example of how to construct a stencil mould design as demonstrated in the SOL3D manuscript.

**Stencil Device Tutorial**

Select **Sketch** from the ‘solid’ menu, click on a plane, and create the base of the stencil device mould by clicking again to create the sketch, typing in the desired length of each dimension. Here, lengths of 40mm X and 30mm Y were chosen. Once happy, click ‘Finish Sketch’ on the right-hand tool bar to complete the sketch.


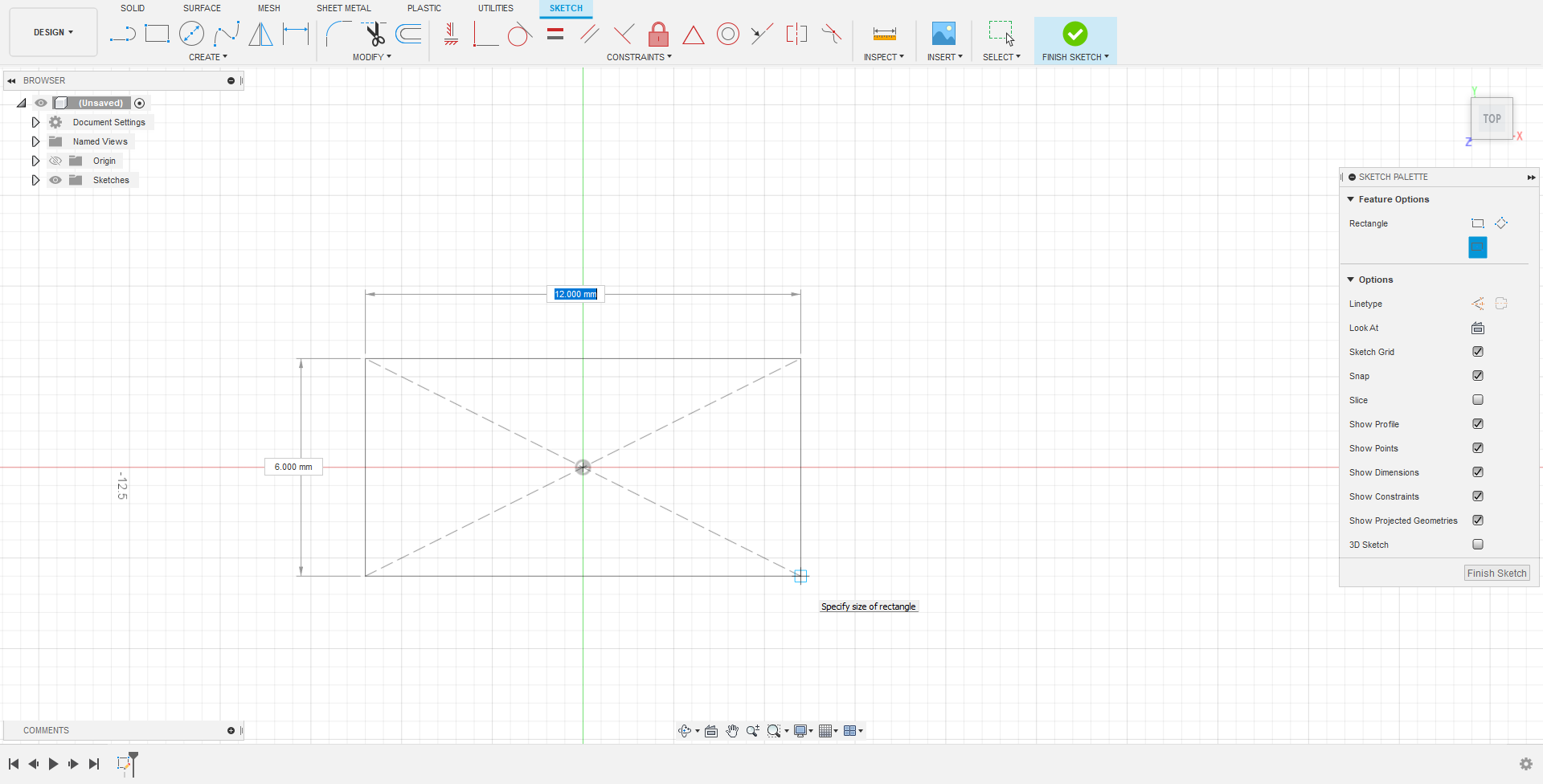


Once the sketch is complete you can **extrude** the shape into a 3D base object by right clicking on the completed sketch and selecting **extrude** from the menu. The Sketch will become darker, and another definable dimension icon will appear on the sketch. Here the sketch was extruded 1mm.


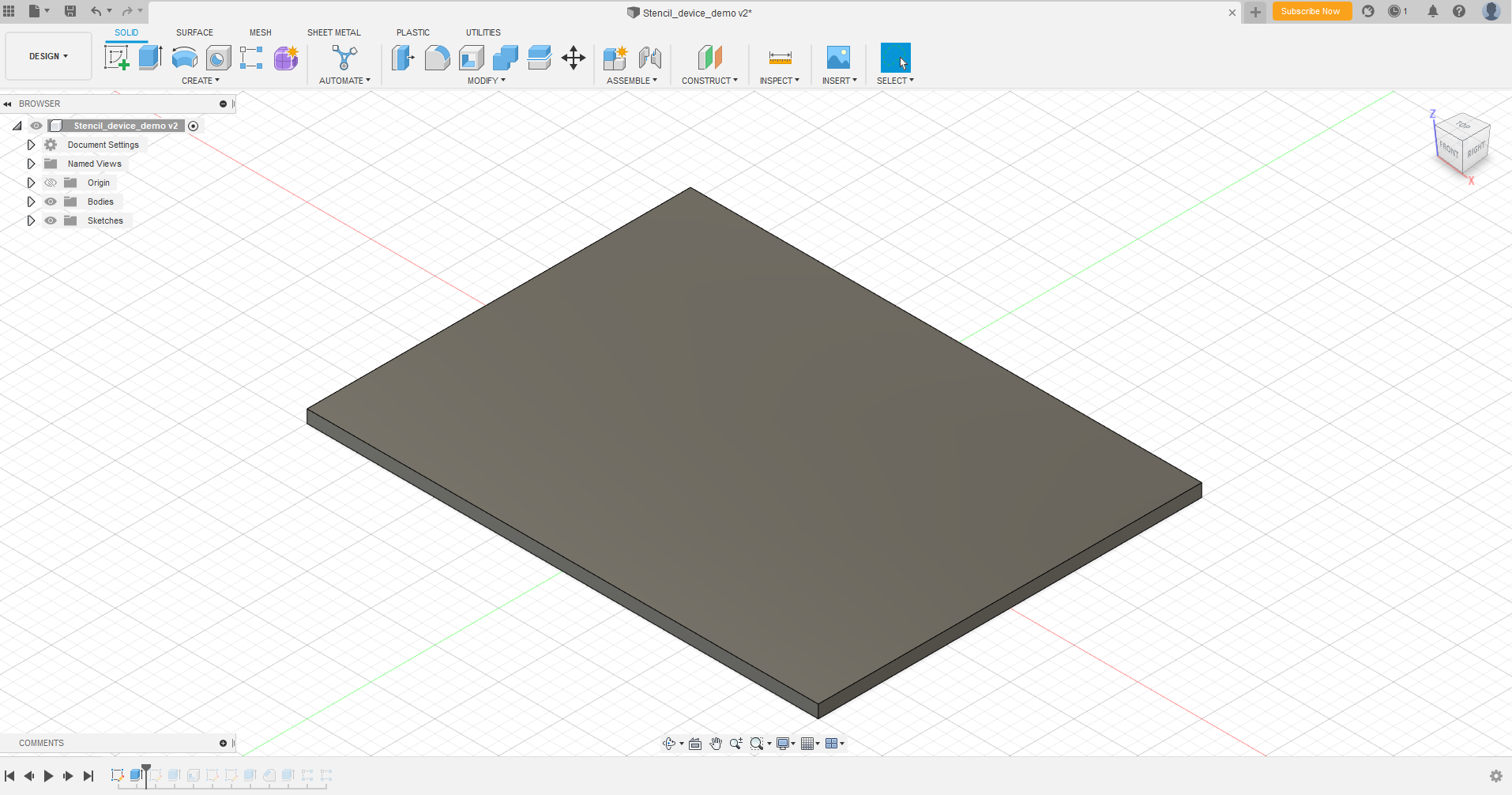


After completing the base section, repeat the **sketch** and **extrude** functions to create the 3D structure that will become the mould for the PDMS casts (below). All sketches so far were made with the ‘centre rectangle’ tool that automatically snaps to the centre of the sketch plane, simplifying the design process. Keep in mind the final dimensions of the cast you want to make here as they will be based on the size of the hole in this extrusion NOT the extrusion itself.
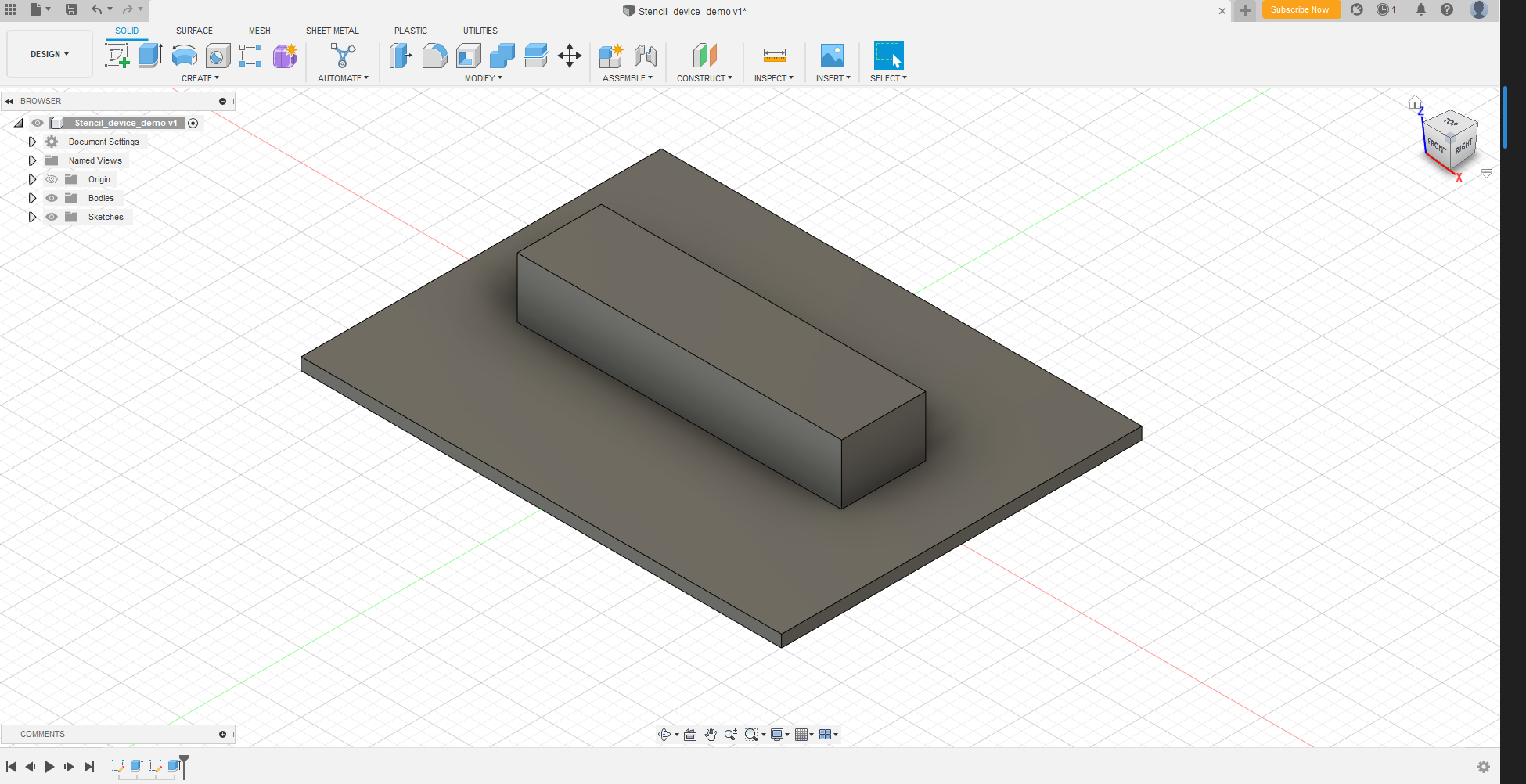


The dimensions chosen here (above) were 27mm X and 7mm Y and 4mm Z to account for losses when using the **shell** tool on the upper face of the extrusion. 1mm shell (below) leaves a 1mm wall on each side, removing 2mm from both X and Y dimensions, leaving a hole 25 mm X 5 mm Y and 4 mm Z.


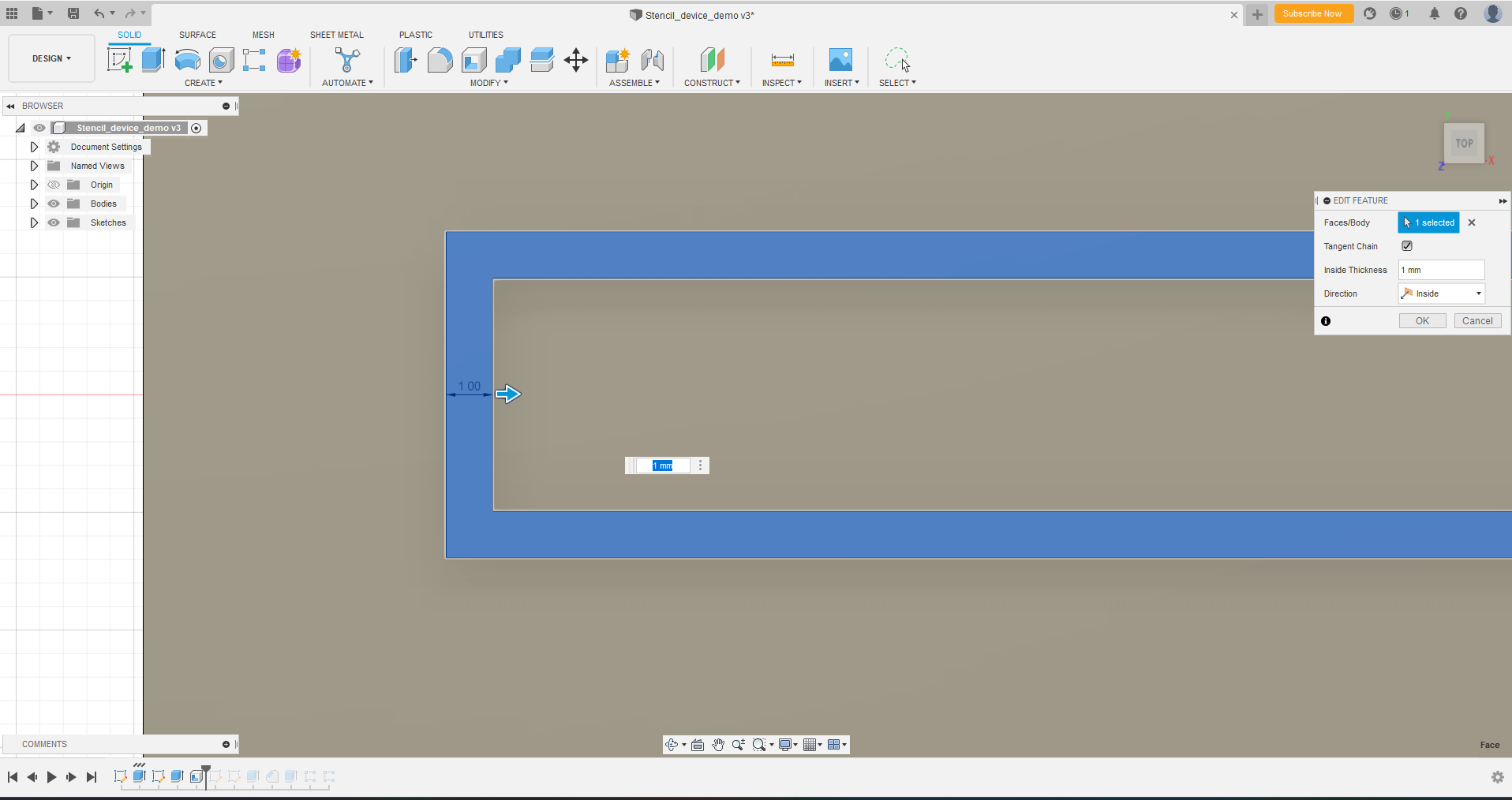


Now the basic mould is complete you can customize the wells you would like to print. In this example we will design a mould to cast a stencil with 4 equally sized 3.5 mm X 1 mm Y 2mm Z wells, with 3.5 mm X 3 mm Y 2 mm Z funnels above, spaced 2 mm apart, with a tolerance of 2.5 mm on each side for easy demoulding. The benefit of SLA printing here is that you can customize well size, shape, and configuration to experimental needs, and editing the Fusion360 design file is quick and easy through rolling back the **history marker (red)**.

To begin the design of the wells, start from the top (the funnel). First, create a sketch as before of the top side of the funnel (3.5 mm X, 3 mm Y). Since the base was generated using a ‘centre rectangle’ sketch the base centreline forms an easy reference point to align sketches too (the tool will also snap to this point to assist you). Here I also sketched a **construction line** (2.5mm from the wall), selected from the ‘sketch palette’ on the right of the screen, which does not form part of the final design but can help in aligning sketches on a design.


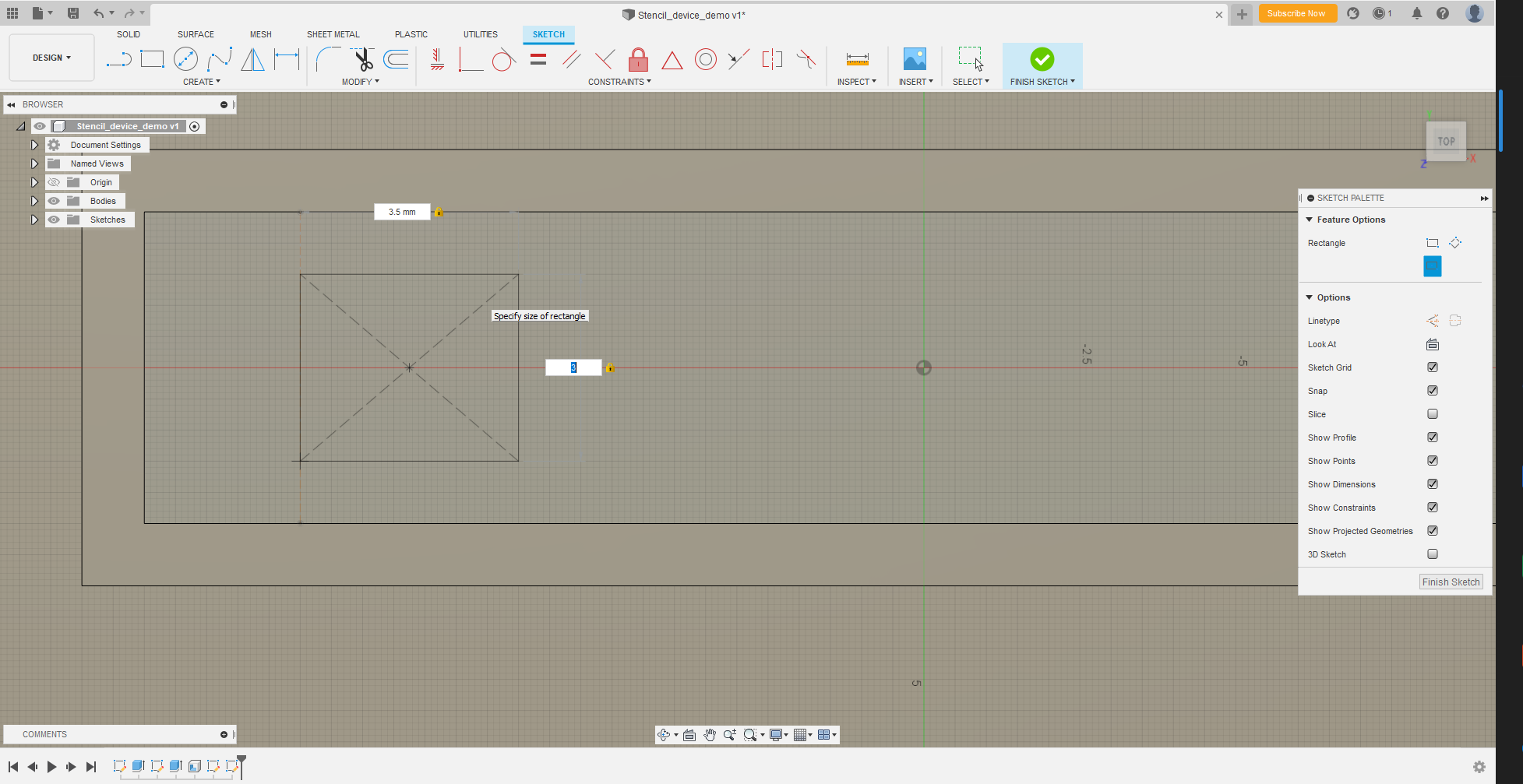


After creating the funnel sketch you can **extrude** as before to the 2mm Z length specified in our design requirements. To form the tapered funnel shape, use the **chamfer** tool by selecting the two long edges (3.5mm) at the top of the extrusion and inputting 1 mm as the distance. This will constrain the well size to 1 mm in Y, the desired dimension. Next, set the angle of the **chamfer** by selecting ‘distance and angle’ in the option menu. This will vary depending on the depth of the well, and the size of the funnel and well. In this case an angle of 62° was used to maximise the funnel size with continuous angle (Below). Fusion360 will not let you input an angle that is too high as it over-constrains the object.


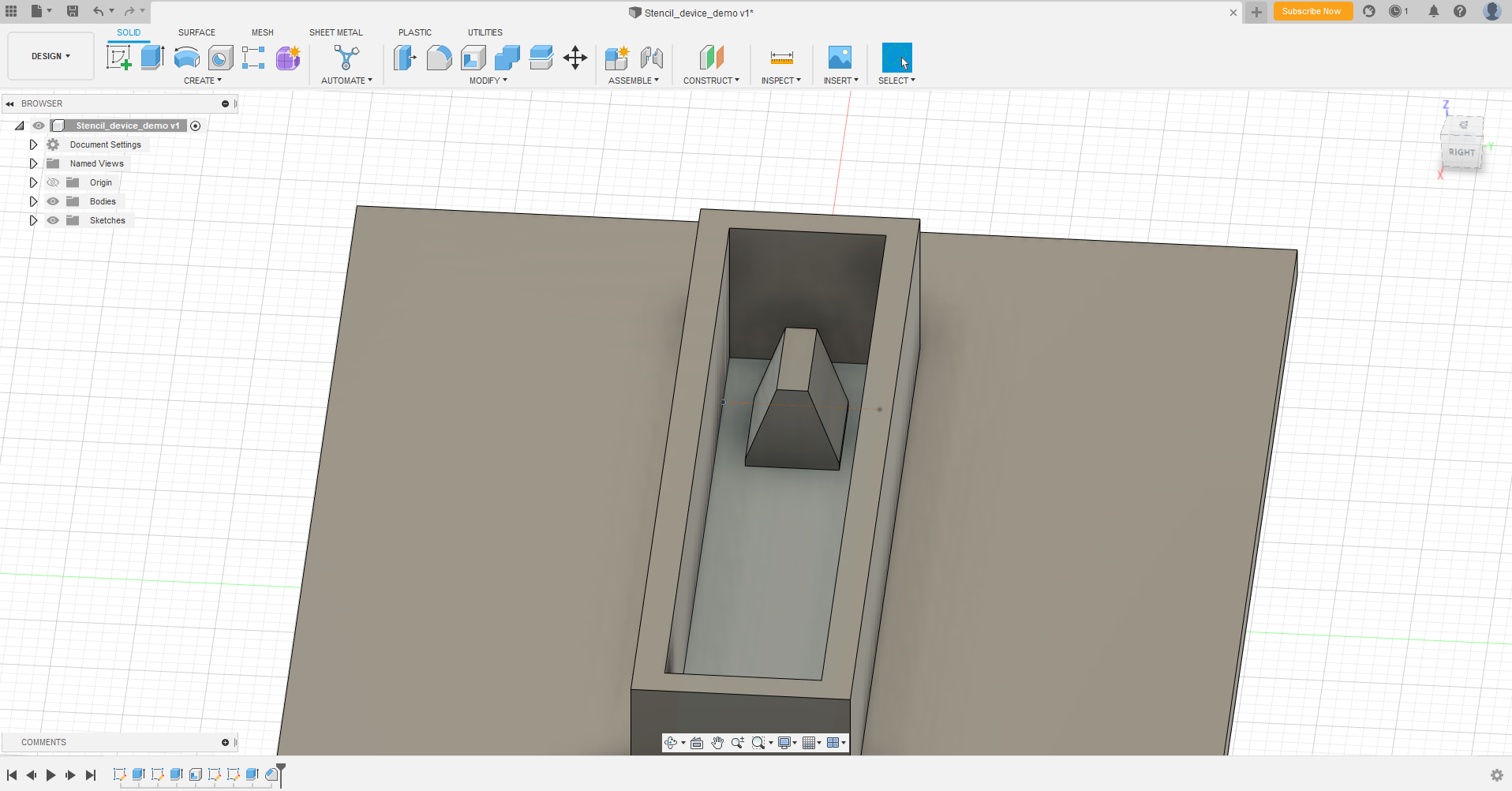


After finishing the **chamfer** for the funnel, simply **extrude** the well 2 mm to match the height of the mould walls (Below). All there is to complete is copying the well within the mould, and copying the whole mould across the base.


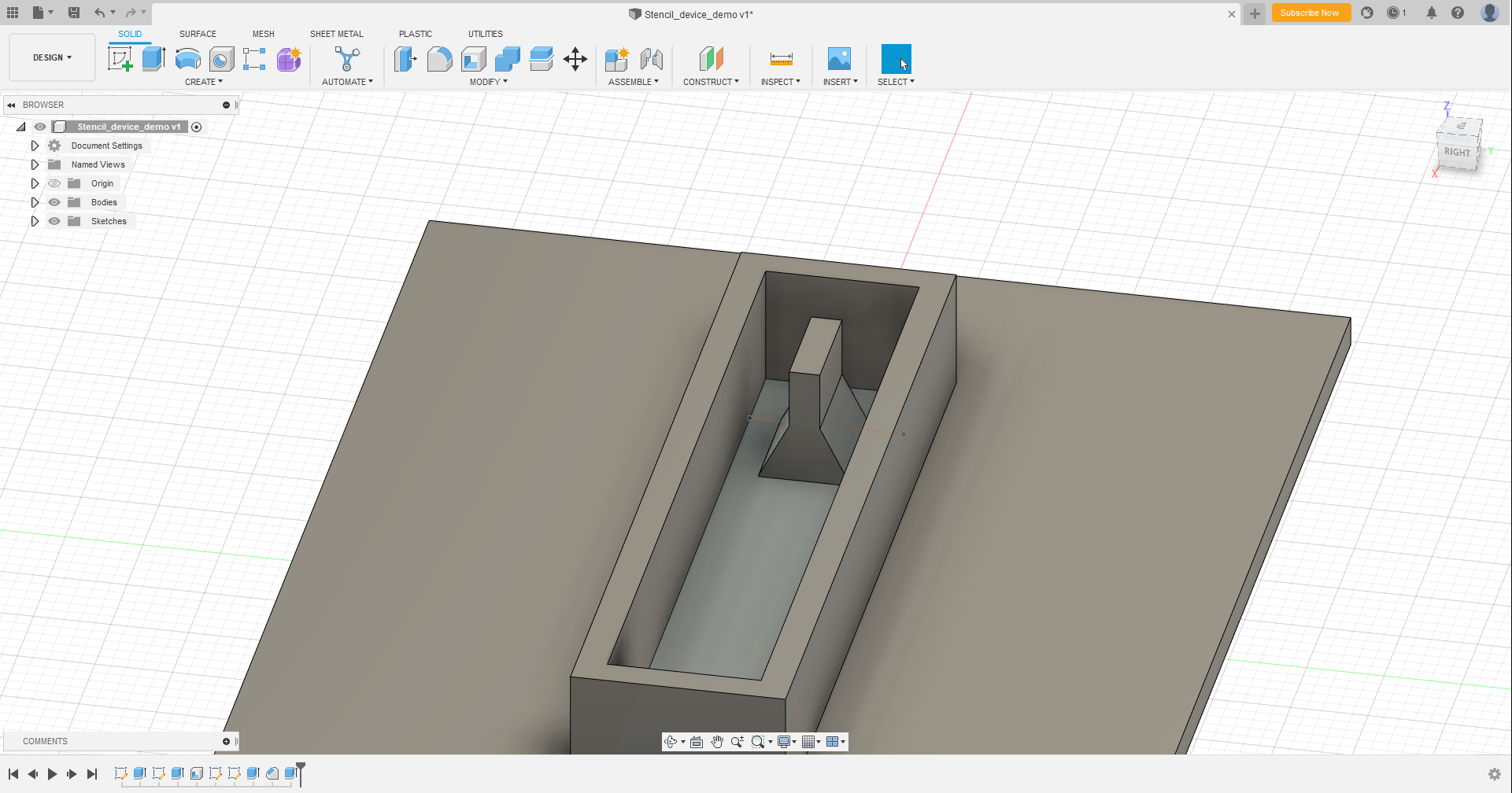


To copy parts of your designs, use the **rectangular pattern** tool (below). In the options panel on the right be sure to select ‘faces’, before selecting all the faces you want to pattern. Here you can see there are 9 faces selected, this is higher than you might expect because you need to select the small faces at the bottom of the funnel **chamfer**. This is a caveat of the **chamfer** tool where you are unable to create a flush surface as doing this over-constrains the face. Once all the faces are selected you change from ‘objects’ to ‘directions in the side menu and select on the design an edge (selected in blue below) that is parallel to the desired pattern direction. From here you set the number of wells (4) and the spacing between them (5.5 mm) to give a 2 mm gap between adjacent wells and a 2.5 mm gap to the mould wall. Spacing is set from the starting point of the selected objects in the desired direction, which in this case includes the 3.5 mm X dimension of the well. This is important to take into consideration when designing different well sizes.


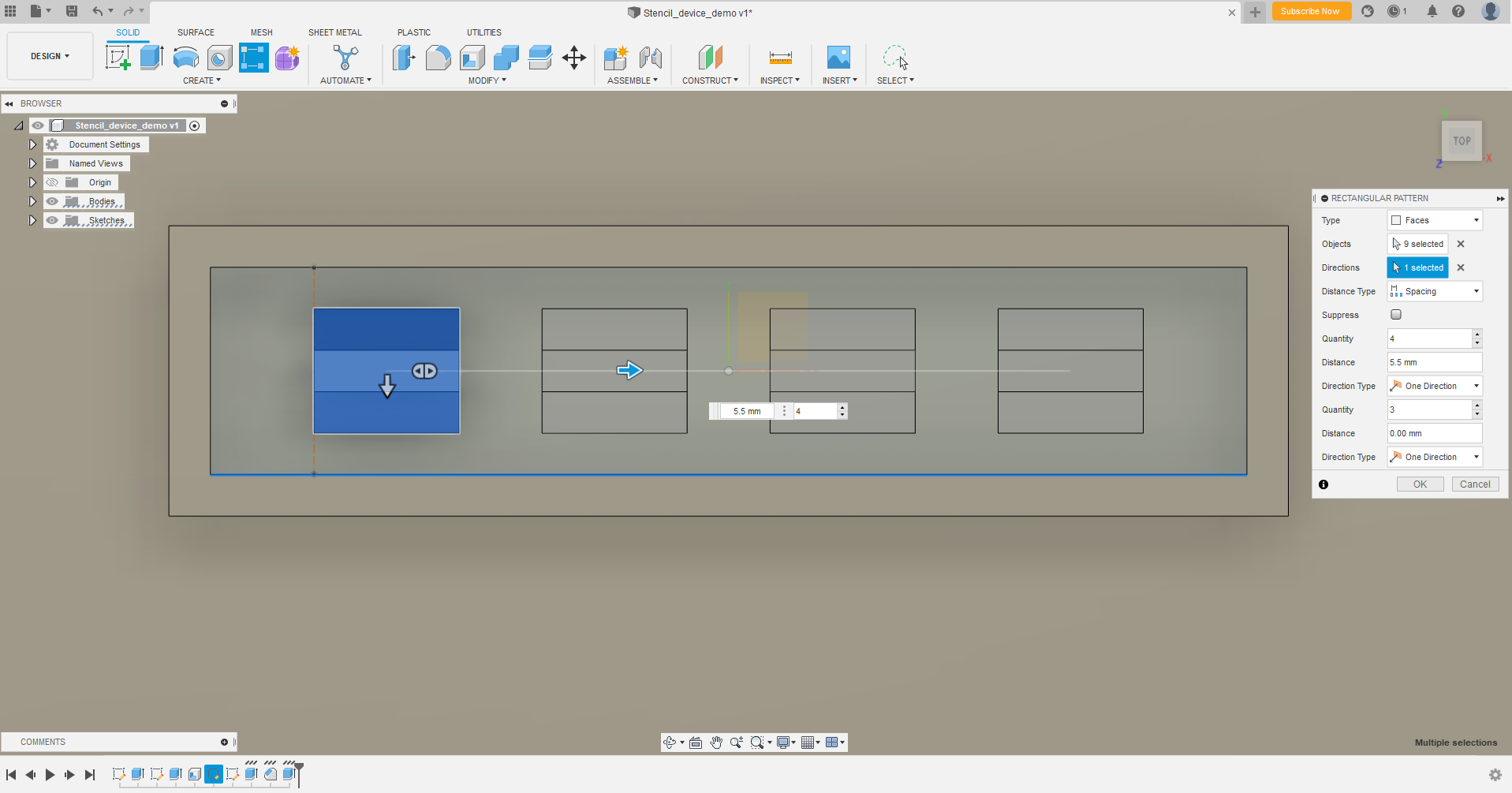


See below the completed single cast mould after using the **rectangular pattern** tool to copy the well within the mould.


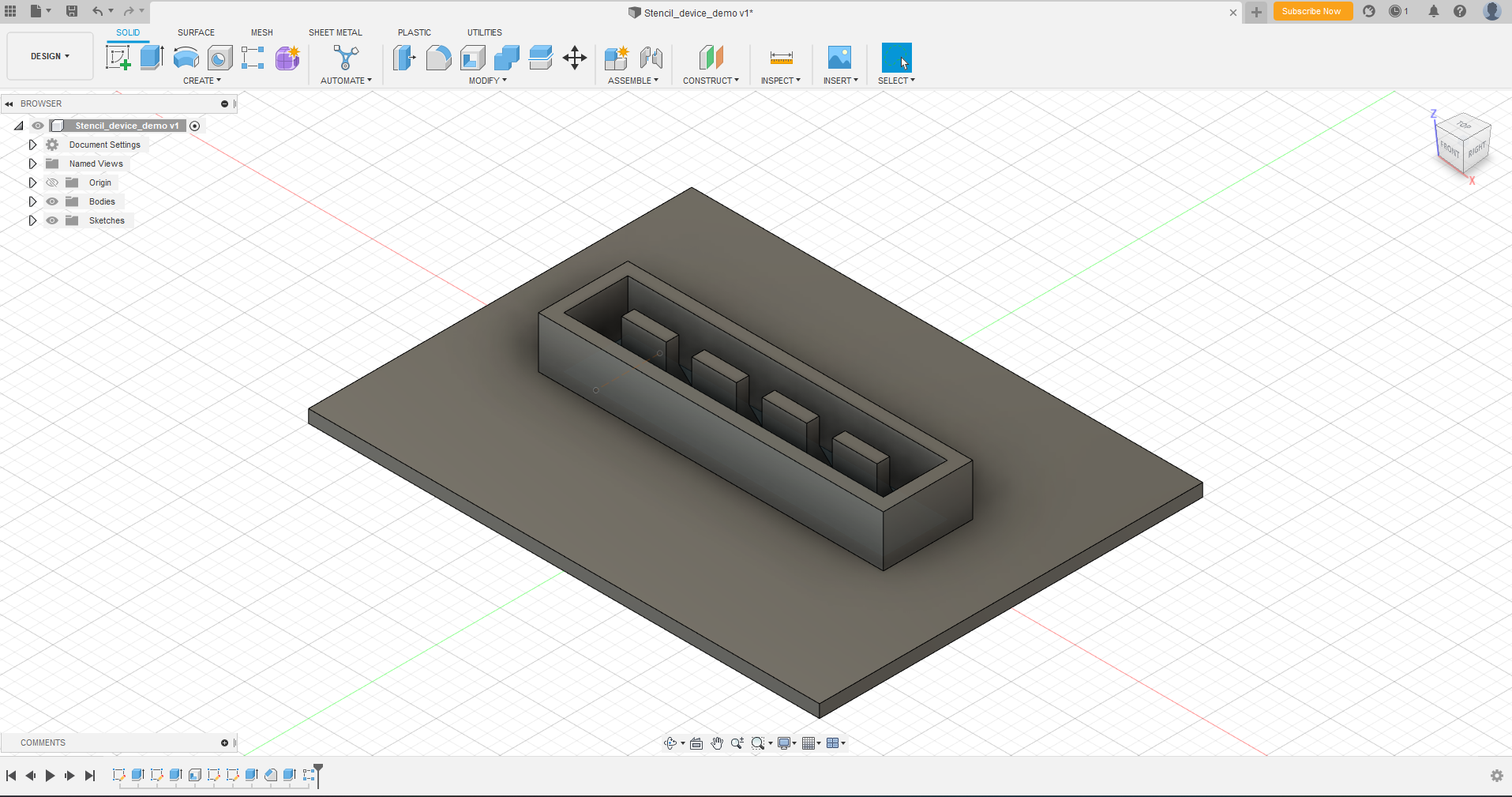


To complete the design, use the **rectangular pattern** tool again to copy the mould you just completed across the full area of the base. The difference between this patterning and the previous is that the mould is copied both above and below its original position, requiring the selection of **Symmetric** from the ‘direction type’ option in the menu panel on the right (below).


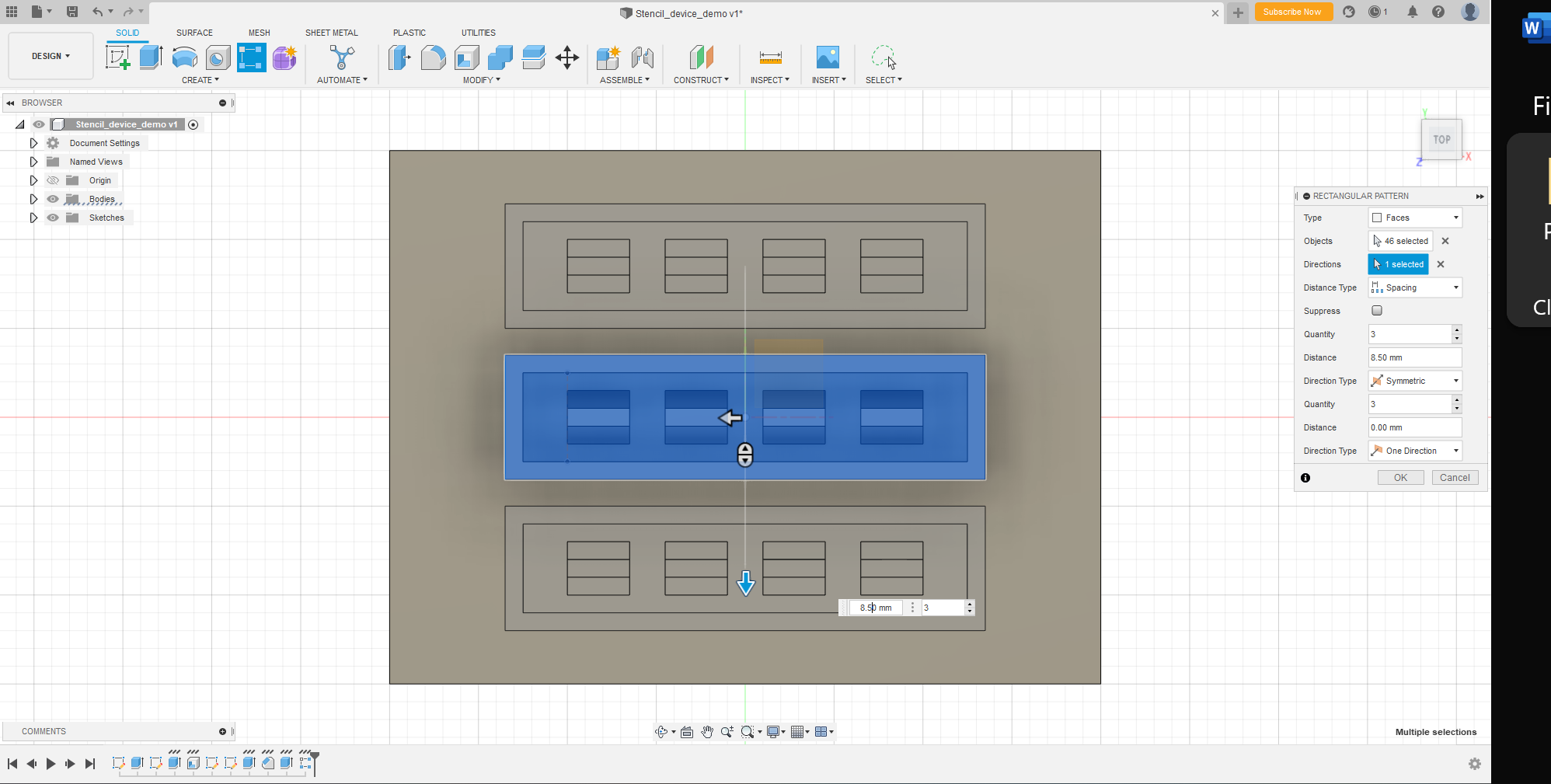


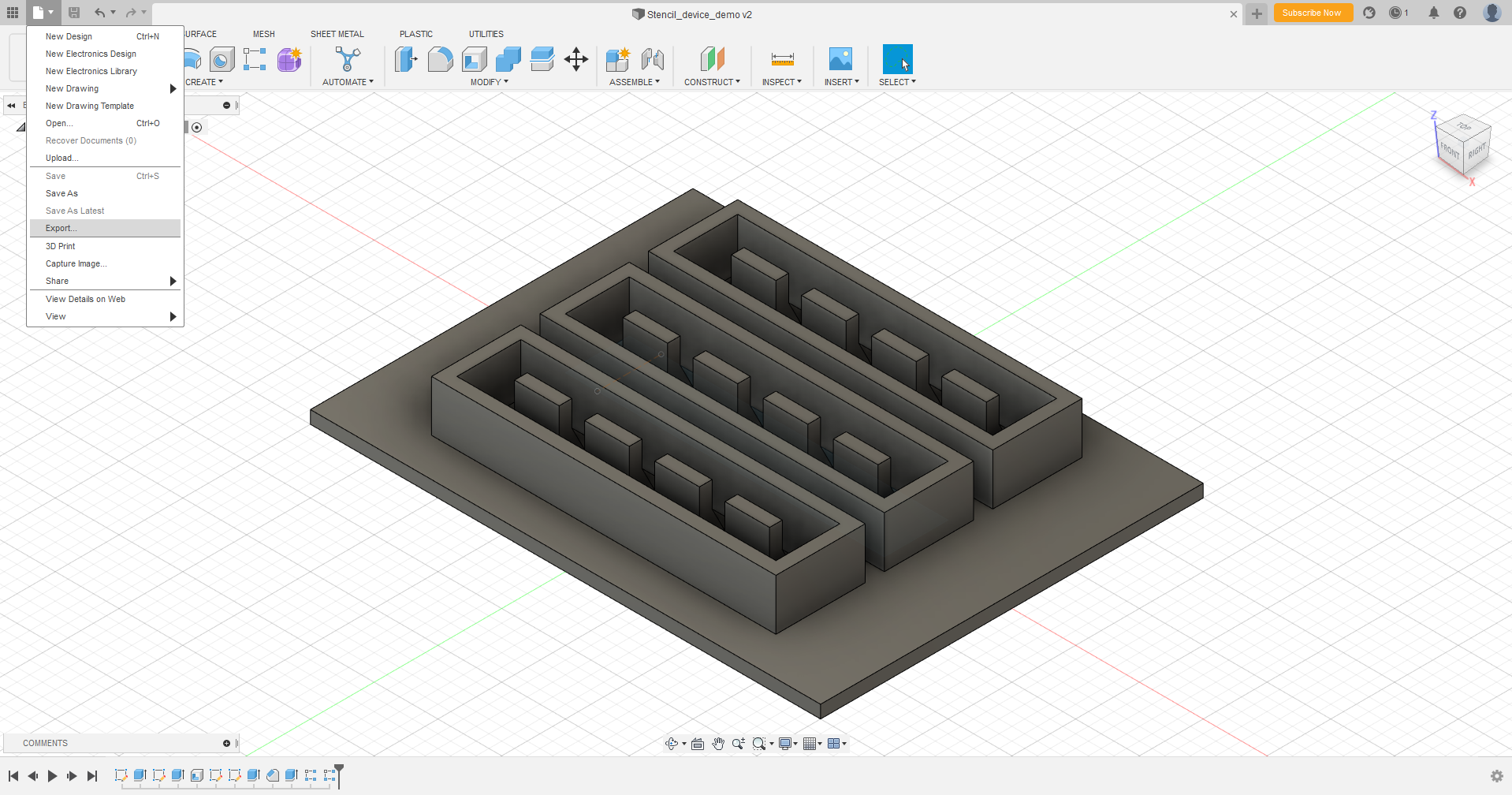


Above is the completed PDMS stencil device mould, complete with 12 3.5 mm X 1 mm wells spaced 2 mm apart, and a 2 mm deep funnel on top to facilitate easy cell seeding – concluding the design tutorial.

Once a design is complete it needs to be exported to an stl. (Stereolithography mesh) to be sliced and read by the 3D printer. This can be done easily in Fusion360 with File>export>.stl (below).


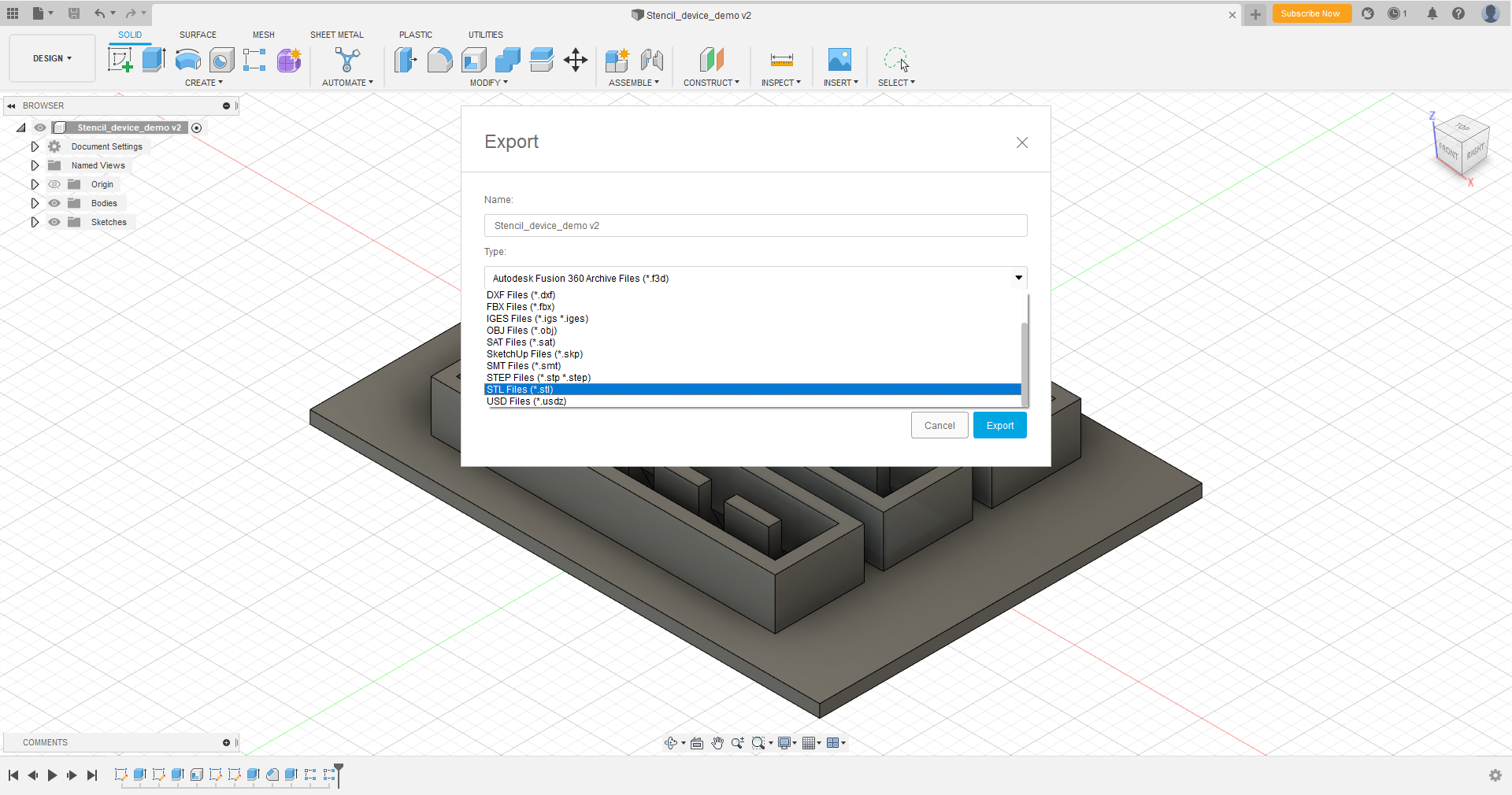


NB: Be sure to continually save your designs as you go, Fusion360 does not autosave. Conveniently, however, each save point can be recovered so if certain alterations don’t work you can always go back. Rolling the history marker back also helps to modify designs without starting from the beginning.

If you have any doubts about how to use Fusion360 don’t forget there are several online sources of tutorials such as the Product design online [channel](https://www.youtube.com/c/ProductDesignOnline). These tutorials can also enable you to design more complex, multi-part constructs to suit your individual needs.

## 3.0 Slicing

**Softwares**

Once a design is finalised and you’re ready to print you need to process the stl. Design file into slices to be printed layer-by-layer by the printer. This is achieved with a slicing software. There are several different slicers available, however the most used are Chitubox for SLA printers and Cura for FDM printers.

**Chitubox**

Chitubox is an easy-to-use slicing software with support for most SLA printers and resins built in, and a host of basic model manipulation functions to help get the best print possible. After importing a design, Chitubox allows the manipulation of orientation, scale, positioning, cloning of parts. As well as some more invasive functions such as shelling parts or drilling holes in designs. See below the window set up of Chitubox 1.9.1.


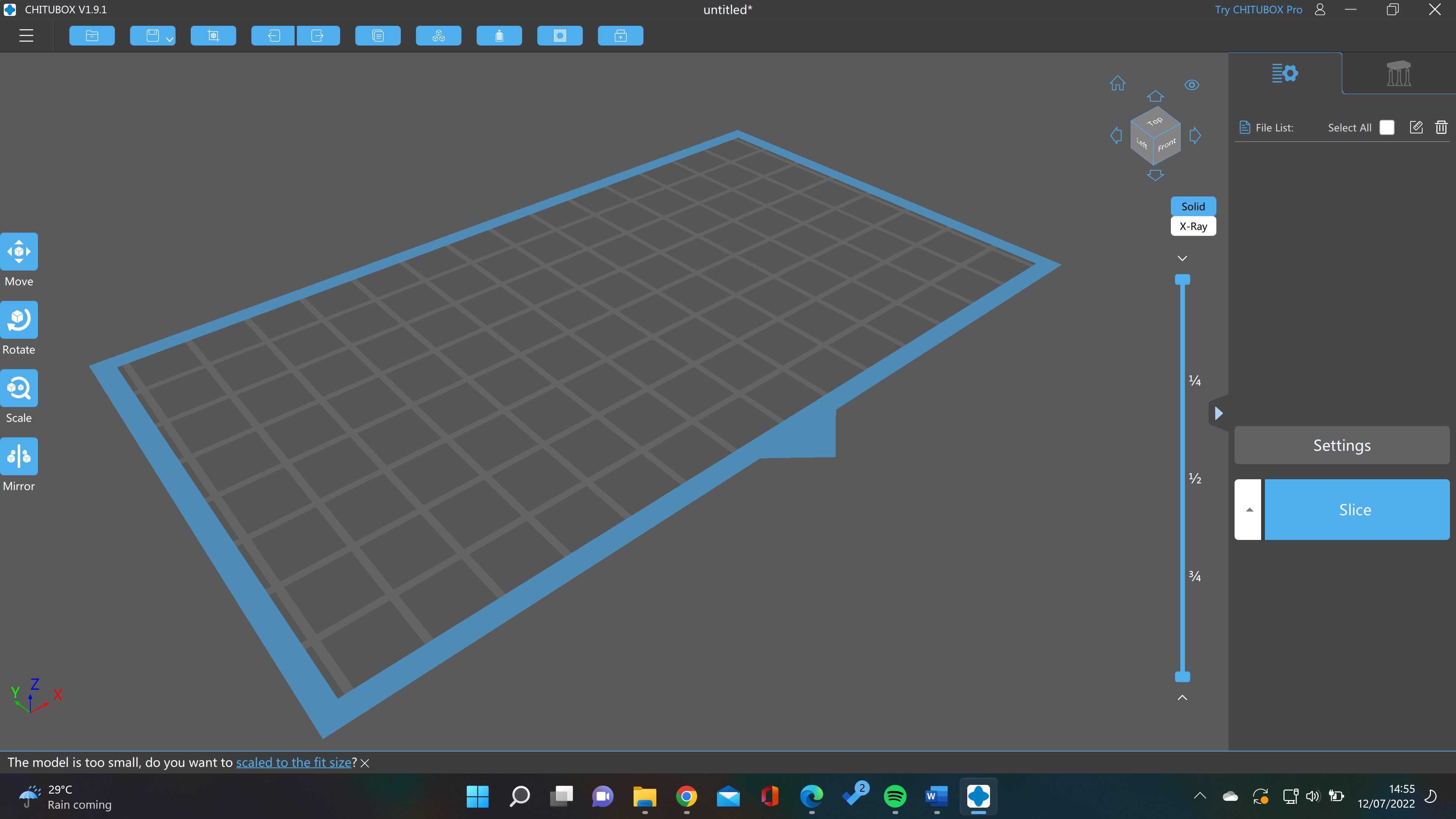


To correctly set a print going using Chitubox there are 3 things to consider.

- Print orientation/supporting
- Balancing the build plate
- Resin settings

**Setting up a printer for the first time**

If you have not used a printer before you need to set it up in the chitubox ‘settings’ tab. As chitubox is the main supplier of boards for most SLA printers they support a wide range of printers and resins with manufacturer published default resin settings. You need to select ‘add a printer’ and scroll to the system you have. For the Serio lab this will either be the Phrozen sonic mini 4K, or the Elegoo Mars 3. You can have any printers you use saved in your ‘settings’ and add/remove more as you please.

NB: New printers and resin settings are updated with chitubox updates, make sure the software is as up to date as possible.

NBB: As chitubox software updates roll through sometimes printers stop supporting the sliced file type. If this happens it will most likely be fixed by a printer firmware update. These are slightly tedious but will be explained in detail on manufacturer websites if required.

**Print orientation/supporting**

Once you have added your printer you and imported a part by dragging in your .stl file you need to consider the orientation of your part, and whether it needs supporting. To do this you can use the ‘move’, ‘rotate’, ‘scale’, and ‘mirror’ tools to change the location, scale, or orientation of your print – see below. Print orientation becomes extremely important for printing parts with microfeatures such as grooves where features are acutely impacted by factors such as pixel shape and orientation, grooves are printed at 90°.


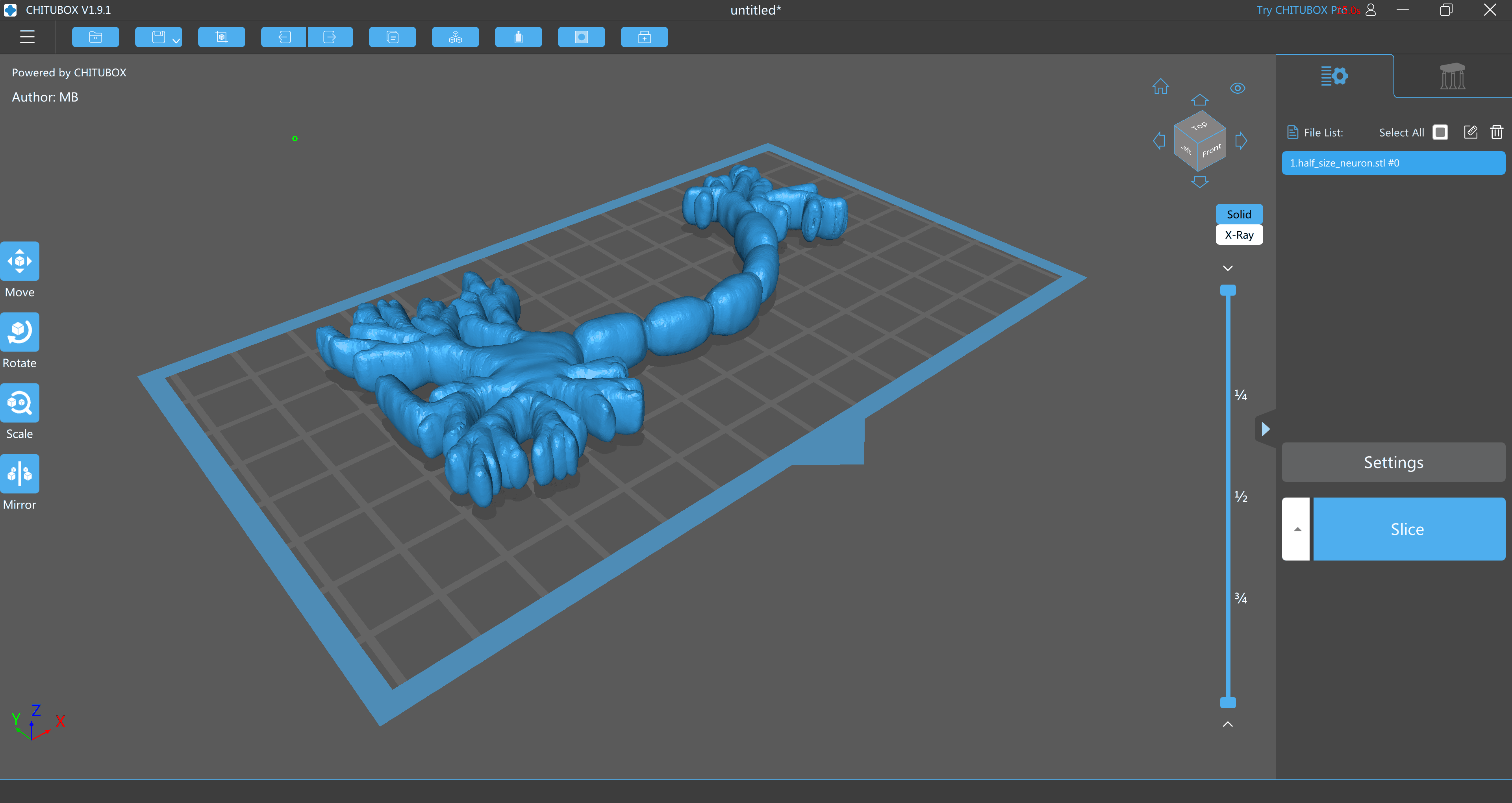


Once you are happy with the size and position of your print, you need to consider whether you need to support it or not. As prints are constructed layer-by-layer it is important to identify and support parts of your designs that over overhanging or are not flat as these areas cannot be printed without additional supports. When supporting parts, it is also good practice to angle designs to limit the risk of failure of overhangs, although this will reduce print resolution in some cases – lower layer thicknesses will help negate these losses, at a trade off with print time. See below a print requiring angle changes and supports.


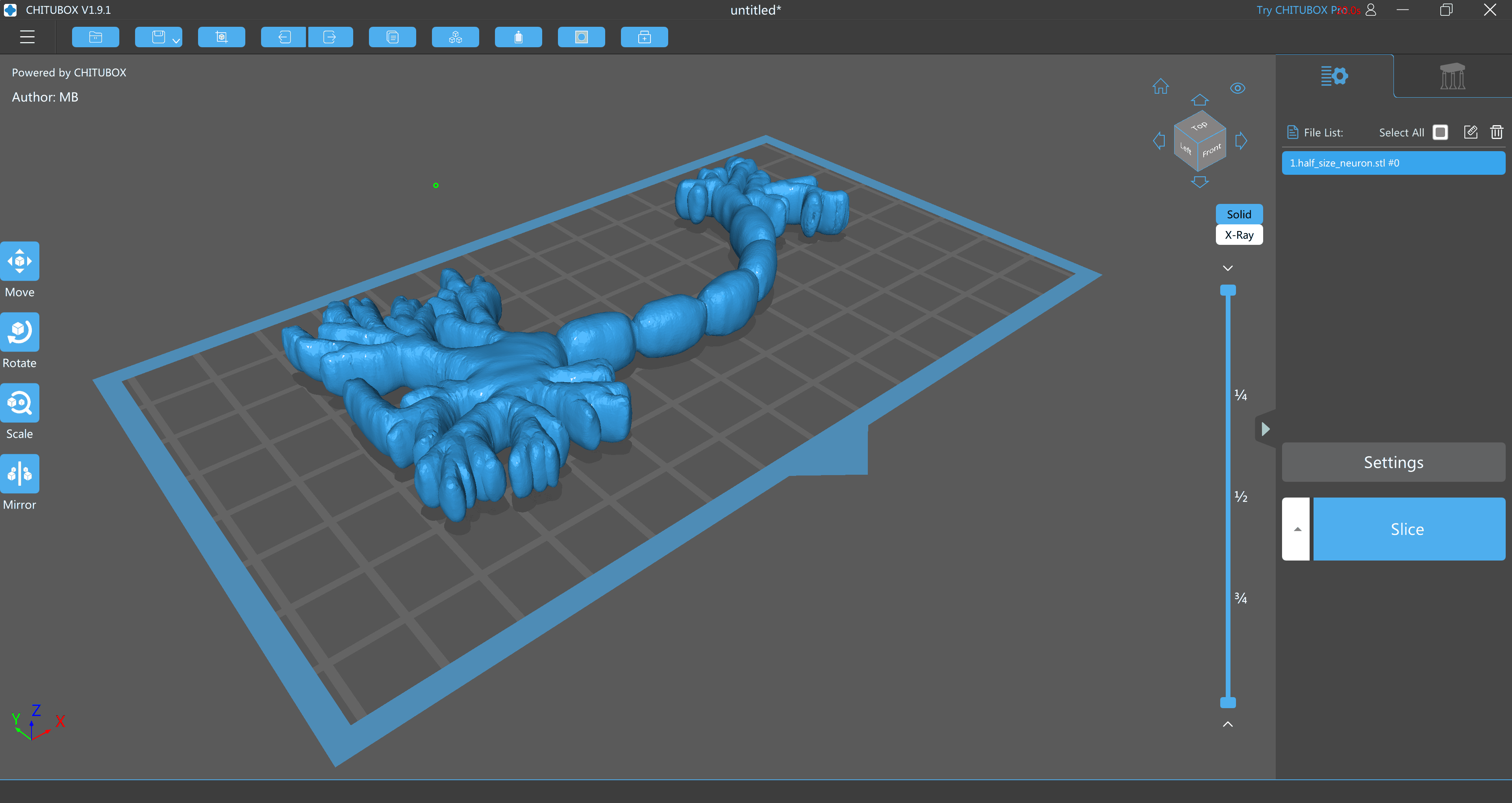


Here it is important to note that the LED array has rectangular pixels, so isotropic designs print better when aligned with the array. Meaning that regular elongated pattern such as grooves should be printed at 90°, compared to round, dot-like features which appear the best at 0°.


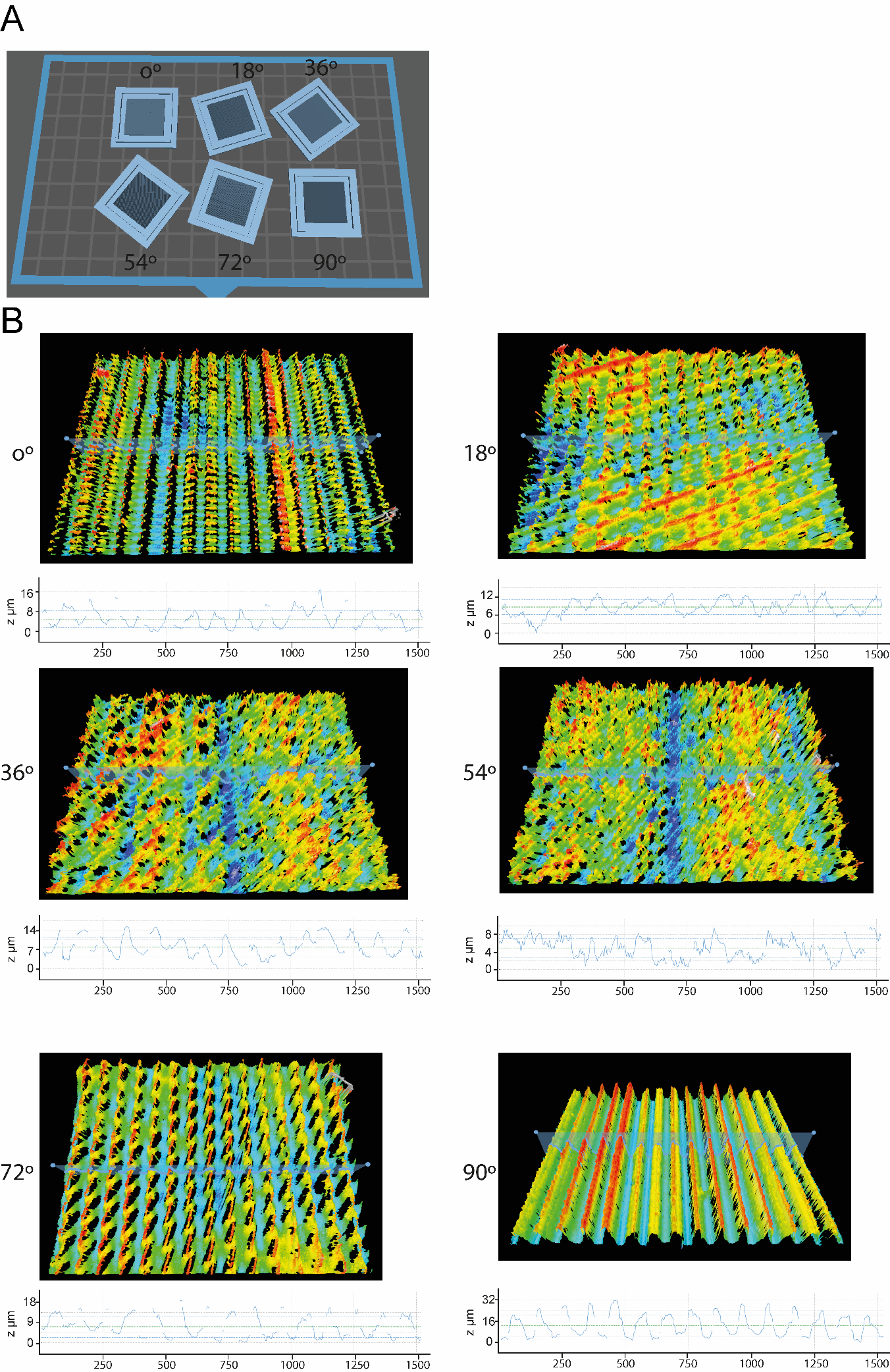


**Balancing the build plate**

After finalising the supports for a print, you need to check that the build plate is balanced. This is important as unbalanced prints will result in differential pulling force on the FEP film and build plate when retracting from each polymerised layer, increasing the risk of FEP rupture but also compromising print resolution. For large/single prints it is important to centre the part on the build plate with the move tool. For multiple prints it is good practice to organise the build plate in a symmetrical fashion, with the tallest parts in the centre.

**Resin settings**

Finally, you need to select the appropriate resin settings for your chosen printer, resin, and layer thickness from default or optimised parameters in the ‘settings’ tab. Understanding these parameters can help in fine tuning print quality for a specific print, or for optimising a new printer/resin.

In short, the settings can be grouped into 3 types: exposure, light off delay, and lifting speed/distance. With ‘bottom exposure time’ dictating how well adhered prints will be to the build plate. ‘Exposure time’ dictating the layer thickness (how much resin is polymerised for each specified ‘layer height’). And the coupling of lifting speed/distance with ‘light off delay’ dictating how far and fast the build plate is moved up and down between each layer, and how long the LCD waits before re-illuminating. In the lab, our experience has found that ‘exposure time’ has the most significant impact on print quality variation, and that slower lifting speeds (and associated light of delay) will increase the quality of prints (especially for higher viscosity resins) at the expense of print speed. ‘Transition layers’ are a series of layers with decreasing exposure time between the bottom layers and the normal layers, providing better adhesion of the top and bottom layers of your print. Whilst they can help limit the ‘elephant foot’ phenomena that occurs from wider resin polymerisation at the base of prints from the high exposure, they are not compulsory. A repository of the current optimised print parameters for resins can be found at the end of this section. A light of delay calculator can also be found in the github repository to help if modifying settings from default.

NB: New updates to chitubox can sometimes alter the structure and parameters in resin settings so it is important to keep track of changes in case they alter PDMS compatibility or print quality.


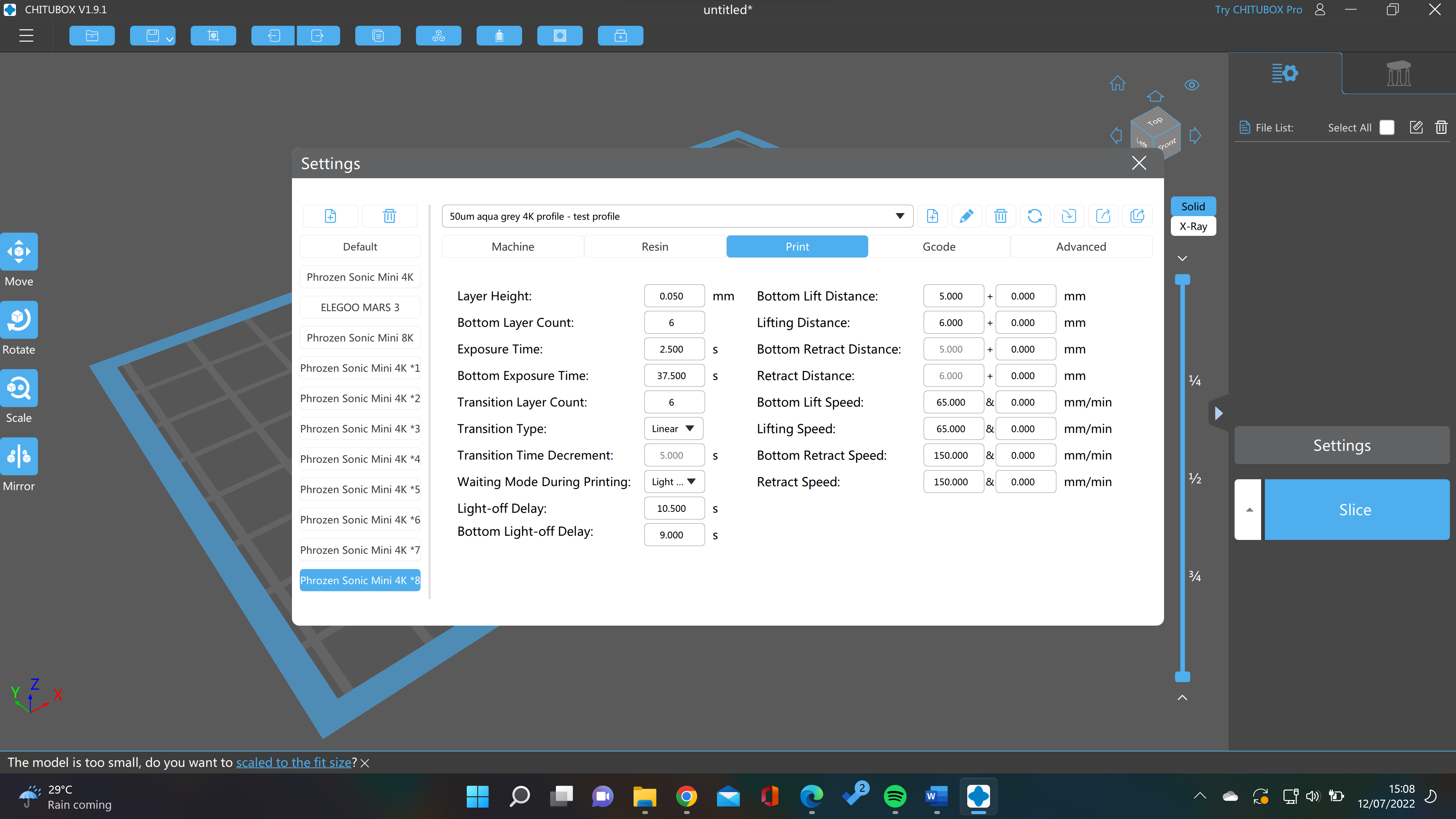


All you need to do now is hit the ‘slice’ button and save your sliced .ctb file to a virus free USB stick. Once in the slicing screen you can also see an estimate of the consumable’s usage of each print, the print time, and a reconstruction of each printed layer. This reconstruction is a good opportunity to double check for any unsupported islands on prints. There is also a brief summary of the resin settings at the bottom of the window which is also helpful to check you have selected the correct parameters.


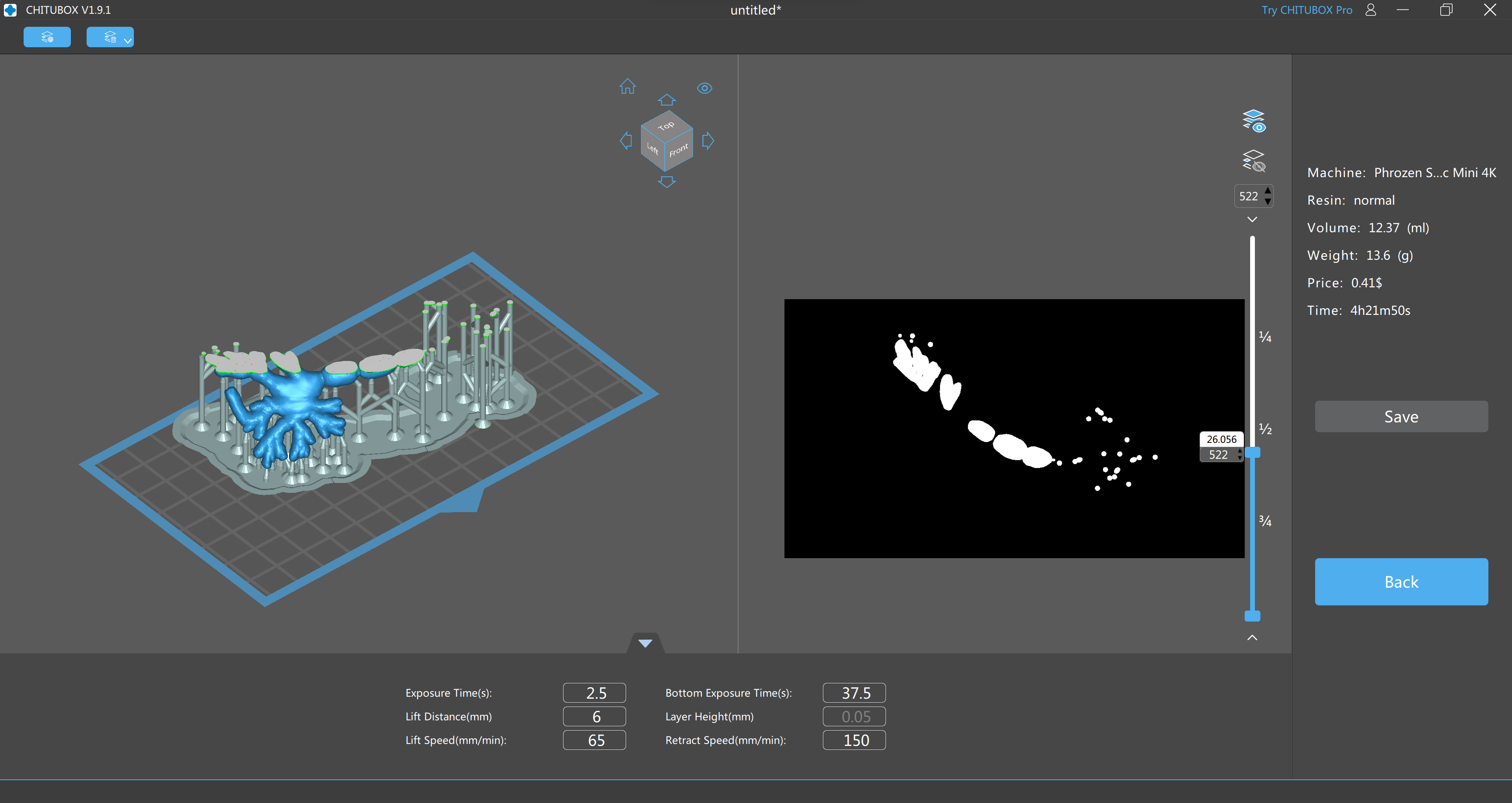


## 4.0 Printing

**Preparing to print**

**Cleaning**

Before starting a print, it is important to check the printer is clean, this will help ensure print quality, reduce the chance of failures, and increase the longevity of the system. Regardless of printer type there are several important components to be checked before starting to print:

Build plate

Check there is no residual resin on the build plate from previous prints and clean off. If left unchecked resin can be partially cured from exposure to natural light when the printer is open and can result in poor print adhesion to the build plate. If particularly worried, use IPA to help remove resin.

Resin VAT and LCD Screen

VAT – **EMPTY** – if the printer hasn’t been used in a while, or you are printing in a different resin, ensure you clean the inside and outside of the resin VAT thoroughly. Do this by wiping down the exterior (with or without IPA) and gently wiping the top and bottom of the FEP film with a tissue (softer/finer than paper towel, also with or without IPA) to remove any dust/residual resin.

VAT – **FULL –** If the printer is being used with the resin already present in the VAT it is important to check there is no solid debris left in the resin from previous prints. If there are, this will likely indent or rupture the FEP film during the next print, potentially causing damage to the printer from resin leaking from the VAT into components. Check for resin debris by gently running the mixing spatula through the resin (this will also mix the resin): feeling for increased resistance – parts polymerised to the FEP film, or for solid objects that appear at the surface when moving through the resin. If debris are found ensure to remove them and check thoroughly that there are no more present. For parts on the FEP film, try to remove them with gentle pressure using the spatula (be careful not to damage the FEP) and repeat the check for debris. The best way to remove all debris is to change the resin in the bath.

Changing the resin

To change SLA resins, you need to drain and clean the resin VAT completely before adding the new resin. If there is a lot of resin left in the bath (more than 1cm depth) it is best to drain this into a ‘dirty’ resin bottle so it can be reused (do not mix different resins – have a separate bottle for each). If there is only a little resin left in the vat it is likely contaminated and not worth keeping. To clean waste resin all printers, have a vat cleaning function. This function uniformly illuminates the full build area for a user defined period, polymerising a layer of resin to allow it to be peeled off the FEP film with ease and can be disposed of safely – leaving a clean vat behind. A normal exposure is 20 seconds and will allow polymerisation without fusion of the resin to the FEP film. A caveat of the vat cleaning function is that the screen is by design smaller than the area of the FEP film, so additional mopping of resin in the surrounding area is required. A tip here to prevent marking the FEP is to conduct the vat cleaning but mop excess resin from around the now-polymerised resin before peeling off the semi-polymerised resin. Once the vat is clear of resin you can add your desired resin to the bath.

**Mixing the resin**

Before printing all resins need to be **mixed** properly to ensure even distribution of photoinitiators and other resin components such as colour pigments. Without proper mixing, print quality will be varied and risk of failure is higher.

- Adding resin from the bottle:
  - The best way to mix resin to ensure good mixing whilst avoiding creating bubbles is to gently invert the resin bottle 5-10 times before pouring into the vat.
- Mixing resin in the vat:
  - Use a plastic spatula to gently mix the resin without touching the FEP film. Look out for changes in resin colour and distribution of pigments as an indicator of mixing.

**Getting rid of bubbles**

In many cases, in particular with more viscous resins, bubble formation during mixing is unavoidable. Bubbles in the resin can result in print failure so it is very important to remove as many of them as you can before printing. The best way to remove bubbles from the resin is to let the resin sit for 10-15 minutes before starting a print to allow them to surface and disappear. If you are short on time the tip of the spatula (or razor/ pipette tip) can be used to gently remove bubbles at the surface of the resin manually.

**Securing the resin bath and build plate**

The final step before setting a print going is to check that the resin vat is firmly secured to the printer by checking the screw on each side is tight. It is important for print resolution to have the vat as tightly secured as possible to the printer to ensure even distribution of forces over the FEP film on retract and uniform exposure of resin across the bottom of the bath.

**Setting a print going**

Once you have the printer cleaned and resin mixed, turn the printer on, plug in the USB, find the file you would like to print, and press go!

## 5.0 Post Processing

Post processing of resin prints serves 2 purposes, to **wash off** excess resin left after printing, and to **finish the UV polymerization** of the resin in its final design.

**Removing prints from the build plate**

After print completion and before post processing can begin you need to remove the part from the build plate. Depending on the resin you printed with there will be excess present on the build plate, remove this with the plastic spatula back into the resin vat before removing the build plate from the print. Once most of the excess resin is removed you can detach the print from the build plate using a combination of a razor blade and the plastic spatula – see below:


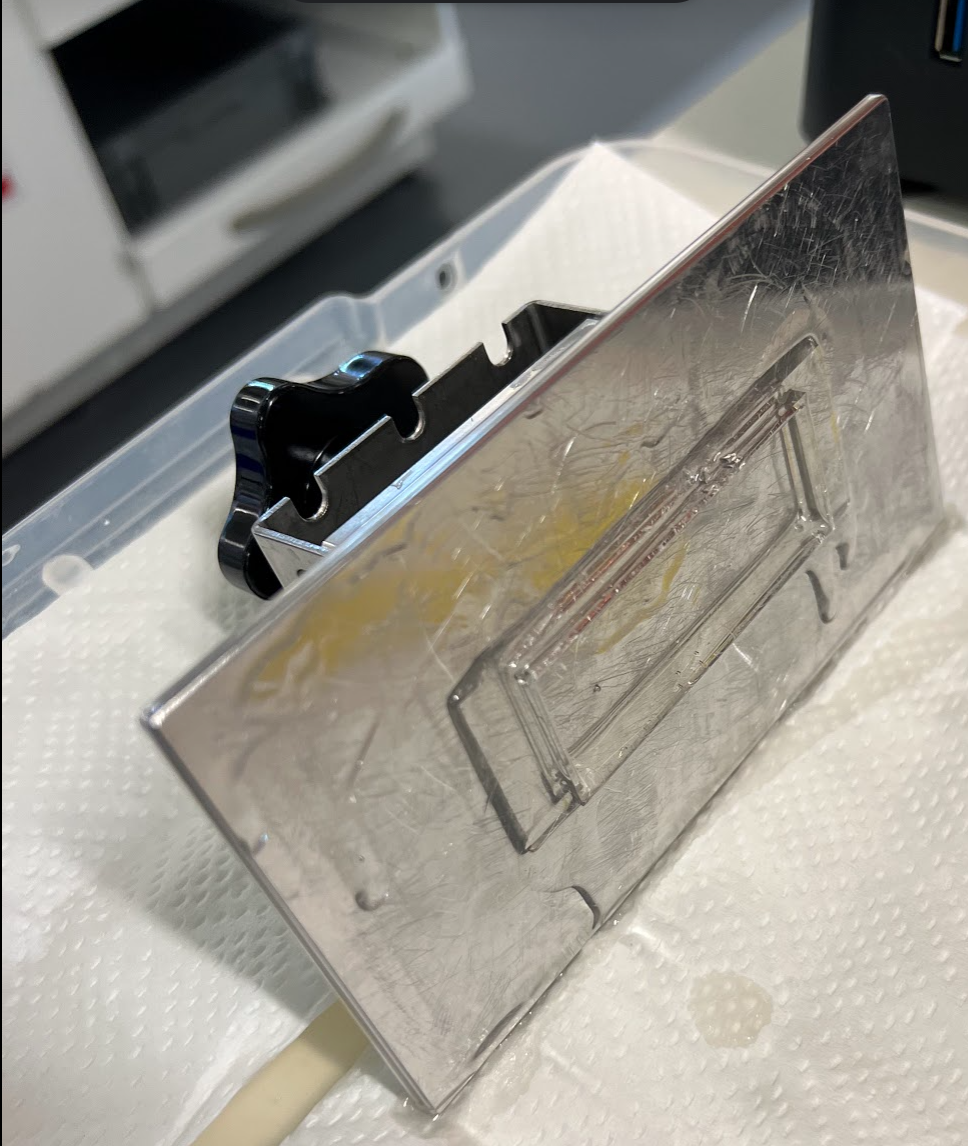

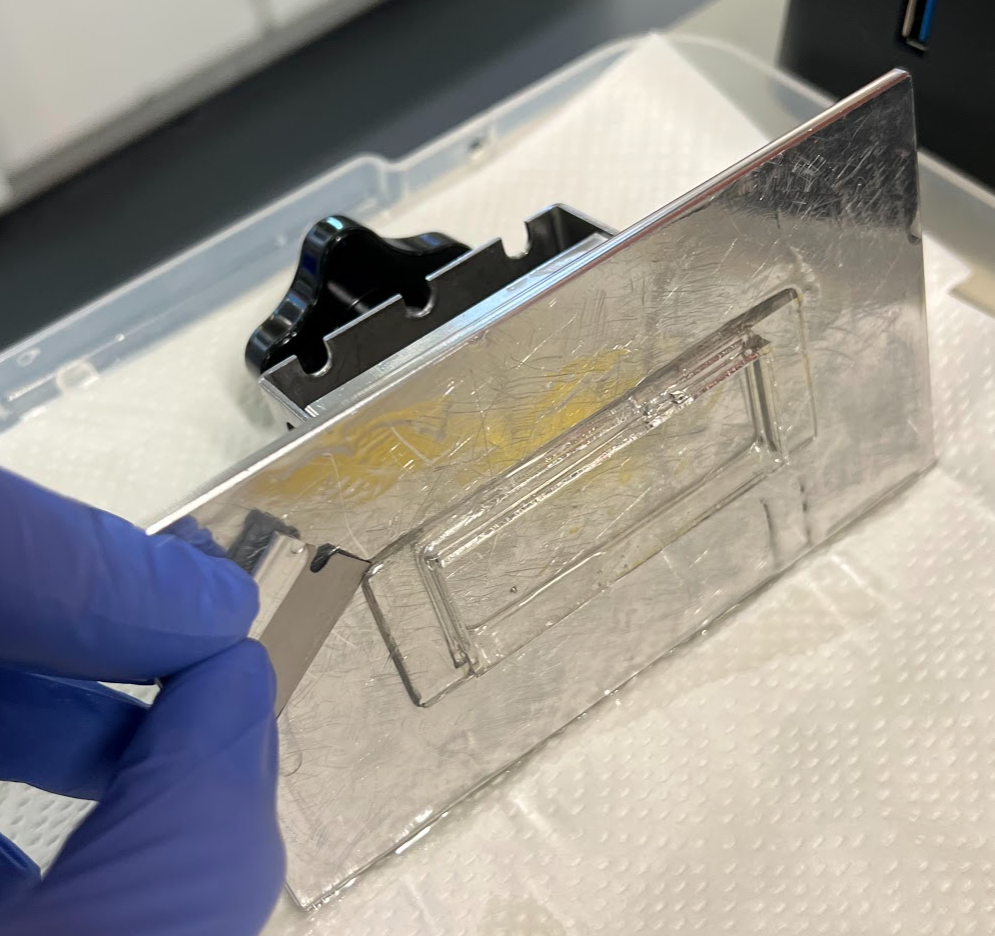

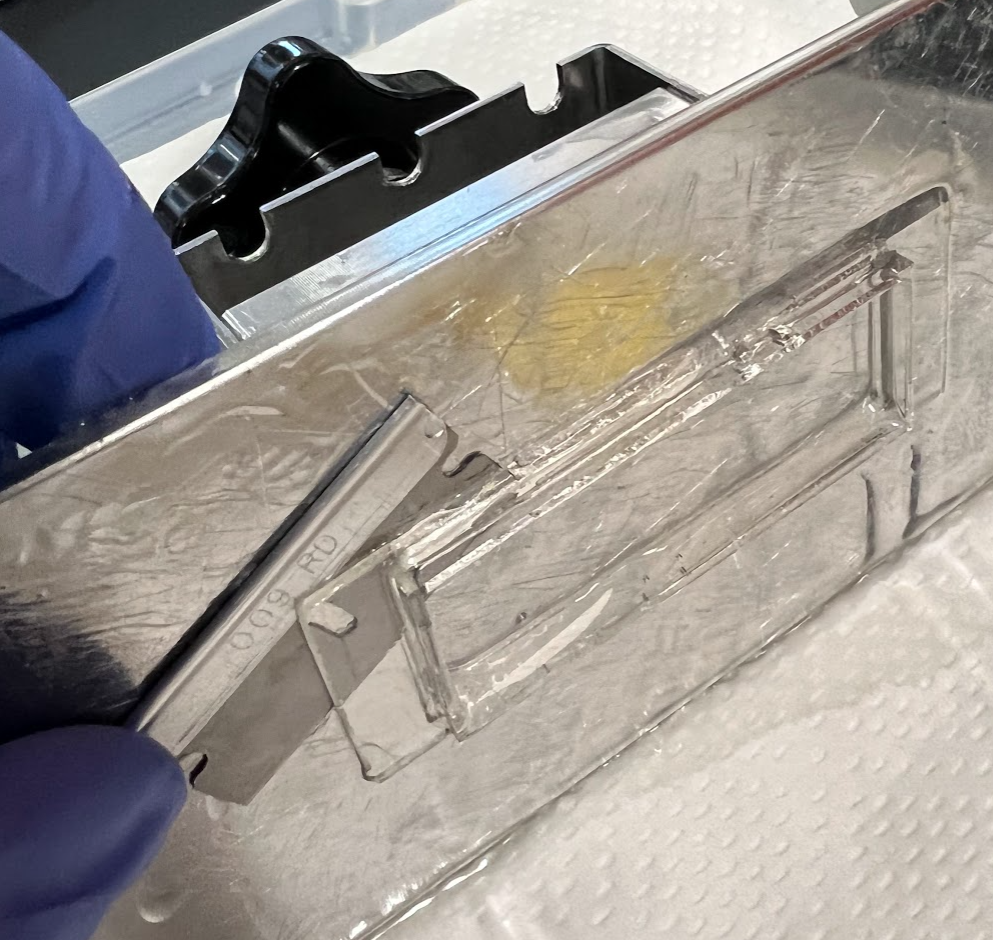

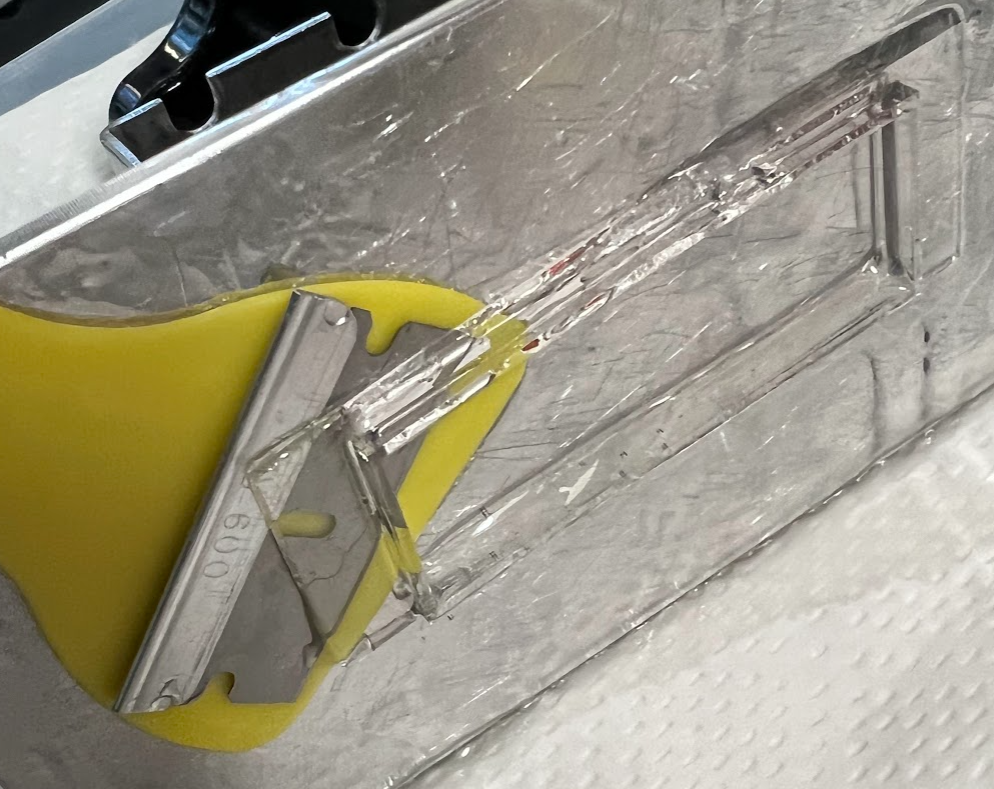


Prize up corner with razor

Push razor further under print

Slip spatula underneath and push rest of print off

It is important to prize up the part **gently**, firstly to prevent scratching or indenting the build plate which might impact the quality of subsequent prints, but also to limit the risk of destroying the not-fully-polymerised part.

See below the optimized protocol for all resin prints.

**Post processing – all resins:**

1. 10min in Sonicator in IPA (dirty wash) – in instrument room with -80 freezer
2. 10min in wash of wash & cure in IPA (clean wash)
3. Cure 1h (wash and cure system)


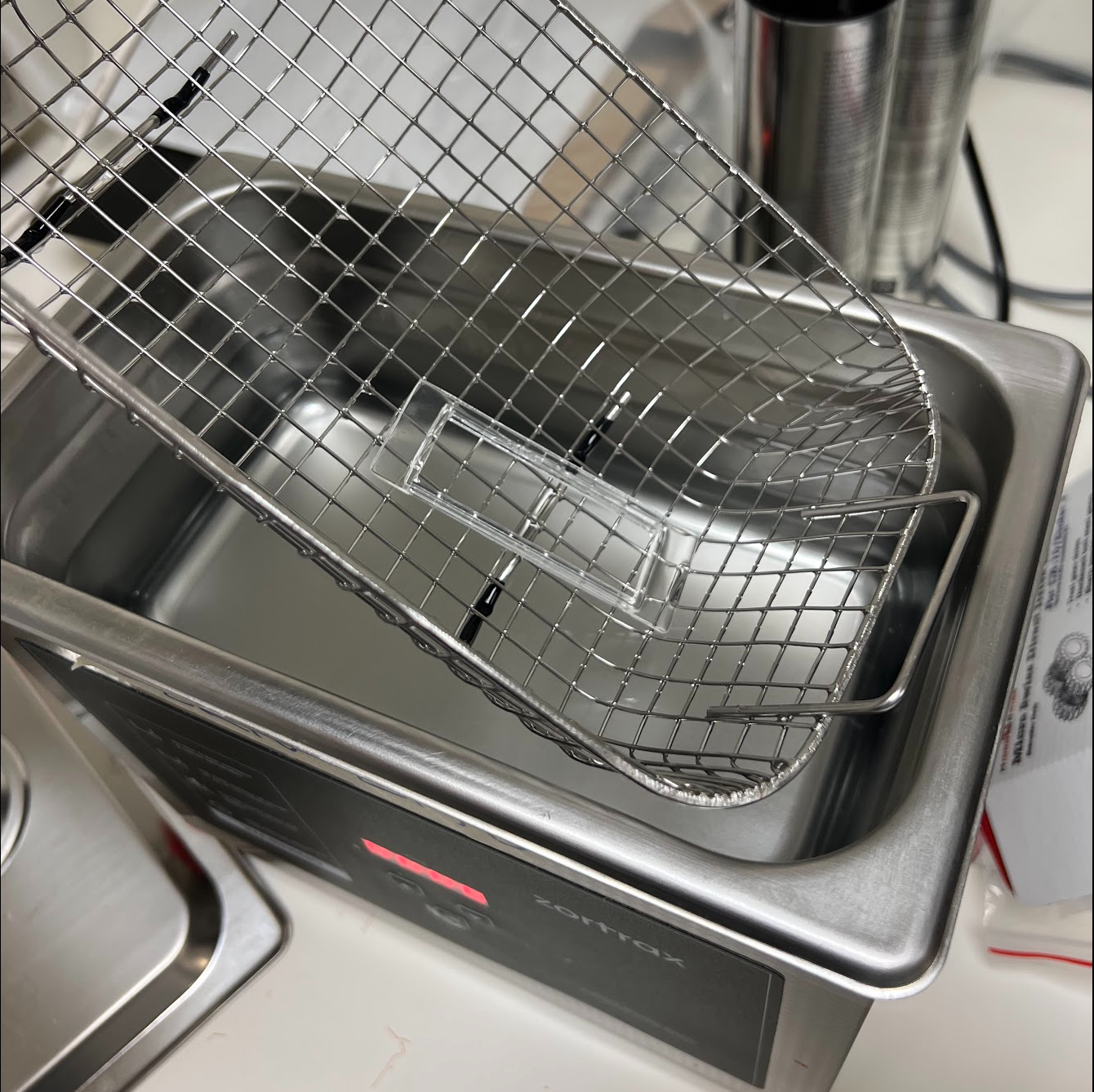

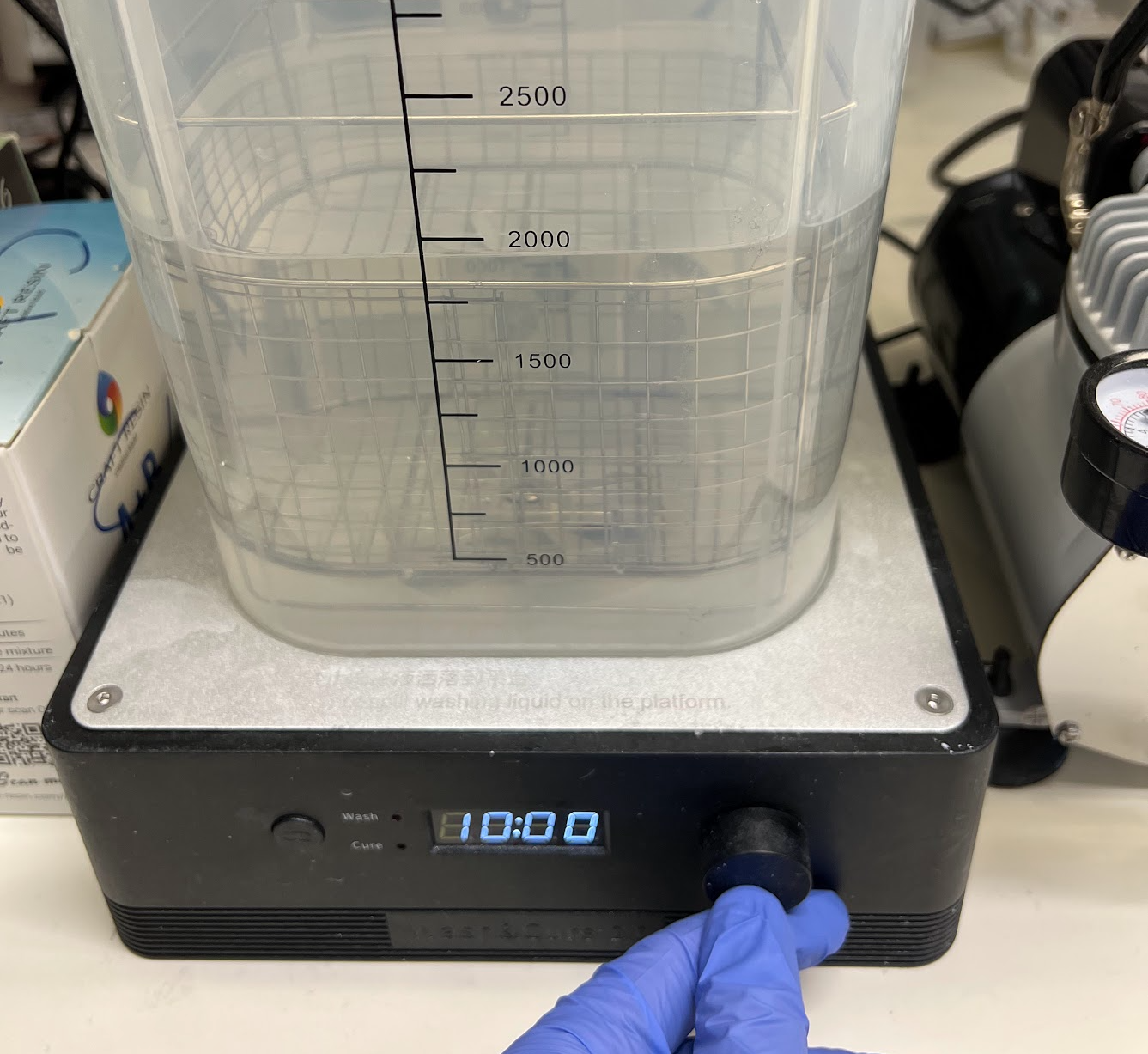

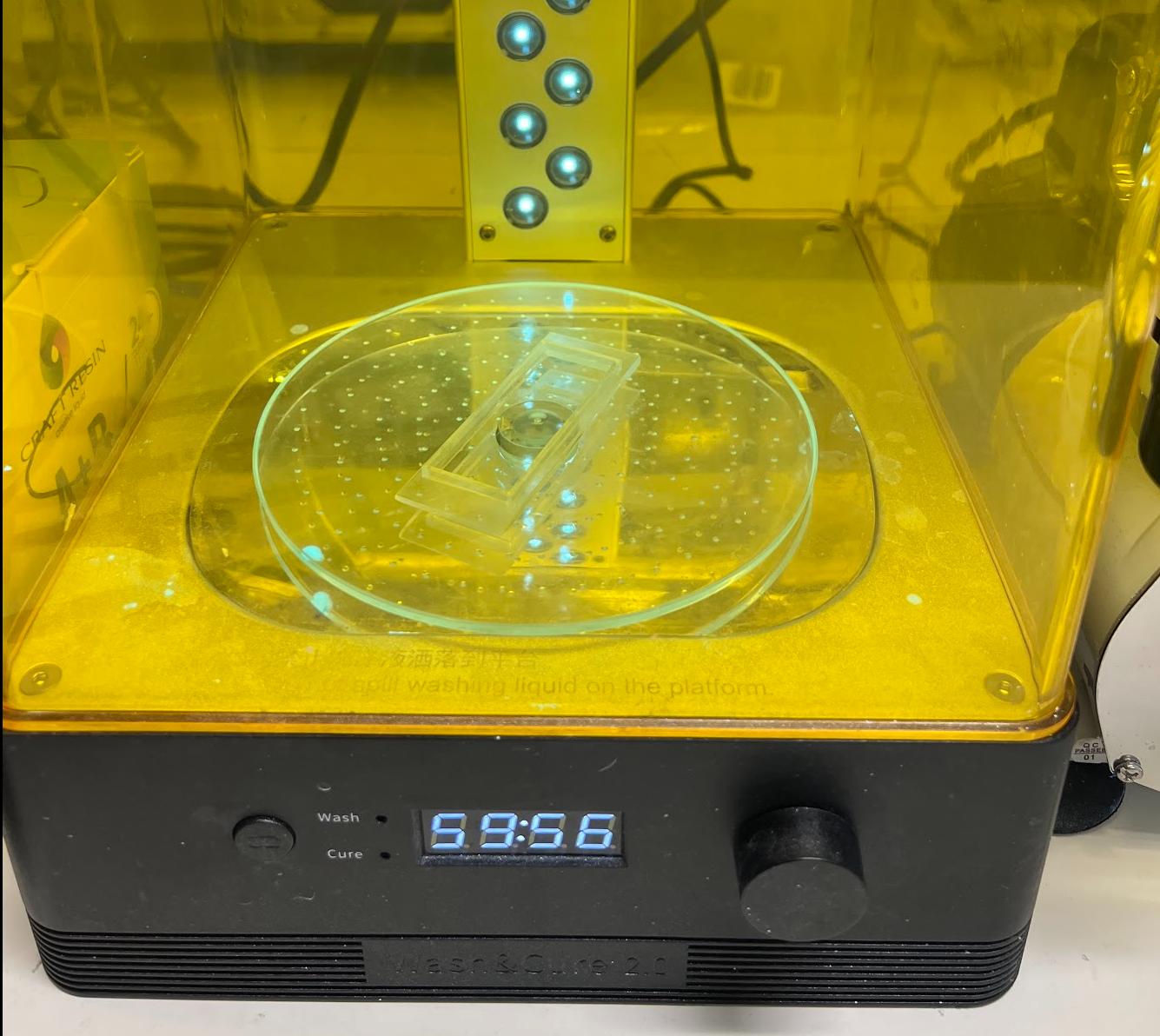


10 mins sonicator (dirty wash)

10 mins wash (clean wash)

1 hour UV curing – ensure parts are completely dry of IPA before curing

**Notes**

- For biocompatible resin use **fresh** isopropanol not filtered
- If printing very small <250 µm features, less/no sonication can be performed to preserve the delicate features. If extremely worried, conduct manual washing with IPA, although this will impact the curing of PDMS if casting is required
- **IMPORTANT – DO NOT** leave prints in lPA for prolonged periods as overexposure can result in warping. Warping is worst from the sonicator as the IPA here gets hot (>50°C after use).

**Deciding which pipeline to use**

We developed two strategies for casting PDMS in 3D printed moulds, which you can find highlighted in the main manuscript Figure 1G. The main difference between these two protocols is the additional coating with enamel paint after the post curing process. The two main contributing factors to decide which pipeline to use, are the size of the features and the type of mould. As a rule of thumb, complex and closed moulds with little air contact, such as sandwich moulds used for figures 8 and 7 will require the additional coating. Additionally, if your designs do not contains small scale features <100um, it is worth it to immediately use the paint protocol shortening the troubleshooting process. But this decision has to be taken for each design individually. We added here some design examples that take these points into consideration

| Coated pipeline | Non-coated pipeline |
| --- | --- |
| 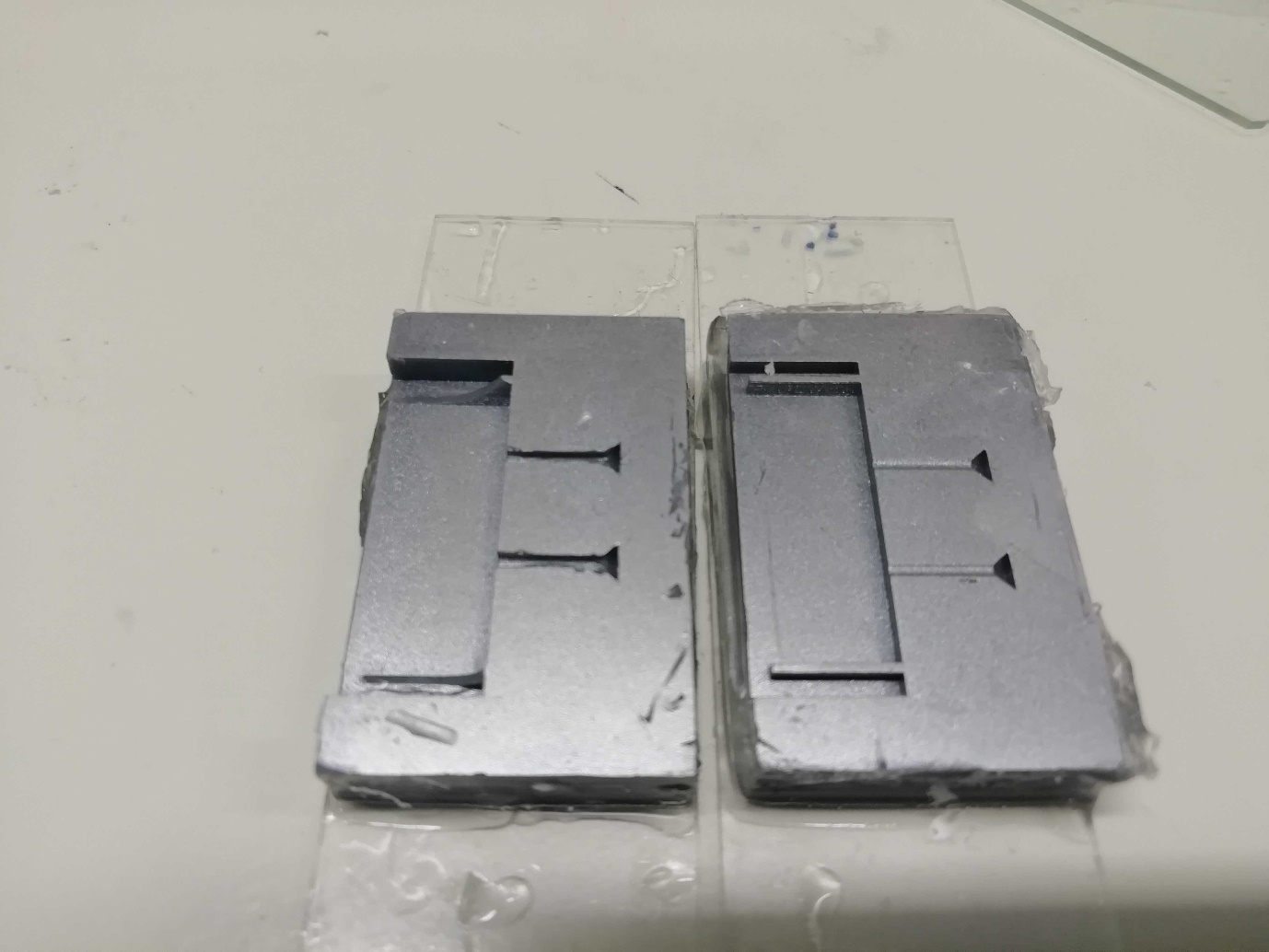  Muscle device mould: A complex mixture of small and large-scale features in a sandwich mould. There is limited air contact, a large contact area and complex features that make the demoulding itself challenging. The mould has been enamel coated, even in the small features, through angle variation while air brushing. | 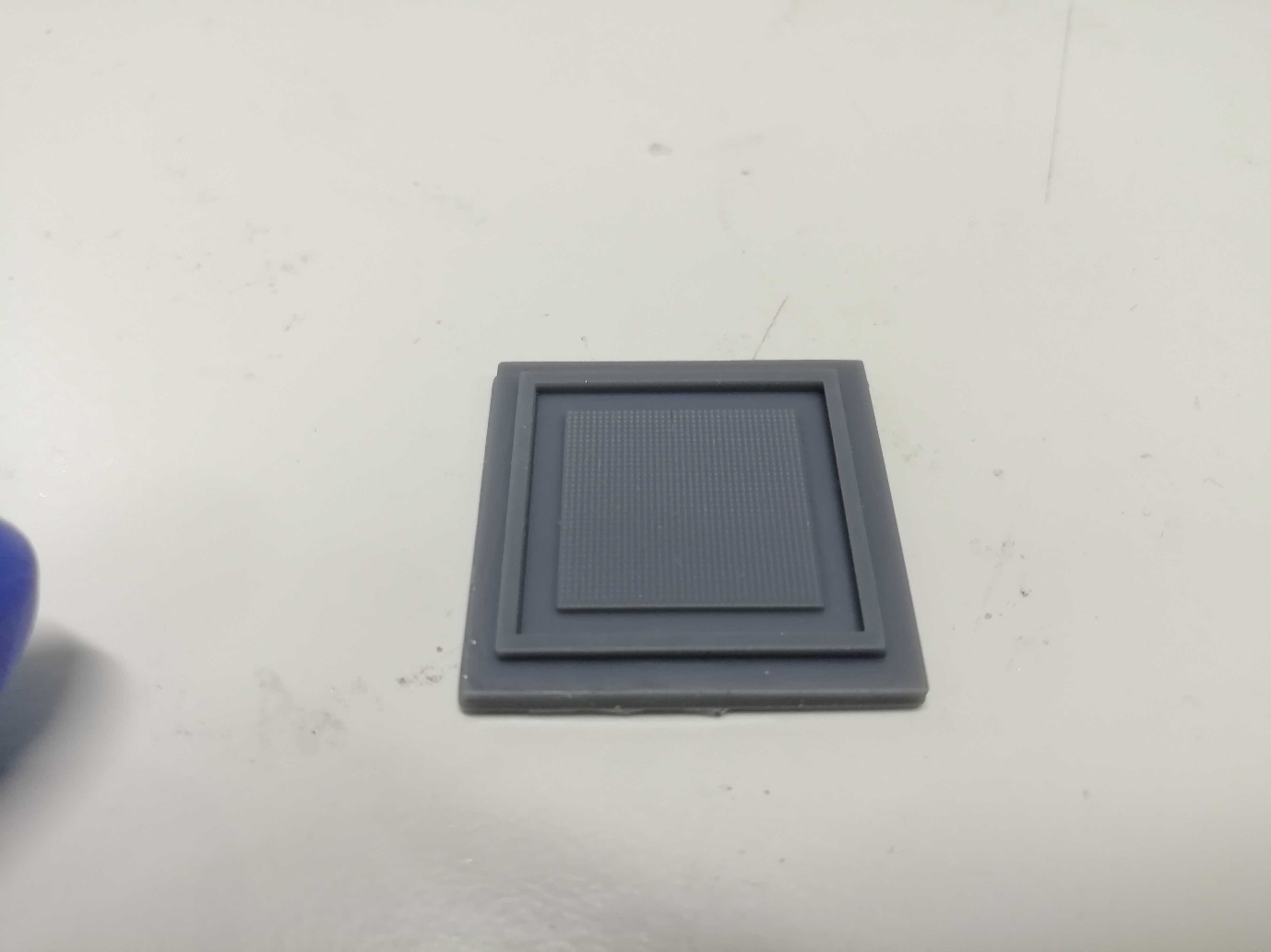  Microwell mould: This moulds has a large surface area but only small features. Here, we used the non-coating pipeline, so that the features remain at their original size. |
| 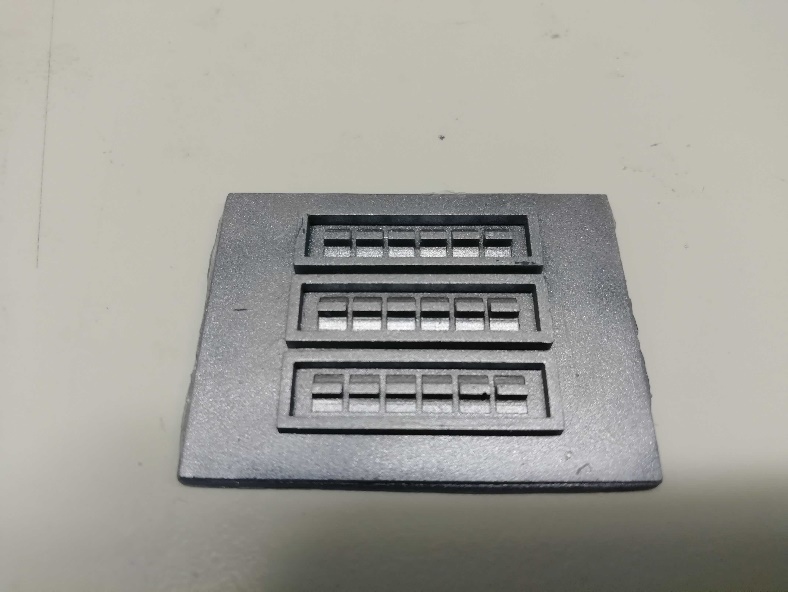  Plating device mould: In this case, we decided to use the painting protocol, as no feature will be covered and the thick layer of PDMS can cure better. This might be also possible with the non-coating protocol but for better demoulding we decided to use the coating pipeline. | 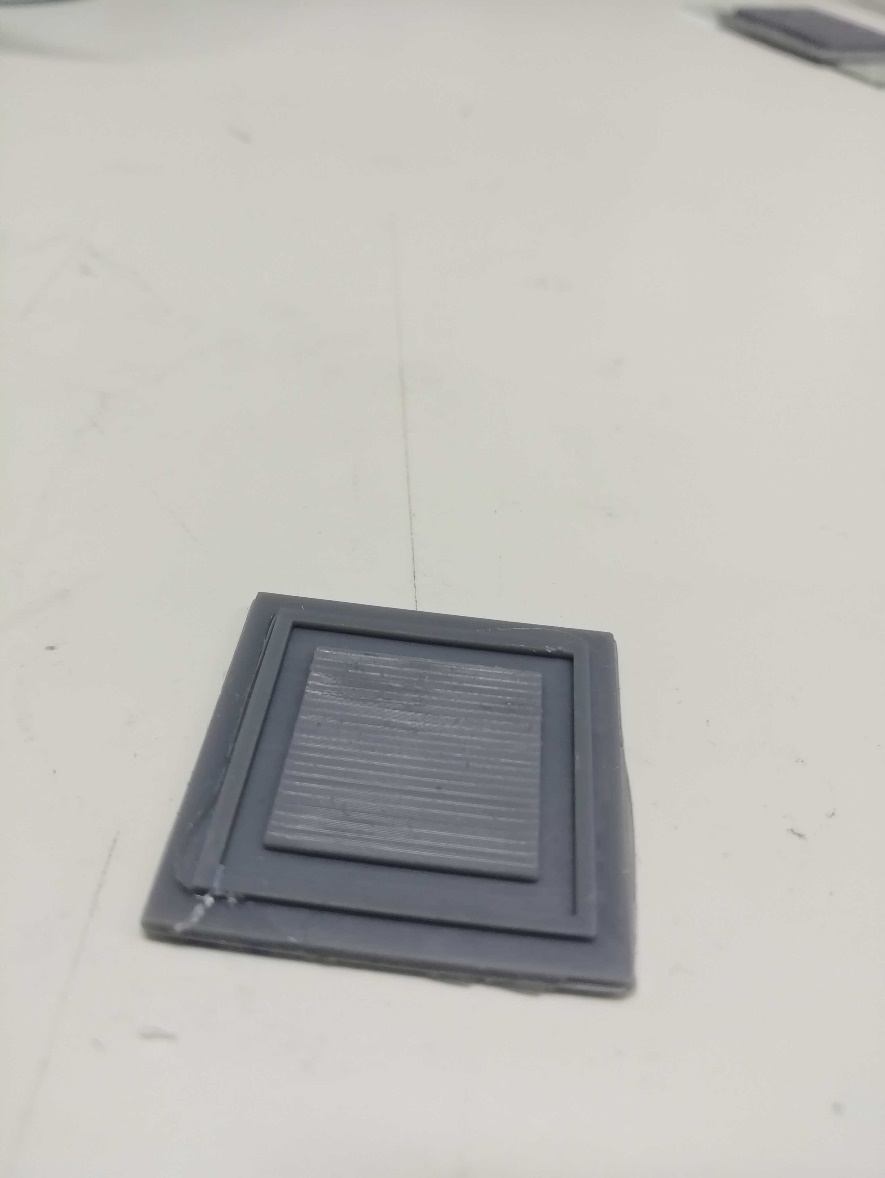  Groove mould: This mould has a large surface area but only small features. Here, we used the non-coating pipeline, so that the features remain the original size. |


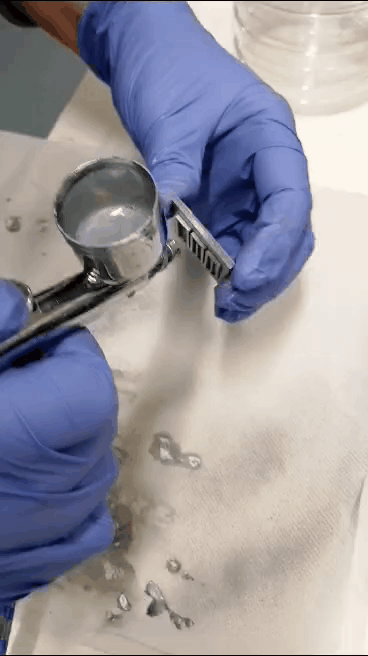

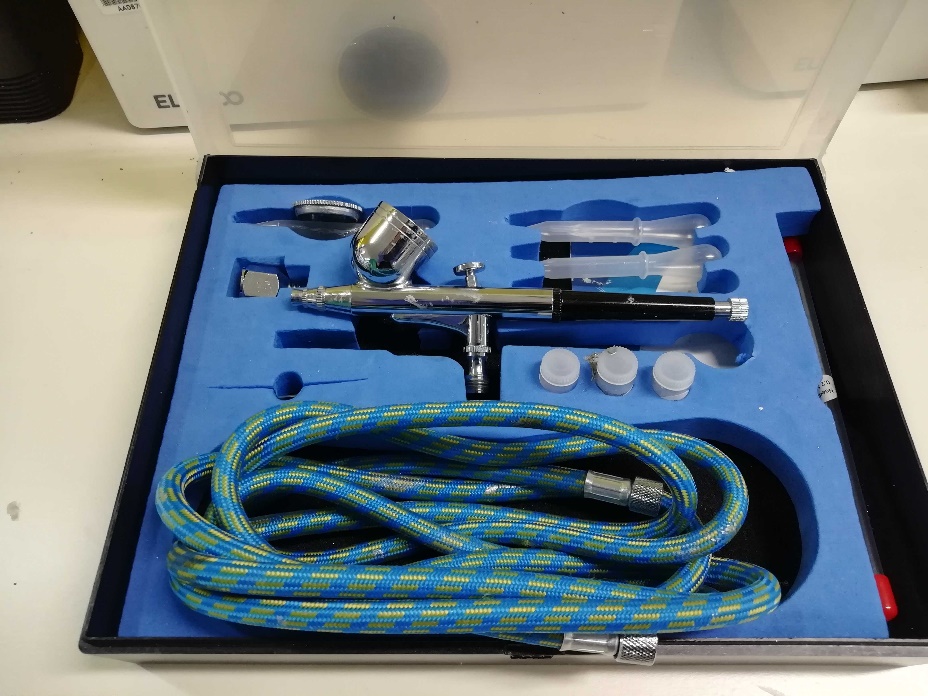

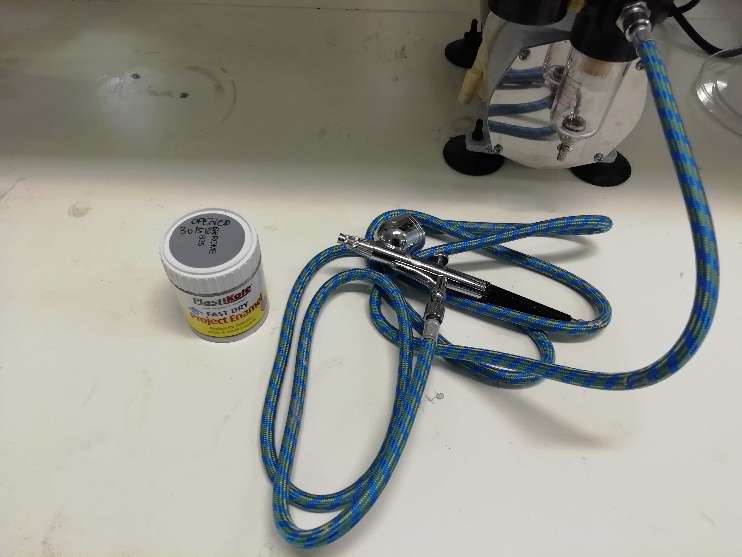
 To equally distribute the enamel paint on the surface airbrush system is of great help and if it is carefully done and the print rotated multiple times from different angles, on can reach even into deep and complex features. In our experience if paint does not reach the area, the design is too complex and the PDMS will not demould properly even if it would cure.

1. Soft lithography for micro- and nanoscale patterning. <https://www.nature.com/articles/nprot.2009.234.pdf>.

2. Qin, D., Xia, Y. & Whitesides, G. M. Soft lithography for micro- and nanoscale patterning. *Nat Protoc* **5**, 491–502 (2010).

3. Rammohan, A. *et al.* One-step maskless grayscale lithography for the fabrication of 3-dimensional structures in SU-8. *Sens. Actuators B: Chem.* **153**, 125–134 (2011).

4. Waits, C. M., Modafe, A. & Ghodssi, R. Investigation of gray-scale technology for large area 3D silicon MEMS structures. *J. Micromechanics Microengineering* **13**, 170 (2003).

5. Think big. Print nano. Your partner for high-precision additive manufacturing. <https://www.nanoscribe.com/en/>.

6. UpNano. <https://www.upnano.at/technology/#scale-applications>.

7. Gong, H., Beauchamp, M., Perry, S., Woolley, A. T. & Nordin, G. P. Optical approach to resin formulation for 3D printed microfluidics. *RSC Adv.* **5**, 106621–106632 (2015).

8. High Resolution SLA and SLS 3D Printers for Professionals | Formlabs. <https://formlabs.com/uk/?&>;utm_source=google&utm_medium=cpc&utm_campaign=UK-EMEA-Prospecting-Search_Brand-Trademark-Brand-EN-Exact-Paid-Adwords&utm_term=formlabs%20printer&utm_content=formlabs_printers&utm_device=c&_bt=345203181553&_bk=formlabs%20printer&_bm=e&_bn=g&_bg=70222759115&gclid=Cj0KCQjwsp6pBhCfARIsAD3GZuauRLTauENV3-KUHR17vNNe0U026sjAqTjInAnjqoYeaubTq4E4TEcaArxJEALw_wcB.

9. Phrozen Sonic Mini 4K Resin 3D Printer | Phrozen Technology: Resin 3D Printer Manufacturer. <https://phrozen3d.com/products/sonic-mini-4k-resin-3d-printer-phrozen>.

10. Anycubic Photon D2 – ANYCUBIC-UK. <https://uk.anycubic.com/products/anycubic-photon-d2?variant=44183762272541>.

11. Balakrishnan, H. K. *et al.* 3D Printing: An Alternative Microfabrication Approach with Unprecedented Opportunities in Design. *Anal Chem* **93**, 350–366 (2021).

12. Kamei, K. *et al.* 3D printing of soft lithography mold for rapid production of polydimethylsiloxane-based microfluidic devices for cell stimulation with concentration gradients. *Biomed. Microdevices* **17**, 36 (2015).
