# Supplementary material for: Low-cost, versatile, and highly reproducible microfabrication pipeline to generate 3D-printed customised cell culture devices with complex designs
Source: PLoS Biol. 2024 Mar 13;22(3):e3002503. doi: 10.1371/journal.pbio.3002503 (PMC10936828; doi:10.1371/journal.pbio.3002503)
Supplement: Attachment — Submitted filename: Sol3d PLOS Response to Reviewers SUBMISSION.docx [file pbio.3002503.s021.docx]

**Response to the EDITORIAL Team Comments:**

*“As you will see below, the reviewers overall find the manuscript interesting and the method useful. However, they also raise some concerns…”*

*“To address these issues, after discussion with the Academic Editor,* ***we recommend to improve the narrative of the manuscript to make it useful to a non-expert reader along the lines of the suggestions of Reviewer 2*** *and Reviewer 3,* ***including the chemical analysis to ensure that the results can be reproduced by the community****.”*

We would like to take the opportunity to thank the editorial team for their inputs and for giving us the possibility to address the reviewers comments and implement their suggestions to improve our manuscript. We have implemented the suggestions above by including new text, figures, comparisons and detailed guides, as well as shared resources that will be available to the whole community.

**Response to the Reviewers Comments:**

*(Note: the coordinates of the edits and additions provided below refer to the pdf file of the submitted manuscript, starting with the title/authors/abstract page, and do not take into account potential other pages added to the beginning of the document by the editorial submission platform). All new additions within the main manuscript are highlighted in red.*

**Reviewer #1:** We thank the reviewer for their assessment and praise of our manuscript. In their review they found “*the manuscript is well-written with various control and sample tests, and the conclusion is supported by carefully presented experimental data…”,* and that *“the length, figures, and citations are appropriate, and the results presented in this paper are highly interesting.”*  They have also stated that “*Therefore, the readership of this work will be broad*.“ and recommended publication.

One suggestion they make related to the testing of a different brand of printers:

“*One of the popular 3D printers providing large scale and high-resolution printing is the FormLabs 3D printer. Have the authors considered testing this printer and their resins? Other than that, I recommend publishing this manuscript as is”*

Indeed, FormLabs printers are very popular, although -within the realm of consumer-oriented vat printers- relatively more expensive and lower resolution than more recently developed LCD and DLP printers. Nonetheless we have tested already the Formlab resins as well as the printers for our pipeline and found that while the resins were not as successful as moulds for PDMS without treatments, the printers had no issues and with the correct resins and could be integrated within the pipeline.

**Reviewer #2:** We thank the reviewer for the kind words and the positive feedback on our manuscript. We could not agree more that the timing for this kind of study is now, as the technology is available and mature, as that the audience we ought to address is researchers in the biological and biomedical fields that are interested in custom designs but lack the technological or design expertise. For these reasons, we are particularly grateful for the many suggestions aimed at honing the language and increasing the usefulness, targeting this potentially large group of readers. We have implemented all the suggested changes and we believe it has significantly increased the quality of the work.

*“The most significant shortfall for non-technology oriented researchers it its failure to discuss where the designs come from, It does not need to be a full CAD tutorial, but some explanation about where-hoe to draw, file formats, downloadable designs and slicing options are essential to allow new researchers to enter this space.”*

We agree with the reviewer that, upon reflection, although this is intended as a “how to” guide, several aspects of the pipeline do rely on skills and processes we do not necessarily detail for the reader, and that might prevent full adoption and limit impact. In response to this (and the other comments below) we have significantly restructured the manuscript and included two important additions: a) we have now compiled a companion 21 page document, provided in the supplementary files, that constitutes a true step by step “from scratch” guide for the user of the pipeline, and covers in details the different printing technology, principles and tips for CAD design, slicing software and many other topics; b) we have created a GitHub page associated to the guide (<https://github.com/SerioLab/SOL3D> ) were we have collected all the designs used in the manuscript (in an open source parametric and user-modifiable form), together with the detailed manual and links to the preprint.

*“With that, perhaps some hints on hydrodynamic resistance and how design can be used to manipulate flow would aid in the starting up phase. And some information in regard to world to chip interfacing through printing fittings and connectors is also expected to facilitate the journey.”*

While we understand that microfluidic devices are an important portion of the devices used in biology, we have not originally included it in our work because, beside the fact that there are already a number of detailed reviews on microfluidic systems (MCSs) in biology, we felt that of all the possible devices that can be fabricated with SOL3D or other vat polymerisation techniques. In this case the capillary fluidics is probably the one where photolithography is still preferable for several reasons. Nonetheless we agree, upon reflection, with the reviewer that MCSs and other fluidic devices might be one of the reasons some researcher would want to implement this pipeline, so we have now included an example of it within the main text and supplementary figures (**Main text, section title “PDMS bonding for chamber slide devices”, page 14, line 17; Supplementary figure S17A-F**), as well as adding the relevant information in the **Material and Methods (page 26, line 6)** and adding the design to the GitHub repository.

*“While the manuscript is lengthy, it lacks focus and a clearly defined scope. In the introduction, a more comprehensive overview of resin printers could be provided.”*

The reviewer is indeed right that the manuscript became somewhat lengthy and needed to be refocused, so we thank them for their suggestions. As already outlined in above, in order to both be able to provide the additional details and avoid increasing further the length of the main text, potentially decreasing readability, we have opted to streamline the main text, where we provide a brief intro to the main concepts intended to serve as a point of entry to the results, and to instead provide the detailed introduction and comprehensive review within the supplementary guide. In particular, the reviewer can find the **text** addressing this specific point **in pages 1 to 3 (Background) of the supplementary guide**, and within the **main text in page 3, Introduction, line 11 to 32.**

*“Pleaqse note the nanoscribe and Up3D are commercial 2PP systems and that should be mentioned. Also, BMF has launched the S230, 1 2 um pixel, 10 um resolution printer. For the more budget versions, the difference between DLP where a digital light engine is used, to the LCD based printers where light from an LED array is selectively masked by the screen as well as the SLA based systems where the laser writes the design in a layer by layer manner. Advances have been made in all classes, so a brief summary of what printer provides what edge may help readers with the very first step: buying a printer.”*

We agree that a in depth explanation of different printer types was necessary for the beginners, guide, as we felt that adding this part into the main text would result in an even longer introduction and it is not strictly necessary for the implementation of our SOL3D protocol. We therefore added descriptions for different printer types into the 3D printing manual **in pages 1 to 3 (Background) of the supplementary guide** and referenced the suggested commercially available 2PP systems in the **main manuscript Introduction page 4 line 1,** that are further advanced in resolution than standard vat polymerisation but accompanied with significant cost.

*“In the experimental, no information is provided on the key difference between two printers and why they use different resins: the wavelength! This is critical information that needs to be understood before people purchase the wrong resin, or want to blend their own.”*

This factor is now acknowledged in the **supplementary guide on page 1 line 43**, clearly stating *“There are 3 main subtypes of vat polymerisation-based 3D printers, defined by their UV light source, which dictate resin choice (due to light wavelength – extensively reviewed^7^, as SLA printers usually use ~395nm light and LCD screens emit longer wavelength of ~405nm), part resolution, printing time, and printer cost.”* We also refer to a thorough investigation of wavelength absorption in specific resins.

*“I woudl also suggest to refer to the 2016 (9??)Nordin paper in optimizing exposure, for finer detail and custom resins, this is important.”*

We have added this reference within in the **main text, see page 4 line 36 (reference 36)** and in the **supplementary guide, see page 1 line 43 (reference 7).**
 *“The enamel paint is a nice idea, and i would love to understand how it changes morphology through reference SEMs and surface area roughness before coating. It may well be the particles in the paint rather than the 3DP that causes the roughness. The profiler images look nothing like a DPL print, where we expect ~8 um deep pixelation.”*

We agree with the reviewer that this is an important point. We have added an additional characterisation the surface of our moulds after paint deposition, to better show the effect that the airbrushing has on the surface properties **(Fig. S7, also represented below for convenience)**. We initially provided in the submitted version of the manuscript figure S7 with a SEM characterisation of the paint layer thickness and described the layer as “<100um”, which is an overestimation of its actual dimensions. We have now corrected that based on the analysis of the thickness visible in the SEM images (Fig. S7B), now presented as “(between 30 and 50 µm)”, which more faithfully represents the situation.

On the subject of the pixelation/visible LED units within the prints, we do not observe in any of the printers “~8 um deep pixelation” across 4 different brands and models, which would be problematic for the scale of features we demonstrate, and in our experience the current generation of LCD high resolution (18-36 um pixel size) do not tend to show such prominent artefacts. We do however observe smaller pixel effects on our prints, **as shown now in figure S7 E and F (see below)**. Several of these printers have specific antialiasing options and other grey scale control systems to lower the pixelation and layer visibility, in some cases directly written within the firmware, or more often specified within the slicing software. Moreover, in some cases (e.g., Phrozen 8k) the pixel matrix is not directly below the vat, but instead the light is focused and transmitted via an optical system, which allows the printers to further smooth the pixelation and layering.

**Figure S7: Analysis of paint layer thickness and surface roughness**

(A) Representative SEM image of a resin A print with airbrushed enamel paint (B) Analysis of paint layer thickness on 3D vat printed moulds (C, D , E, F) Optical profiling of a 3D print (C) painted with enamel, shows a surface roughness that is variable around 3um (D) a flat silicon wafer shows variability in the nanometer scale (E, F) a non-painted 3D print showing a small variation based on the pixel size of the screen, with 1um variability.

One of the areas where we agree with the reviewer that pixel size and pixelation effects become crucial, is when printing patterns of features in the approximate scale and spacing of the single pixels or pixel clusters. We have now expanded and thoroughly tested this eventuality within the in-depth guide, using the printing of grooves (depth of 200µm and width of 100 µm) as an example. Our testing shows the potential interference patterns between the repeating features and the pixel alignment on the LCD matrix (see below and within the supplementary document page 15), which can be adjusted by changing the angle to the LED array at which the device is printed. In this scenario, when the pixelation effect is most noticeable (0 degrees) one can indeed observe 8 to 10 um deep breakages within the features that correspond to single pixels.

*“The PDMS casting study is interesting, and needs a reference to Folch' paper on 3DP PDMS. There are many reports casting from DLP moulds, so an expression what is new and what matches precedence is helpful.”*

We have added this information within in the **main text, see page 20 line 50.**

*“It is well established that biocompatibility strongly depends on cell type and experiment, and there are some reviews looking at this in detail that could be referenced.”*

We have added this information within in the **main text, see page 2 line 43**.

*“I would like to suggest to expand the introduction to understand printer, drawing and design and resin selection, while shortening the culture examples to make them nice illustrations of what can be achieved.”*

As detailed in the responses above, the main manuscript has now been shortened and the necessary details on resins, printing and CAD added to the in-depth guide. We feel that with these changes there is a better balance between the biological applications (which we believe should be detailed to showcased what kind of biological questions can be addressed with these approaches) and the necessary technical details.

**Reviewer #3:** We thank the reviewer for their comments and suggestions.

“*The main downsides are a worse spatial resolution (~50-100 µm minimal feature sizes in SLA) and some unknowns pertaining to biocompatibility.”*

Regarding the resolution, throughout the entirety of the work presented here we employ printers that have a minimal feature size in X-Y between 18 and 36 µm, and 10 µm in Z. Therefore, while we agree with the reviewer that photolithography can achieve higher spatial resolution (and acknowledge this several times throughout the main text and now supplementary guide), the characterisation of the minimal feature size given here is not entirely representative of current technology SLA/DLP printing (e.g., see BMF brand printers, with resolution down to 2 µm) nor of the work we present. Moreover, while it is true that the applications, we showcase make use of features in that scale range (chosen because they are representative of real use cases within biological experiments), the pipeline is capable of much smaller features. For example, in this version of the manuscript, in response to a suggestion from another reviewer, we have included a microfluidic device with channels of 9 µm in height, using a design normally fabricated with conventional photolithography (**see Figure S17**).

Regarding the “unknowns pertaining to biocompatibility”, while certainly that was exactly the starting point of our investigation, we have performed extensive experiments to test the biocompatibility of our protocol with stem-cell derived neurons and other cell types, and compared the biocompatibility of a number of different resins (**See Figures S2,S3 and S6**, already included in the reviewed manuscript) which goes above and beyond what normally is tested for conventional soft-lithography in PDMS, specifically to ensure that highly complex, long term biological experiments with sensitive cell type could be conducted safely. We are confident that the work presented (now enhanced by the implementation of the reviewers’ suggestions) demonstrates the biocompatibility of the devices made with this pipeline.
 *Several other groups have investigated and reported such a workflow. A common challenge is the inhibition of PDMS curing by residual small molecules and oligomers in the resin. Incomplete PDMS curing can have severe impact on biocompatibility, and resin residuals may similarly be cytotoxic, which is combatted through extensive cleaning (washing) and coating of the SLA parts. In light of the earlier work within this space, the novelty of this work is somewhat limited.*

We agree with the reviewer that the idea of using 3D printing to produce PDMS moulds has been within the field for some years, and several different reports are published with different levels of success, detail and applicability. As such, we do not claim the idea itself is novel anywhere in the manuscript, and we reference several other works that have used different methodologies in the introduction, as background and steppingstones in our optimisation. In our opinion the value and novel proposition, (and where we believe this work represents a useful resource) lies on the first complete pipeline (to our knowledge) that has been optimised specifically for biological labs, that require virtually no access to expensive or difficult to source equipment (e.g., parylene coaters, mask aligners, and even plasma cleaners as we detail in the material and methods and the supplementary guide), can be set up with no prior expertise and minimal upfront cost, and has been described across several different biological applications. We believe that this pipeline will make these powerful techniques more accessible to non-specialist labs across a variety of fields. Hence why we propose will be a good fit for the Methods & Resources section of a journal focused on a biology-oriented readership, rather than a to engineering, nanoscience or microfabrication experts who have access and expertise to already fabricated such devices to um and nm precision.

Moreover, the work described here was born of our need to customise quickly cell culture devices in large quantities within a biology wet lab, precisely because the testing of the already available and published pipelines resulted in limited applicability for various reasons.

*“In my view, the main finding of the paper is of practical nature, in the identification of a commercial resin for SLA printing that does not impair PDMS curing notably, and where PDMS parts are fully biocompatible within the tested ranges. However, the reasons why this particular resin is advantageous is not investigated. The paper therefore does not provide any mechanistic insights into the choice or formulation of resin for such purposes.*

We respectfully disagree on the characterisation of the “main finding” being only the identification of a commercial resin with interesting properties. What we present is a framework to test and select the potentially useful resins for the pipeline and a post processing protocol that works across a wide range of compositions, which will ultimately, in our opinion, allow different labs to test and optimise a variety of different resins and fabricate their custom devices.

As we point out in the text, some resins have properties that better suits the pipeline, while others require more extensive post processing, and others still are virtually unusable, but nowhere in the manuscript we suggest that one specific resin should be used over the others, as many labs will likely have accessibility to different manufacturers and different resin compositions, and indeed composition can be altered over time by the manufacturers.

We agree that our manuscript does not address the formulation of these proprietary resins, as this poses a number of issues and difficulties (**see response to Comment 1 below**) and we think this goes beyond the scope of this work, i.e., to provide a pipeline any biologist could optimise and adopt. Customised resin composition, and detailed chemical forensic analysis of commercial resins are unlikely to be steps widely adopted by the intended target audience. Nonetheless we have performed the analysis of some of these resins as the reviewer requests, see response to Comment 1 below for details.

*“I understand that this can be challenging, since the applied SLA resins are commercial products, but perhaps some chemical analysis would return insights as into why this resin is more compatible than the other tested resins.”*

See response **to Comment 1 below**.
 *The paper also presents a somewhat workable solution to applying other SLA resins, through coating the printed parts with enamel paints using airbrush. However, this procedure to me seems to not be very reliable for complex e.g. non-planar designs, in particular being a manual process, with the variability this brings. Lastly, even if reproducible, the application of a 30um coating will be problematic for achieving high-resolution features.*

We apologise that our description of the method was not clear. As detailed in Figure 1G, the pipeline protocol has an optional step for enamel coating that specifically is intended for large devices or fully enclosed moulds (e.g., muscle tissue engineering devices from Fig.7) that do not require high resolution features, while the non-coated postprocessing moulds are recommended for high-resolution features. See below:

In the **original submission** we discuss this, specifically in page 7, line 1:

‘Analysis showed that coated prints exhibited a thin layer of paint (between 30 and 50 µm) and greater surface roughness compared to uncoated ones **(Figure S7)**. This limits the application of this method to features larger than 100 µm in any dimension **(Figure 1F)**.’

As one example of the fact that enamel coating is not needed for high resolution features, we can point to the newly included **Figure S17**, which features a microfluidic device with channels 9 µm in height that have been casted on an uncoated processed resin mould.

Regarding applicability of the coating to complex non-planar devices, it is true that particularly complex shapes with potential “blind volumes” within the mould that are not easily accessible could be challenging to reach with the enamel paint. However, as the same challenge would apply in their use as a PDMS mould, this is unlikely to represent a significant limitation. For example, the devices used in the muscle tissue engineering in figure 7 have a complex geometry and would not be able to be moulded in a single device, which is why the printed moulds are split in 2 parts, which automatically allows for ease of access with the airbrushing technique. To further address this point, we explain in depth the airbrushing protocol in the **new supplementary guide, see page 21**, and give example designs to choose which pipeline to use.
 *Although the engineering and materials analysis could be improved, the data presents and comprehensive set of potential applications of the workflow, spanning several types of cells and several types of devices. The biological data is generally convincing and of good quality. The presented procedure might therefore have some impact for the creation of prototypes in research lab, even if it boils down to other groups simply applying the same resin.*

We would like to thank the reviewer for the positive assessment and their suggestions on improving the engineering and the materials analysis, which we have implemented in full as detailed in the response to their other comments. Together with the other reviewer’s suggestions we believe the revisions have significantly improved our work, and we believe it will have a larger impact than simply other groups adopting one specific resin for their printers.

*“Comment 1: A more in depth analysis of resins content would potentially enable a discussion of why certain resins apparent work better with PDMS molding”*

We have analysed the commercial resins via ^1^H NMR, as suggested by the reviewer, to try and gain an insight into their composition. As the reviewer highlights, this is a challenging thing to do as the resins are complex mixtures of many components (up to 10 for some resins) at varying and unspecified ratios, with the nature of these components and their formulation being proprietary information. From this analysis, while we can glean small insights into the main component of several of the resins, any correlations between the composition and their performance were small and we are therefore wary of drawing any conclusions or inferrals from this analysis. We therefore provide below a summary of our analysis, but do not think this is well suited to being included in the main manuscript, particularly in light of the potential legal implications of ‘reverse-engineering’ a proprietary product. However, this decision is at the discretion of the Editor if they would prefer this information to be included.

From our analysis, we can make some general observations. The 4K resin is composed primarily of an acrylate monomer bearing an electron donating substituent, e.g., an acrylamide. This monomer is shared by both the Clear and Water washable resins. However, these are both mixtures with either an aromatic monomer (Clear resin) or an electron deficient acrylate (Water washable) which may significantly alter their properties. The electron deficient acrylate is also shared with Flex resin, which has 3 major components rather than two. Of the remaining resins, both Tough and Ortho are composed of a common component that we have been unable to identify. Notably, of the resins used, 4K, Tough, and Ortho seem to be composed predominantly of a single component which may contribute to their observed properties, while the others are a blend of monomers.

| Resin | Fragments |
| --- | --- |
| 4K | **A** |
| Clear | **A**, B; A:B=1:1 |
| Flex | B, **C**, **D**; C:B=4:1 |
| Tough | **D** and unidentified aromatic fragment |
| Water washable | **A**, **C**; A:C=1:1 |
| Ortho | **D** |

*Comment 2: The manuscript several times states "high-resolution" about the procedure, apart from being highly qualitative statement, I would also argue that in the context of soft lithography, that the resolution is not high but poor. To settle such matter, why not simply write that smallest possibly feature sizes (in the order of 100um seems like a fair statement, so "sub-millimeter")*

The majority of the uses for “high-resolution” within the manuscript refers to the 3D resin printers, as it is common to refer to the actual pixel resolution of the LCD screens that enable the feature size (e.g. all the printers we tested have a minimum of 4K native resolution, 2840x3460 pixels), and we do state the ranges for the minimal features early on in the introduction (**see Page 3, Line 17**).

We also fully acknowledge within the introduction (and now within the initial section of the supplementary in-depth guide) that photolithography is currently capable of higher precision and smaller feature sizes (**see Page 3 starting at line 11 in the main text and Page 1 of the supplementary guide**).

To avoid confusion, we nonetheless removed “high-resolution” from the title and the abstract.

*Comment 3: The evaluation of PDMS curing seems highly qualitative. I would suggest confirming full crosslinking by extended swelling in a good solvent (Hexane perhaps) and evaluate mass of oligomers or monomers that have leached from part.*

As suggested by the reviewer, we have performed such an experiment. Samples were incubated in an excess of n-hexane for 24 h to allow swelling, and the mass of oligomers or monomers that leached out was then calculated. We also calculated the mass of the samples after air drying, and all experiments were performed in triplicate. We found only very minimal weight loss from PDMS samples, within the margin of experimental error, and can therefore quantify PDMS curing as being >97%, analogous to previously reported figures for PDMS devices. Details of this have been added to the **main manuscript, see Material and Methods page 23 line 6**.

In addition to this new data, it is worth highlighting that in the original manuscript we included a full Raman spectroscopy analysis of the different curing states at different stages of the pipeline, comparing it with conventional methods (**see Figure S4**). Together with the cell survival analysis and long-term cultures, these results strongly support our statement that we are obtaining cured devices with our set-up, indicating that the SOL3D moulds perform as well as conventional photolithography moulds for curing.

*“Comment 4: rather than testing biocompatibility be seeding of cells a more convincing argument that the PDMS parts do not leach monomers or resin components would be to subject to heated water and apply analystical chemistry methods e.g. mass spec to verify that such components are not found in the media.”*

We have performed the experiment suggested by the reviewer, incubating PDMS devices fabricated in SOL3D moulds in water at 37 °C for 72 h, to mimic the conditions of the biological experiments that would be performed. We then undertook MALDI-TOF mass spectrometry analysis of the leachate, as described in the reference suggested by the reviewer. MALDI-TOF MS is a highly sensitive technique can detect the presence of even very trace amounts of dimethylsiloxane oligomers which are known to be the predominant leachates during such experiments. However, we were unable to observe any such leachates, and the spectra are comparable with what observed in the water-only control. This is in line with our original hypothesis that leaching is negligible and supports our observations of high biocompatibility during the long-term analysis and culture of extremely sensitive cell types (human iPSC derived neurons and others) which show no sign of toxicity even after weeks of culture.

It is likely that in this case the focus of the reader might influence what type of data is more convincing, and we are therefore grateful for the opportunity to add this additional bit of data for the broad readership of this journal and can be found in the main manuscript **Figure S5**, were we show the spectra of MALDI matrix-only control, water only (as a control for the PDMS) and water incubated with PDMS as described above.

*Comment 5: Introduction is very qualitative. I would suggest getting more specific and quantitative on e.g. spatial resolution and concept, in last paragraph of introduction*

See response to Reviewer 2 above, we have restructured the introduction and added an entire supplementary document to balance readability and add all the necessary level of detail. We believe that these changes address the points to raised by both reviewers on the focus of the initial sections of the manuscript and their accessibility to a non-specialist audience. *Comment 6: Airbrush technique does not seem to be applicable for all types of designs; I suggest you elaborate and/or document limitations.*

See above, we have addressed this in response to one of the earlier points from this reviewer.

*“Comment 7: It is argued several times that by getting rid of clean-room produced wafer molds the workflow is compatible with most any lab. However, however in addition to handling PDMS resins, which are not benign, the SLA workflow introduces several flammable, light sensitive, and toxic chemicals. Meaning at least a full chemistry setup with hood and chemical waste handling.”*

We appreciate the reviewer’s point of view on this, and acknowledge that one of the challenges with highly interdisciplinary research is that very often labs have different accessibility to different expertise and equipment, therefore a generalisation would always fall short of addressing all possible combinations. Our argument in response to this would be that in theory *wet* labs (i.e. we fully acknowledge that dry labs and other non biologically/biomedically oriented research teams might have different experiences) will in the almost totality of the cases have access to a chemical hood or similar, to perform a number of tasks related to biological experiments and entail the handling of chemicals far more problematic than PDMS or 3D printing resins (to provide some examples, Paraformaldehyde and other fixatives, DMSO, phenols or other derivatives employed in molecular biology, etc.). Given that the risk profile for the components of the pipeline is much lower than some of these common chemicals we do not agree with the characterisation that a full chemical set up is needed, at least not one that goes beyond what any wet lab that performs cell cultur

e experiments (and therefore might have a need for device fabrication as described) would not already have (i.e. a chemical hood and a basic chemical waste management system).

Regarding the additional safety requirement introduced by the resin printing, all of the printers we have tested, and the majority of commercially available printers, are intended primarily for “at home” and sold with a risk profile that can be easily implemented within private homes and DIY spaces, rather than research labs and generally require only good ventilation when handling alcohol based washing steps or the resin themselves, which is likely a much lower safety standard than any research lab maintains as baseline.

*“Along these lines, I would argue that the main time-consuming step in in traditional soft lithography is not producing the wafer, since the wafers are generally reused multiple times. To me, the main time consuming and impractical steps, relates to PDMS mixing, degassing, curing, demolding, cutting, bonding etc, which is still needed for the presented procedure. But perhaps that's the eye of the beholder.”*

Different teams/labs will have different experiences of what constitutes “bottlenecks” within their working pipelines, so we cannot comment specifically on this other than agreeing that in general there is a large component of handling and processing required for these experiments. However, we absolutely agree that the“time consuming and impractical steps” mentioned (e.g. demoulding, cutting, bonding, degassing etc.) are exactly the same whether one uses photolithography moulds, CNC milled moulds, resin printed or any other system, and as such -given the resin based printing is faster per mould, and with higher overall throughput potentially (i.e. multiple different devices for each print, multiple printers running at the same time)- SOL3D still represents a significant reduction in the production time and prototyping turnaround.

*Comment 8: The limitations/opportunities for reusing SLA templates several times, could be investigated.*

We agree with the reviewer on this point. We have already provided in the original manuscript a full characterisation of the effects of reusing serially the same mould for casting multiple devices (see **Figure S9C**), where we compare the surface and overall device dimensions of 25 repeated casts, but have now explicitly pointed this out with pros and cons in the discussion **(see main text Page 20, line 17)**

*Comment 9: In the last page of section 2.2 you mention "volumetric" printing. Unless I am mistaken only traditional layer by layer SLA printing was performed (?).*

We apologise for the confusion, in this case we intended to mean *“**producing multiscale features simultaneously”* but indeed the reviewer is correct that on reflection it might be misleading, and we have therefore removed that.

*Comment 10: Figures. Figure 1C: Numbers on optical profiling data extremely hard to read, 1F: Couldn't find the arrows mentioned in caption,*

We apologise for the mistake; we have now corrected the figure accordingly.
